# New perspectives on body size and shape evolution in dinosaurs

**DOI:** 10.1111/brv.70026

**Published:** 2025-05-08

**Authors:** Matthew Dempsey, Samuel R. R. Cross, Susannah C. R. Maidment, John R. Hutchinson, Karl T. Bates

**Affiliations:** ^1^ Department of Musculoskeletal & Ageing Science Institute of Life Course & Medical Sciences, University of Liverpool, The William Henry Duncan Building 6 West Derby Street Liverpool L7 8TX UK; ^2^ Fossil Reptiles, Amphibians and Birds Section, The Natural History Museum Cromwell Road London SW7 5BD UK; ^3^ School of Geography, Earth and Environmental Sciences, University of Birmingham Edgbaston Birmingham B15 2TT UK; ^4^ Structure and Motion Laboratory, Department of Comparative Biomedical Sciences Royal Veterinary College Hatfield AL9 7TA UK

**Keywords:** dinosaurs, body shape, body size, centre of mass, volumetric modelling, macroevolution

## Abstract

Diversity in the body shapes and sizes of dinosaurs was foundational to their widespread success during the Mesozoic era. The ability to quantify body size and form reliably is therefore critical to the study of dinosaur biology and evolution. Body mass estimates for any given fossil animal are, in theory, most informative when derived from volumetric models that account for the three‐dimensional shapes of the entire body. In addition to providing estimates of total body mass, volumetric approaches can be used to determine the inertial properties of specific body segments and the overall distribution of mass throughout the body, each of which are essential for the modelling and interpretation of form–function relationships and their associations with ecology. However, the determination of body volumes in fossil taxa is often subjective, and may be sensitive to varied artistic inference. This highlights the need for an approach to body mass estimation in which body segment volumes are systematically constrained by quantitative scaling relationships between the hard tissues that fossilise and the soft tissues only observable in extant taxa. To this end, we used recently published skeletal to soft tissue volumetric scaling factors derived from CT data of extant sauropsids to estimate body segment mass properties from skeletal models of 52 non‐avian dinosaurs representing the majority of major clades and body plans. The body masses estimated by this study range from less than 200 g in the tiny avialan *Yixianornis* to over 60 tonnes in the giant sauropod *Patagotitan*, which is currently the largest dinosaur known from mostly complete skeletal remains. From our models, we infer that many previous reconstructions of soft tissue envelopes may be too small, and that many dinosaurs were therefore heavier than previous estimates. Our models generally overlap with the range of body mass estimates derived from limb bone shaft dimensions, but with considerable quantitative variability among major clades. This suggests that different taxa either differed in skeletal to soft tissue volume ratios, or that their limb bone dimensions varied relative to body mass, perhaps related to differences in locomotor dynamics and postural evolution. Our models also allowed us to investigate variation in mass distribution and body proportions across different dinosaurs from a perspective grounded in extant anatomical data, framing long‐standing hypotheses about their form, function, and behaviour in a quantitative context. For example, reconstructed disparity in whole‐body centres of mass reflects a broad array of postures in different dinosaur clades, while the lack of strong positive allometry in the dimensions of the weight‐bearing limb segments relative to total body mass corroborates previous studies suggesting an overall decrease in dinosaur locomotor performance as body size increased.

## I. INTRODUCTION

Body mass is a foundational metric for the study of all aspects of animal biology, being closely tied to geography, ecology, form, function, and physiology (LaBarbera, 1989). The mass properties of a given animal body are tied to functional and behavioural parameters. For example, the lengths and masses of limb segments relative to total body mass are key to the interpretation of locomotor performance and energetics (e.g. Biewener, 1989a; Christiansen, 2002; Hutchinson, 2004; Kilbourne & Hoffman, 2013), the dimensions of the torso aid interpretations of diet (e.g. Clauss *et al*., 2016; Maher *et al*., 2022), and mass distribution throughout the body is tied to posture, locomotor mode, and the ways in which limbs are loaded (e.g. Biewener, 1989b; Alexander, 2003; Allen *et al*., 2013; Bishop *et al*., 2020; Macaulay *et al*., 2023). On a broader scale, diversifications in body size and shape are a distinctive feature of major adaptive radiations (e.g. Benson *et al*., 2014, 2018) eliciting great interest in the evolution of animal body mass properties across deep time.

**Fig. 1:**
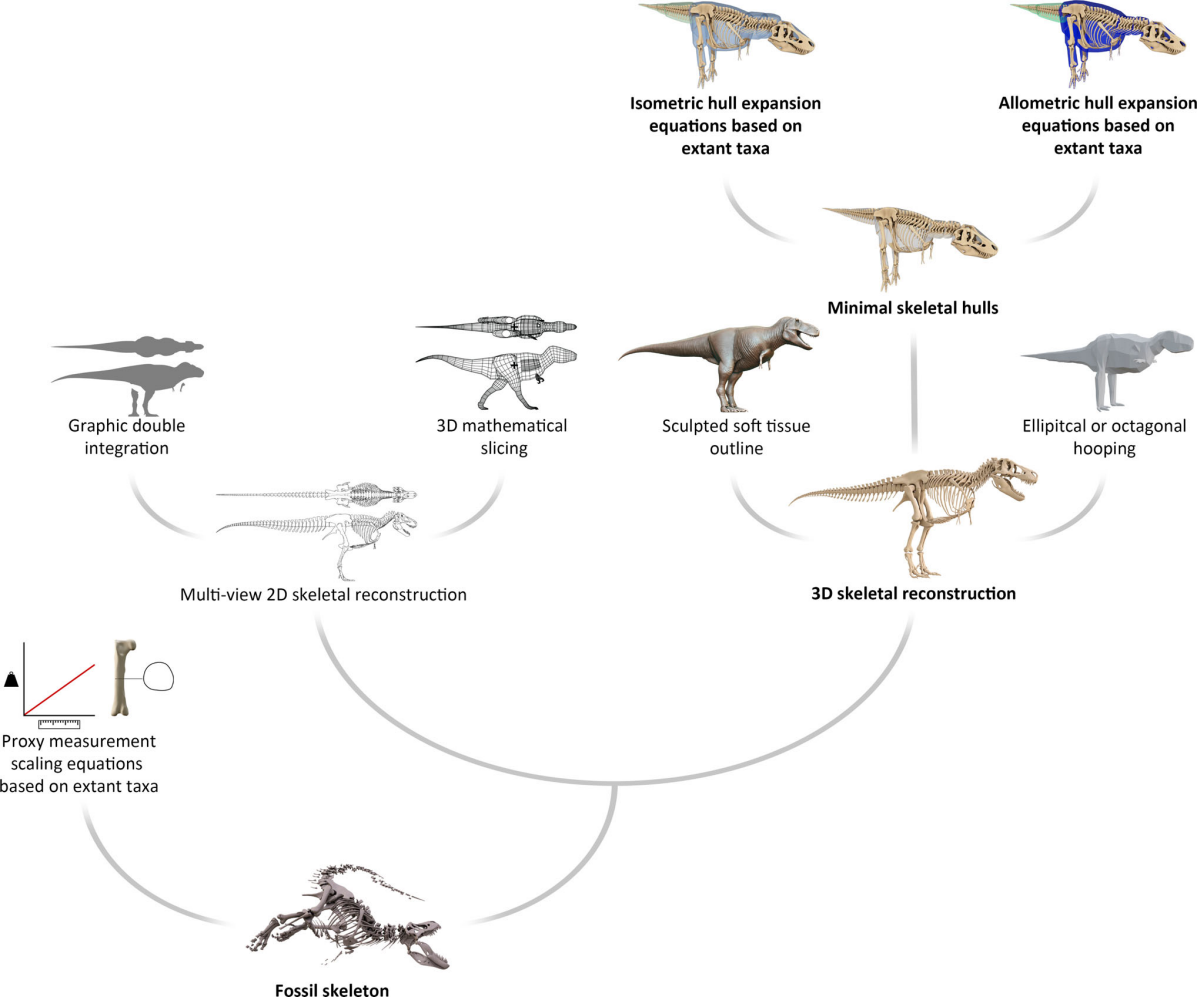
Flowchart illustrating different methods of estimating the body mass of a fossil animal from skeletal remains, using *Tyrannosaurus* as an example. Branches with bold text represent the workflow of this study. The 3D mathematical slicing model is from Henderson (2018) (CC BY 4.0 *via* PeerJ), and the octagonal hooping model is from Allen *et al*. (2013) (CC0 1.0 *via* Dryad).

Given that the *in vivo* body mass of a fossil animal is not directly observable, many methods have been devised to reconstruct body mass in fossil taxa (Fig. 1), with a particular focus having been placed on large tetrapods, especially dinosaurs. Approaches range from the use of statistically robust scaling relationships between limb bone dimensions and body mass (e.g. Anderson, Hall‐Martin & Russell, 1985; Campione & Evans, 2012; Campione *et al*., 2014) to holistic attempts at digitally reconstructing the volumetric dimensions of the entire body (e.g. Henderson, 1999; Bates *et al*., 2009a; Hutchinson *et al*., 2011; Sellers *et al*., 2012; Allen *et al*., 2013; Brassey, 2017; Macaulay *et al*., 2023). Different methods of estimating body mass present varied advantages and disadvantages. For example, while limb bone scaling relationships are very useful for demonstrating major macroevolutionary trends in body mass across large sample sizes (e.g. Benson *et al*., 2014, 2018), they cannot account for the varied distribution of mass between specific body segments. While digital volumetric models provide more holistic information about the form of the entire body, they may be prone to a high degree of subjectivity in the determination of both total body volume and relative shapes (Bates *et al*., 2009a,b, 2016; Bates, Benson & Falkingham, 2012; Allen, Paxton & Hutchinson, 2009; Allen *et al*., 2013; Hutchinson, Ng‐Thow‐Hing & Anderson, 2007; Hutchinson *et al*., 2011).

Focused interpretations of form, function, biomechanics, and physiology in fossil animals must address potential sources of sensitivity and subjectivity across different methods of body mass reconstruction. In this study, we review previous methods of body mass estimation in extinct tetrapods, highlighting their utility in the context of studying the evolution of form and function. We then estimate plausible body segment mass properties in a representative range of non‐avian dinosaurs by using the recently published volumetric convex hull expansion approach of Macaulay *et al*. (2023), which defined segment‐specific scaling relationships between the skeletal dimensions and soft tissue dimensions of extant sauropsids. We compare our models to previous body mass estimation approaches, and discuss variation between methods from anatomical and biomechanical perspectives. The modular construction of our models then allows us to open up broader discussions about the evolution of body dimensions and disparity in form–function relationships across Dinosauria as a whole.

## II. REVIEWING METHODS OF ESTIMATING BODY MASS IN FOSSIL TETRAPODS

A common approach to the estimation of body mass in fossil taxa is to use predictive mathematical relationships between body mass and skeletal proxy metrics defined from extant taxa. In terrestrial tetrapods, these metrics are typically derived from the long bones of the limbs, as their weight‐bearing function suggests adaptational associations between their dimensions and total body mass (Anderson *et al*., 1985; Campione & Evans, 2012; Campione *et al*., 2014). Campione & Evans (2012) demonstrated that the summed circumferences of the proximal limb bone shafts are the most statistically robust proxy metric for body mass in quadrupedal tetrapods, with a theoretical conversion for bipedal taxa based on femoral circumference alone subsequently proposed by Campione *et al*. (2014). This objective scaling relationship is remarkably consistent across a large diversity of extant tetrapods, and thus has high utility in the prediction of overall patterns of body mass evolution across very large data sets of extinct tetrapod taxa (e.g. Benson *et al*., 2014, 2018; Campione & Evans, 2020). While body mass estimates based on limb bone scaling relationships meet a high standard of accuracy informed by extant taxa, they present some limitations. They do not, for example, contain information about how mass is variably distributed throughout specific segments of the body, which can make interpreting possible form function differences between taxa of equivalent body mass challenging.

The other common approach to estimating body mass in fossil tetrapods is to construct whole‐body three‐dimensional (3D) reconstructions. In theory, an accurately proportioned 3D reconstruction allows the volumetric properties of a given taxon to be calculated directly and holistically. However, as with proxy‐based predictive approaches, the different methods of producing 3D reconstructions present varied advantages, disadvantages, and their own sources of uncertainty.

Several 20th century attempts to estimate body mass in extinct animals were based on the volume displacement of scaled physical models (e.g. Gregory, 1905; Alexander, 1985; Colbert, 1962; Paul, 1997), while more recent increases to the capabilities and accessibility of computer modelling technology have allowed many researchers to produce digital volumetric reconstructions [Brassey (2017) and references therein]. Digital models benefit from the ability to quantify easily, precisely, and repeatably the inertial properties (e.g. mass, centre of mass) of reconstructed bodies, and also facilitate the spatial manipulation of individual body segments. For this reason, they have high utility for the study of functional morphology and biomechanics. Digital models can, for example, allow interpretation of how limbs may be loaded during stance and gait (e.g. Henderson, 2006; Sellers *et al*., 2017), aid in the reconstruction of plausible postures (e.g. Gatesy, Bäker & Hutchinson, 2009), and can illuminate other form–function relationships. Multiple studies have used estimated limb segment volumes to infer a lack of strong positive allometry in the limb muscle masses of theropod dinosaurs, suggesting that locomotor performance was not maintained across ontogenic and evolutionary increases in body size (Bates *et al*., 2012; Hutchinson *et al*., 2011). Based on similar data, other studies have hypothesised that changes in relative body segment lengths, estimated masses, and estimated centres of mass of different sauropodomorphs and ornithischians were associated with both ontogenic and evolutionary changes to their locomotor form (e.g. Henderson, 2006; Bates *et al*., 2016; Maidment, Henderson & Barrett, 2014b; Otero *et al*., 2019). Modular volumetric models also facilitate the modelling and simulation of dinosaur locomotion (e.g. Sellers *et al*., 2017; Bishop *et al*., 2021c), feeding actions (e.g. Bates & Falkingham, 2012; Snively *et al*., 2013), physical behaviour when submerged in water (Mallon *et al*., 2018; Sereno *et al*., 2022), and other biological actions. Volumetric approaches to body mass estimation, whether physical or digital, are founded on assumptions of overall density, which is often either modelled as equivalent to water (1 g/cm^3^), derived from measured densities in extant animals (e.g. Brassey & Sellers, 2014; Macaulay, Hutchinson & Bates, 2017), manually reconstructed with zero‐density air spaces based on the comparative anatomy of extant taxa (e.g. Henderson, 1999, 2006, 2014, 2018, 2023; Bates *et al*., 2009a,b, 2016; Hutchinson *et al*., 2007, 2011; Allen *et al*., 2009, 2013; Larramendi, Paul & Hsu, 2021), or based on segment‐specific overall densities (e.g. Maidment *et al*., 2014b; Macaulay *et al*., 2023).

Many methods have been used to produce digital volumetric models of fossil taxa. One of the more technologically simple approaches is graphical double integration (GDI) (Jerrison, 1969), in which the estimated volume of a given animal can be extrapolated from multi‐view two‐dimensional (2D) outlines (e.g. Hurlburt, 1999; Christiansen & Bonde, 2002; Larramendi *et al*., 2021). The 3D‐slicing approach of Henderson (1999) and many subsequent studies (e.g. Henderson, 2006, 2014, 2018, 2023; Maidment *et al*., 2014b; Mallon *et al*., 2018; Snively *et al*., 2019) is a conceptual evolution of the GDI approach, in which multi‐view 2D outlines based on skeletal or life illustrations are used to generate a three‐dimensional (3D) reconstruction composed of elliptical segments. The generation of 3D volumes from the slicing of multi‐view 2D outlines was further elaborated on *via* the usage of superelliptical cross sections by Motani (2001). Other 3D volumetric modelling approaches use skeletal geometries more directly, constructing soft tissue outlines around 3D skeletons derived from either fossil scan data or accurately sculpted models. The complexity of manually defined soft tissue outlines constructed around 3D skeletons varies among studies, from surfaces generated *via* the hooping of elliptical or octagonal primitives (e.g. Gunga *et al*., 2008; Allen *et al*., 2009, 2013; Bates *et al*., 2009a,b, 2012; Hutchinson *et al*., 2011; Mallison, 2010, 2014; Otero *et al*., 2019) to detailed and naturalistic artistic sculptures (e.g. Ibrahim *et al*., 2020; Rovinsky *et al*., 2020; Romano *et al*., 2021; Sereno *et al*., 2022; Atkins‐Weltman, Snively & O'Connor, 2021; Larramendi *et al*., 2021).

Irrespective of whether or not they benefit from using 3D skeletal models directly to guide the reconstruction of body volumes, each of these approaches shares the common drawback that soft tissue outline reconstruction is highly subjective in fossil taxa – even if complete skeletons can be accurately assembled with confidence, estimates of body mass are ultimately only as accurate as the reconstructed soft tissues enveloping them. Several studies have addressed this subjectivity by expanding soft tissue outlines from a proposed minimal model to varied extremes, producing a range of possible body masses (e.g. Allen *et al*., 2009, 2013; Bates *et al*., 2009a,b, 2012; Hutchinson *et al*., 2007, 2011; Otero *et al*., 2019). However, with the exception of tail reconstruction workflows (e.g. Allen *et al*., 2009; Mallison, 2011; Snively *et al*., 2019), the margins of uncertainty set by these plausible volume ranges are still largely subjectively inferred rather than being objectively generated from biological data. Based on the observation of skeletons alone, the biologically feasible minimal and maximal extents to which soft tissue outlines should adhere to skeletal landmarks are unavoidably subjective, and results may vary among investigators (Bates *et al*., 2009a; Hutchinson *et al*., 2011). *Tyrannosaurus*, as a popular candidate for studies of body mass, provides a clear case study of this subjectivity. Depending on the size and shape of the soft tissue envelope estimated by different researchers, body mass estimates based on volumetric models of adult *Tyrannosaurus* (~11–12 m in length) range from less than 6 tonnes to over 18 tonnes (e.g. Henderson, 1999; Bates *et al*., 2009a; Hutchinson *et al*., 2007, 2011). Subjectivity in the definition of soft tissue outlines also raises difficulties in the accurate estimation of whole‐body centre of mass, which is a particularly important metric for the inference of locomotion and posture (e.g. Gatesy *et al*., 2009; Allen *et al*., 2013; Maidment *et al*., 2014b; Macaulay *et al*., 2023). Without systematic limitations placed on the specific volumes of each body segment, determining how overall mass is distributed throughout the bodies of fossil animals is challenging, resulting in a variable spectrum of estimated whole‐body centres of mass (e.g. Allen *et al*., 2009, 2013; Hutchinson *et al*., 2011; Bates *et al*., 2016).

In order to estimate volumetrically reconstructed masses in fossil taxa with greater objectivity, several recent studies have attempted to use extant taxa to quantify relationships between the hard skeletal tissues more prone to fossilise and the soft tissues that envelope them. Sellers *et al*. (2012) regressed the calculated masses of mathematically derived minimal convex hulls formed around 3D scan data of mammal skeletons against body masses based on known scaling relationships from the literature, finding that the minimal hulls consistently underestimated body mass by approximately 21%. This convex hull workflow represents a hybrid approach to body mass estimation, integrating mathematically derived relationships in extant taxa with the complexity and more direct approach of whole‐body 3D modelling, and has since been iterated upon in many subsequent studies (e.g. Bates *et al*., 2015, 2016; Brassey & Sellers, 2014; Brassey, Maidment & Barrett, 2015; Brassey *et al*., 2016; Sellers *et al*., 2017; Otero *et al*., 2019; Coatham, Sellers & Püschel, 2021; Macaulay *et al*., 2023; Wright, Cavanaugh & Pierce, 2024). Initial applications of the convex hull and other automated shape‐fitting workflows such as alpha shapes (e.g. Brassey & Gardinier, 2015) were based on the relationship between total minimal skeletal volume and total body mass. While allowing for objective, systematic estimation of total body mass in fossil taxa, this workflow does not apply constraints upon the differential distribution of total volume across the body. As with previous methods, the inertial properties of each individual body segment and the resultant whole‐body centre of mass therefore remain subjective (Bates *et al*., 2016).

**Fig. 2:**
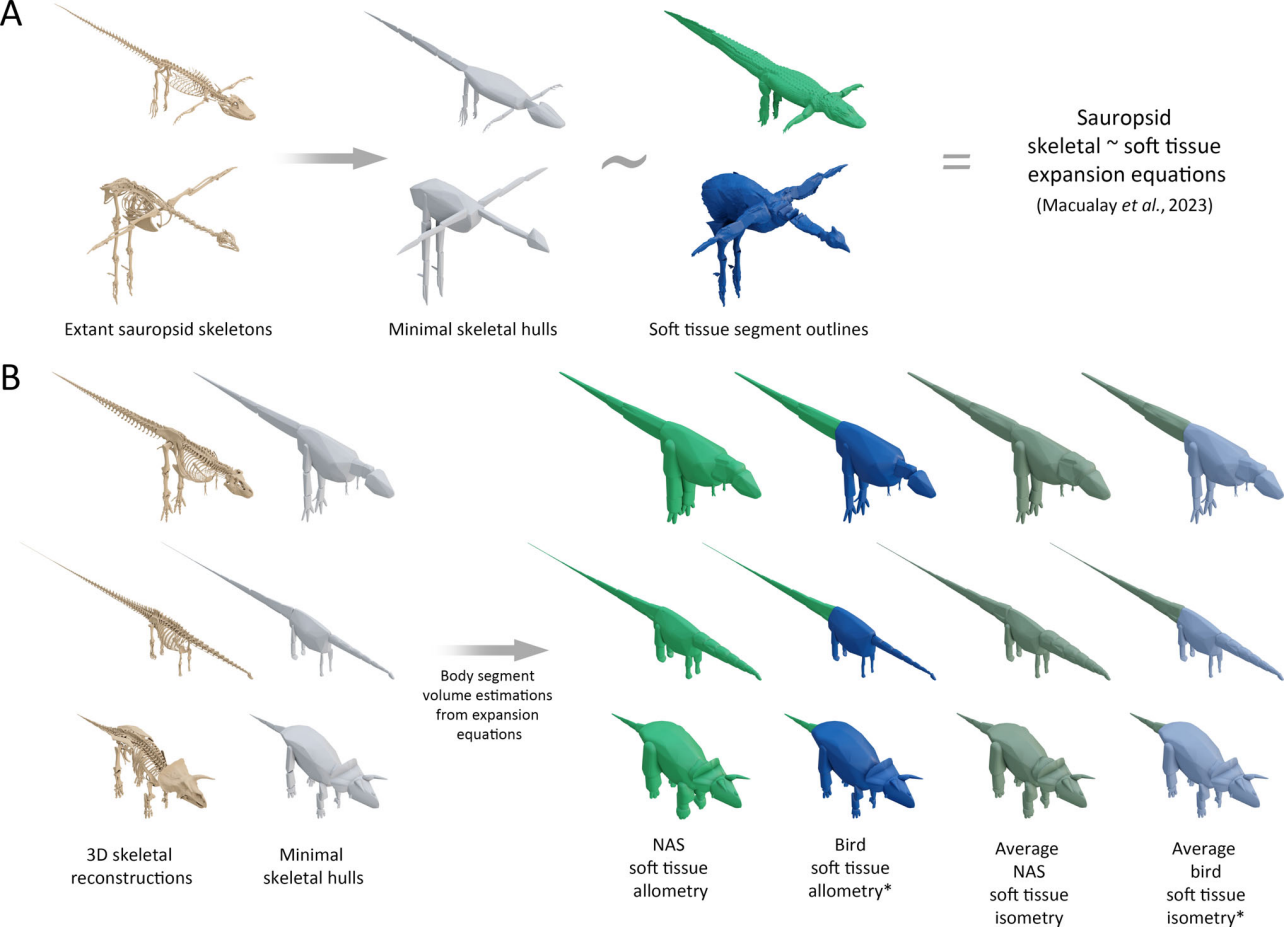
(A) Graphical representation of the convex hull expansion workflow used to estimate body mass in this study from the Macaulay *et al*. (2023) extant sauropsid skeletal hull ~ soft tissue expansion factors, illustrated using *Alligator* and *Numida* as examples. (B) Illustrations representing the expanded dinosaur body segment volumes using *Tyrannosaurus*, *Diplodocus*, and *Triceratops* as examples. *The reduced tails of birds are contained within the torso hulls in Macaulay *et al*. (2023); bird‐based dinosaur models therefore used the non‐avian sauropsid (NAS) tail expansions.

Two recent studies, Coatham *et al*. (2021) and Macaulay *et al*. (2023), addressed this uncertainty by further refining the convex hull workflow by using computed tomography (CT) data from extant tetrapods (mammals in the former, sauropsids in the latter), to generate predictive relationships between the dimensions of the minimal convex hulls of specific skeletal segments (e.g. torso, neck, head, etc.) and their corresponding soft tissue envelopes (Fig. 2A). To address partially the potential issues with the extrapolation of allometric relationships to dinosaurs far beyond the size range of extant sauropsids, Macaulay *et al*. (2023) also calculated an alternative set of average isometric expansion factors between the convex hull and soft tissue volumes for each body segment of the extant sauropsid taxa. The scaling factors of Macaulay *et al*. (2023) were applied to a set of convex‐hulled skeletons of theropod dinosaurs in order to track body mass and body shape evolution along the evolutionary continuum from non‐avian theropods to crown‐group birds. By defining scaling equations from both birds and non‐avian sauropsids independently, the Macaulay *et al*. (2023) approach also allowed uncertainty inherent to soft tissue reconstruction approaches based on the extant phylogenetic bracket (EPB) (Witmer, 1995) of dinosaurs to be quantitatively assessed. This refined convex hull expansion approach currently represents the most quantitatively data‐driven, anatomically grounded method of reconstructing the volumetric dimensions of fossil tetrapod body segments to date.

## III. STUDY AIMS

The primary goal of this study is to estimate body mass properties in a representative array of non‐avian dinosaurs that are empirically grounded in extant archosaur soft tissue data. This was carried out by applying the Macaulay *et al*. (2023) workflow to a data set of 53 skeletal models covering the majority of major dinosaur clades, body plans, and body sizes, including 16 theropods, 19 sauropodomorphs, 17 ornithischians, and one non‐dinosaurian dinosauriform (*Marasuchus*). Given that the Macaulay *et al*. (2023) expansion workflow facilitates a more modular approach to the estimation of body masses by quantifying the dimensions of each body segment independently, we were able to use our models as a starting point to examine functional variation in dinosaur body segment proportions from a basis more empirically grounded in extant anatomical data than previous studies. We approached this from two perspectives: tracking broad evolutionary patterns in relative whole‐body centre of mass across Dinosauria as a means of summarising overall changes in whole‐body proportions, and examining how the relative dimensions of specific body segments varied across different body sizes and between major dinosaur clades.

## IV. METHODS

### (1) Model construction

Body segment convex hull volumes for each taxon were generated from 3D skeletal models. These were derived either from 3D scan data and accurately sculpted models newly prepared for this study, or from previously published scan data and models that had already been hulled for prior studies on body mass properties. Skeleton models were selected primarily on the basis of specimen/taxon completeness and/or proportional accuracy, and a complete summary of each is provided as online Supporting Information in Table S1. New models were prepared in Blender version 3.1 (Blender Foundation, 2022) by dividing the body into functional segments, and generating minimal convex hulls from those segments following reorientation into a standardised reference pose in which the axial skeleton was anteroposteriorly extended, and the limb segments were pointed ventrally (Fig. 2B). This pose does not represent an *in‐vivo* posture, but instead represents a necessarily standardised point of reference from which anatomical measurements can be taken and compared, following the approach of previous studies (e.g. Allen *et al*., 2009, 2013; Hutchinson *et al*., 2011; Bates *et al*., 2016; Macaulay *et al*., 2017, 2023). The approximate position of the shoulder girdle is somewhat more subjective than other elements of the reference pose due to the lack of skeletal joints between the shoulder girdle and torso. Where necessary, the shoulder girdles of the skeletal models were edited based on fossil skeletons of the same or related taxa preserved in articulation, as well as previous hypotheses on dinosaur scapular orientation and glenoid position (e.g. Schwarz, Frey & Meyer, 2007a; Senter & Robins, 2015). The exact orientation of the shoulder girdle is sensitive to ribcage morphology, but in general, the coracoids are placed anterior to the distal ends of the anterior thoracic ribs, with birds and their closest relatives having somewhat shallower scapular inclinations, and therefore a more dorsal glenoid. Where necessary, head, neck, tail, manus, and pes segments were further divided into additional segments so that the convex hulls more closely conformed to overall skeletal forms.

We then applied the extant sauropsid convex hull scaling equations of Macaulay *et al*. (2023) to the measured convex hull volumes in order to generate four alternative sets of body volume estimations for each dinosaur – one derived from the non‐avian sauropsid allometric equations, one derived from the bird allometric equations, one derived from the average non‐avian sauropsid isometric scaling factors, and one derived from the average bird isometric scaling factors (Fig. 2B). In some scenarios, the bird‐based scaling factors of Macaulay *et al*. (2023) may produce estimated body segment volumes lower than the minimum convex hulls. This is either the result of extrapolated negative allometry, or non‐convex shapes in the real soft tissue outlines that cause them to become less voluminous than the hulls. With the latter reason in mind, negative scaling of the convex hull volumes to estimate soft tissue volumes is not necessarily biologically infeasible. For example, convex hulls formed around skulls typically contain a considerable amount of empty space that can hypothetically be collapsed and redistributed without the skull becoming ‘shrink‐wrapped’. However, in body segments for which the dinosaur convex hulls adhered more tightly to overall forms, such as the neck and metatarsal segments, the convex hull volumes were treated as a minimum threshold for estimated segment volumes (see also Section VI.1, and Appendix S1) No expansion factors were applied to ornamental structures and major osteoderms in taxa where these were present, around which the dermal envelopes were assumed to have wrapped more tightly than ‘fleshier’ body segments. Uncertainty in the allometric estimates was evaluated using the mean absolute percentage prediction error (mPPE) of each body segment equation, chosen here as the preferred error metric because it deals with the overall predictive strength of scaling relationships in relation to the untransformed source data (Campione & Evans, 2012, 2020).

Body densities in each of the primary model sets were defined heterogeneously following the approach of Macaulay *et al*. (2023), in which the head, tail, and limb segments were set at 1000 kg/m^3^, and the neck and torso segments were set at 800 and 850 kg/m^3^, respectively, to account for the presence of respiratory structures. Thyreophoran osteoderms and ceratopsian cranial ornamentations were set at a density of 2000 kg/m^3^, following the approach of previous studies when restoring regions assumed to be primarily made up of more compact bone (e.g. Maidment *et al*., 2014b; Mallon *et al*., 2018). Whole‐body centres of mass were calculated by multiplying the Cartesian coordinates of each segment's centre of mass by its mass, and dividing the sum of these by total body mass. The Cartesian coordinates of each segment's centre of mass were defined based on their displacement from the acetabula. We herein focus on anteroposterior and dorsoventral centres of mass, because even in cases of slight model asymmetry (either as an artifact of imperfect skeletal reconstruction or true biological asymmetry, such as the overlapping plates of *Stegosaurus*), whole‐body mediolateral centre of mass is either in the sagittal plane or only negligibly displaced from it.

### (2) ‘Preferred’ models

While the Macaulay *et al*. (2023) expansion workflow maximises objectivity in the determination of body segment volumes, it does not entirely remove subjectivity. The varied skeletal morphologies of dinosaurs suggest that a single group of expansion factors cannot be uniformly applied to a single taxon, as some skeletal forms may bear closer resemblance to extant non‐avian sauropsids, whereas others may bear closer resemblance to extant birds. In addition to the four base variant model sets, we therefore produced two sets of more subjective ‘preferred’ volume reconstructions in which the most applicable scaling factors for each body segment were assessed on a taxon‐by‐taxon basis. Complete details of how each of the scaling factors were applied to generate the preferred model sets are outlined in Appendix S1, with sources of subjectivity further outlined in Section VI.1. One of these preferred model sets was derived primarily from the allometric convex hull scaling equations, whereas the other was derived primarily from the average isometric convex hull expansions. To assess whether relative differences in total body mass were retained between each of the model sets derived from the different Macaulay *et al*. (2023) expansions, we carried out Spearman's rank order analyses in R version 4.2.2 (R Core Team, 2022).

### (3) Sensitivity analyses

To test the effects of our heterogenous density assumptions on both reconstructed masses and reconstructed centres of mass, each of our models was given a set of alternative homogenous density values based on maximal and minimal overall densities in extant taxa derived from previous literature. Maximal (1169 kg/m^3^) and minimal (731 kg/m^3^) avian density values were derived from the values collated by Brassey & Sellers (2014, and references therein). We only used densities derived from plucked birds, as the soft tissue volumes used to define the Macaulay *et al*. (2023) expansion factors did not include feather volumes.

The cervical vertebrae of many sauropods are inferred to have possessed extensive pneumatic diverticula (e.g. Schwarz, Frey & Meyer, 2007b). Following the protocol of Macaulay *et al*. (2023), our main heterogenous density models treat the neck as the least dense body segment (800 kg/m^3^), which is consistent with results derived from manual reconstructions of cervical air spaces based on osteological correlates (e.g. Larramendi *et al*., 2021). However, the extent of these diverticula is subjective, and it therefore cannot be ruled out that their presence may have caused sauropod necks to be considerably less dense than those of other dinosaurs. To test the effects of lower hypothetical neck densities on sauropod mass property estimates, an alternative set of heterogenous density sauropod model values was generated with low neck densities of 500 kg/m^3^, following a similar sensitivity analysis carried out by Bates *et al*. (2016).

Many dinosaurs have hyperelongate neural spines, which in some taxa are hypothesised to have supported minimal soft tissues, forming a sail like structure similar to that of some extant lizards (Ibrahim *et al*., 2020; Cerda *et al*., 2022) and in other taxa have a hypothesised association with expanded epaxial muscles based on specific osteological correlates for the erector spinae and transversospinalis muscle groups (Snively & Russell, 2007; Organ, 2006), with a fatty hump‐like structure also sometimes proposed (Bailey, 1997). While histological evidence for a sail‐like structure has been identified in *Amargasaurus* (Cerda *et al*., 2022), the reconstruction of many other taxa remains equivocal. To test the possible effects of varying neural spine soft tissue hypotheses on body mass estimates, we took *Acrocanthosaurus* (the taxon in our data set for which neural spine elongation was the most extensive across the entire axial skeleton), and produced a set of alternative models. The primary models for *Acrocanthosaurus* were derived from expanded convex hulls enveloping the elongate neural spines alongside the corresponding axial segments, producing ‘hump‐backed’ axial volumes with a large convex space between the ribcage and/or transverse processes and neural spine tips (Fig. S1A). In the alternative models, the neural spines were hulled separately, creating minimal ‘sail‐backed’ axial volumes (Fig. S1B). The separately hulled sails were not expanded alongside the rest of the axial hulls, and were given a density of 1000 kg/m^3^, as was done for *Amargasaurus* in the primary model set. Due to the relative robustness of the neural spines of *Acrocanthosaurus*, a second set of ‘sail‐backed’ models with a more compact sail density of 2000 kg/m^3^ was also generated.

The presence of elaborate osteoderms poses a unique challenge when attempting to reconstruct the body volumes of thyreophorans (armoured ornithischian dinosaurs). Given that they are derived from the soft tissue volume, the mass of ossified dermal structures would in theory be accounted for by the expansion of just the primary skeletal hulls. However, the major osteoderms of thyreophorans are often much larger than those of extant non‐avian sauropsids, and would have protruded far beyond the ‘normal’ soft tissue envelope. To assess the effects that separately considering major osteoderms would have on body mass properties, total body masses and whole‐body centres of mass were recalculated without the osteoderm hulls (Fig. S2).

Ceratopsian cranial ornamentation was initially assumed to be composed of more compact bone and less extensive fleshy tissues than the rest of the head. However, horns have a hollow core, and ceratopsian frills are often either fenestrated or exhibit regions of more thinly constructed bone (e.g. Scanella & Horner, 2010), particularly in chasmosaurines, in which much of the frill resembles a thin frame around very broad parietal fenestrae (e.g. Godfrey & Holmes, 1995). The horns and frills of ceratopsians may therefore have been less dense than in our primary models, which used a compact density of 2000 kg/m^3^ for ornamental structures. To test the effect of the initially assumed compact density of the ornamental structures (Fig. S3), we produced an alternative set of ceratopsian head models in which the ornamentation density was lowered to 1000 kg/m^3^, matching the density of the main head segments.

The long necks of sauropods make up a much greater proportion of their total body mass compared to other dinosaurs, meaning that changes in neck posture from the reference pose might result in greater centre of mass changes than in other taxa. Furthermore, the habitual posture of the sauropod neck has been highly debated (e.g. Stevens & Parrish, 1999; Taylor, Wedel & Naish, 2009; Christian, 2010; Stevens, 2013; Vidal *et al*., 2020). To quantify the effect of neck posture on our reconstructed centres of mass, we produced alternative models of a representative selection of sauropods (*Shunosaurus*, *Omeisaurus*, *Apatosaurus*, *Barosaurus*, *Giraffatitan*, and *Rapetosaurus*) in which the necks were pitched 45° above the horizontal orientation of the base models (Fig. S4).

### (4) Comparisons of body masses with previous methods

In order to place our models within the context of previous approaches, we compiled previous volumetric body mass estimates of taxa in our sample based on skeletons of equivalent size from the literature, and also applied the 21% expansion convex hulling protocol of Sellers *et al*. (2012) and subsequent works (e.g. Bates *et al*., 2016) to the skeletal models prepared for this study. In order to compare our model masses with mass estimates derived from stylopodial equations, we compiled humeral and femoral circumference data for as many of our modelled taxa as possible (see Table S2 for a full list of data sources), and used the linear bipedal and quadrupedal equations of the MASSTIMATE package version 2.0‐1 (Campione, 2020) in R version 4.2.2 (R Core Team, 2022) to derive alternative mass estimates. We used Spearman's rank analyses in R version 4.2.2 (R Core Team, 2022) to compare ranked similarities between the model mass estimates derived from the different Macaulay *et al*. (2023) expansions, previous volumetric approaches, and MASSTIMATE equation estimates.

### (5) Analysis of dinosaur body proportions

Analysis of relative dinosaur body proportions was carried out from two perspectives – the estimation and comparison of whole‐body centres of mass, and the examination of the estimated dimensions of each body segment relative to total body mass. To compare non‐dimensionalised (size‐relative) centres of mass, we used two different normalisation metrics – glenoacetabular distance (both anteroposterior and dorsoventral, measured relative to the reference pose), and total body mass to the power of one‐third. The former provides a direct summation of the centre of mass relative to the proximal joints of each limb, which is important for interpreting relationships between mass distribution and locomotion, whereas the latter provides a holistic summary of centre of mass relative to the 3D form of the entire body. To investigate which body segments were most associated with shifts in centre of mass, segment masses were expressed as a percentage of total body mass and regressed against the non‐dimensionalised centres of mass using ordinary least squares (OLS), as well as phylogenetic generalised least squares (PGLS) *via* the nlme package version 3.1 (Pinheiro, Bates & R Core Team, 2022) in R version 4.2.2 (R Core Team, 2022), assuming a Brownian model of evolution, with model fits compared based on the sample‐adjusted coefficients of determination (*R* ^2^), and sample corrected Akaike Information Criteria (AICc). To determine potential allometric relationships between total body size and centre of mass, log_10_‐transformed centres of mass were also regressed against log_10_‐transformed total body mass using both OLS and PGLS. Theropods, sauropodomorphs, and ornithischians were each considered independently. To track centre of mass evolution across Dinosauria, we produced phylomorphospace scatter plots for each model set using the *phylomorphospace* function of Phytools version 2.0‐4 (Revell, 2012, 2023) in R version 4.2.2 (R Core Team, 2022), which uses the *FastAnc* function to estimate ancestral states at internal nodes. To compare the ranked similarity of centre of mass estimates between model sets, we again used Spearman's rank analyses in R version 4.2.2 (R Core Team, 2022).

To examine the relative proportions of individual body segments across Dinosauria, and how these proportions varied alongside changes in total body mass, log_10_‐transformed segment masses and lengths were regressed against log_10_‐transformed total body masses using OLS and PGLS. Segment masses as a percentage of body mass and non‐dimensionalised segment lengths (segment lengths divided by body mass to the power of one‐third) were also examined and compared independently. For our centre of mass phylomorphospaces and PGLS regressions, we constructed a supertree of the 52 dinosaur taxa plus the non‐dinosaurian dinosauriform *Marasuchus* (Fig. S5, Appendix S2) in R version. 4.2.2 (R Core Team, 2022). The overall topology was based on recent literature (Table S3), and time scaling was performed with the *timePaleoPhy* function of Paleotree v. 3.4.5 (Bapst & Wagner, 2022) in R version 4.2.2 (R Core Team, 2022), using the ‘equal’ time‐scaling method with the ‘vartime’ set to 1 Ma, and the ‘firstlast’ date treatment setting. Taxon ages and internal node ages were calibrated using occurrence ranges based on the literature (Table S3).

## V. RESULTS

### (1) Body mass estimates

**Fig. 3:**
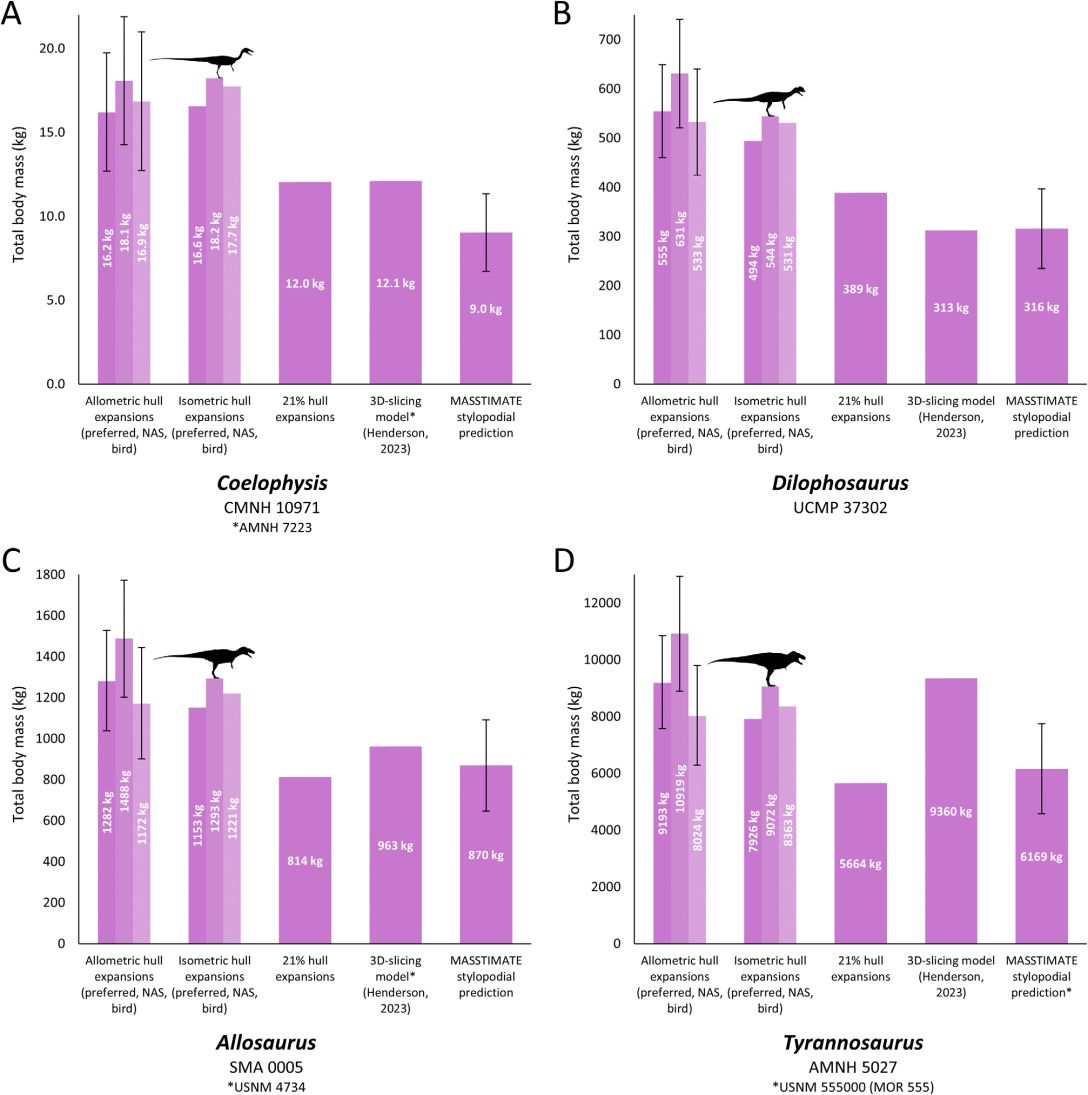
Comparisons of our mass estimations (allometric hull expansions and isometric hull expansions) of selected theropods with previous methods and estimates: (A) *Coelophysis*, (B) *Dilophosaurus*, (C) *Allosaurus*, (D) *Tyrannosaurus*. NAS = non‐avian sauropsid. Error bars on the allometric hull expansions are calculated from the mean absolute percentage prediction error (mPPE) of each body segment, and error bars on the stylopodial predictions are derived from the MASSTIMATE mPPE.

**Fig. 4:**
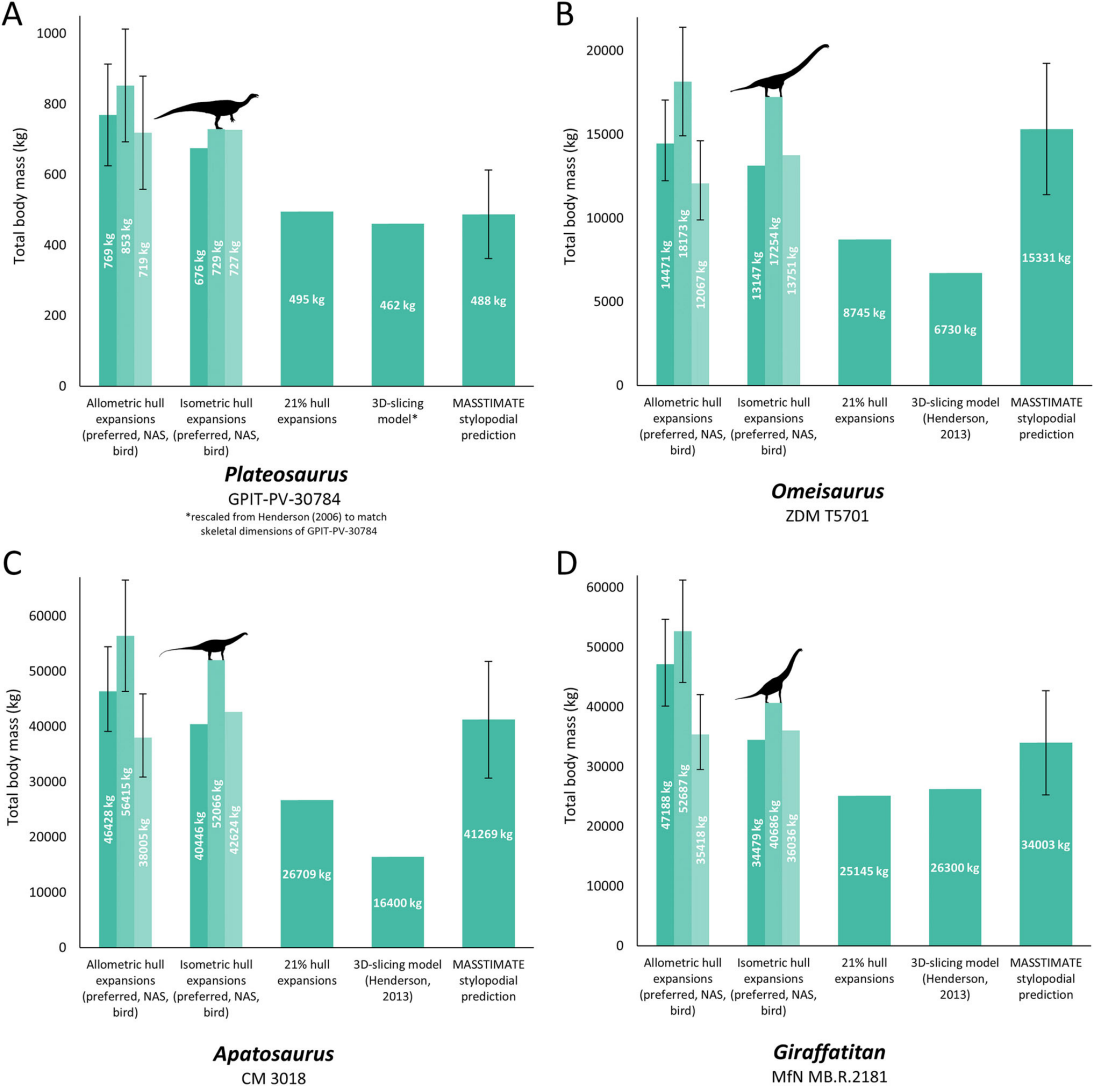
Comparisons of our mass estimations (allometric hull expansions and isometric hull expansions) of selected sauropodomorphs with previous methods and estimates: (A) *Plateosaurus*, (B) *Omeisaurus*, (C) *Apatosaurus*, (D) *Giraffatitan*. NAS = non‐avian sauropsid. Error bars on the allometric hull expansions are calculated from the mean absolute percentage prediction error (mPPE) of each body segment, and error bars on the stylopodial predictions are derived from the MASSTIMATE mPPE.

**Fig. 5:**
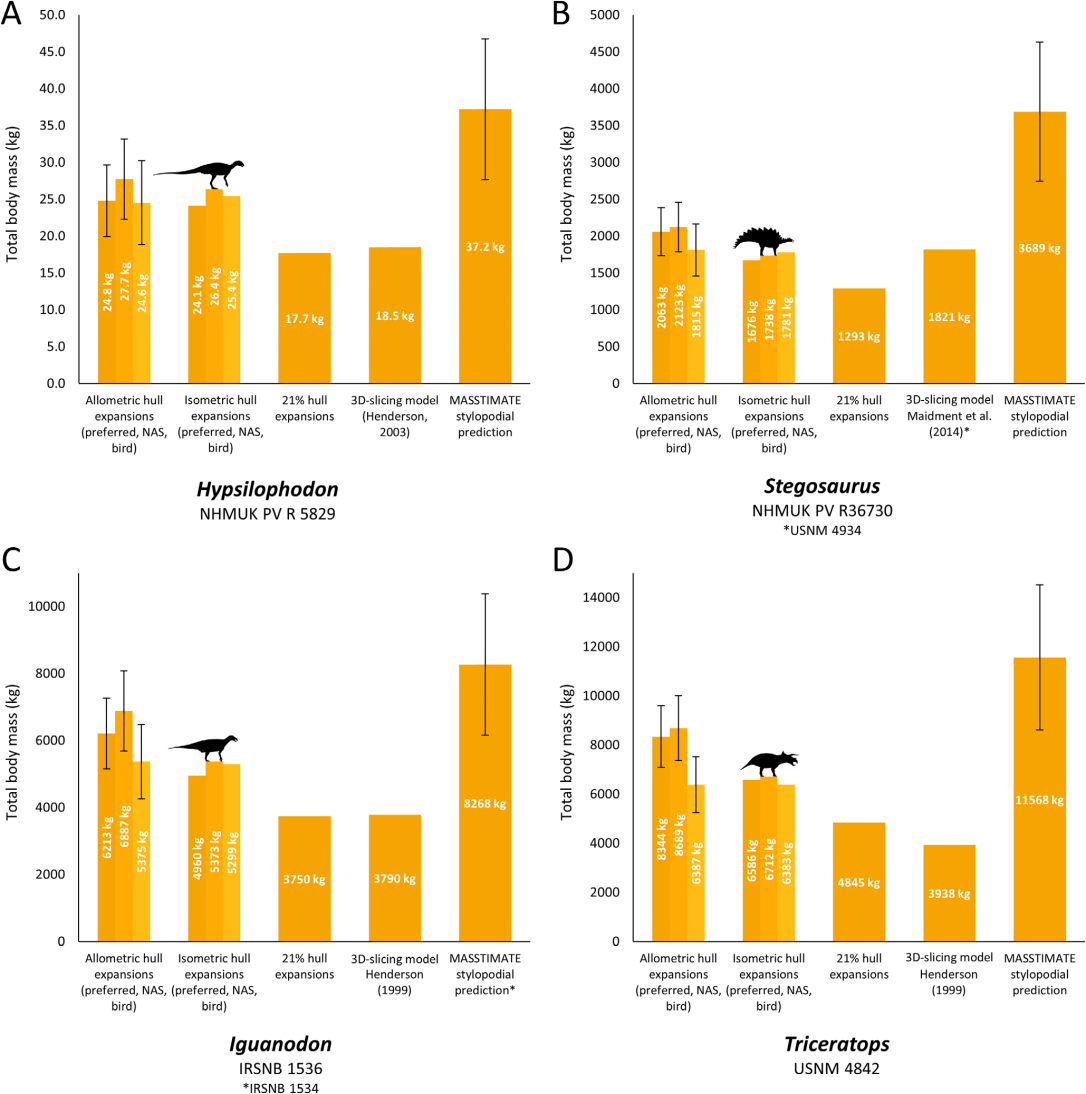
Comparisons of our mass estimations (allometric hull expansions and isometric hull expansions) of selected ornithischians with previous methods and estimates: (A) *Hypsilophodon*, (B) *Stegosaurus*, (C) *Iguanodon*, (D) *Triceratops*. NAS = non‐avian sauropsid. Error bars on the allometric hull expansions are calculated from the mean absolute percentage prediction error (mPPE) of each body segment, and error bars on the stylopodial predictions are derived from the MASSTIMATE mPPE.

Heterogenous density body mass ranges for each model and taxon are outlined in Table S4, and complete individual segment masses are provided in Table S2. The lightest taxon in the study was the avialan theropod *Yixianornis*, in which model mass estimates were found to be lower than 200 g. The heaviest taxon in the study was the titanosaurian sauropod *Patagotitan*, for which most model mass estimates were greater than 60 tonnes. With the exception of the torso, reconstructed body segment masses (and therefore total body masses) were mostly higher when using the non‐avian sauropsid‐based expansions than the bird‐based expansions (Figs 3, 4, 5, Table S4). The total body masses of the subjectively preferred isometric models were either lower than or at the lower end of the standard non‐avian sauropsid‐based and bird‐based isometric models, as most used the less voluminous non‐avian sauropsid‐based torso hulls and bird‐based neck hulls (see Section (1) and Appendix S1), leading to a decrease in mass that was not offset by the relatively heavy non‐avian sauropsid‐based limb hulls. The masses of the preferred allometric models were constrained within the range of the standardised non‐avian sauropsid‐based and bird‐based allometric models. Overall, the range of body masses estimated from the allometric expansions overlapped with the isometric expansions (Figs 3, 4, 5). Non‐avian sauropsid allometric expansions tended to produce higher body masses than the corresponding isometric expansions in the largest multi‐tonne dinosaurs, whereas differences in total body mass between the bird‐based allometric and isometric expansions were minor (Figs 3, 4, 5, Table S4). Ranked differences in body mass between taxa were closely mirrored between each of the variant model sets (Spearman's rho >0.99, Table S2), only differing slightly differing as the result of minor variations between taxa of very similar body size.

Given that each body segment was scaled according to a different equation, and that a handful of body segments in some models used the minimal hull volumes when negative expansions were deemed to be implausible, the magnitude of uncertainty in the allometric model masses varied according to proportional differences between taxa. Tail volume, for which mPPE was ±52%, represents the largest source of uncertainty in the estimates, while the volume of the torso (which is also the proportionally largest segment of the body in all taxa, and thus the most important determinant of total body mass) has a narrow mPPE of ±10% in the non‐avian sauropsid equations, and ± 14% in the bird equations. The overall total body mass error bars calculated from the mPPE of each body segment ranged between 74% and 86% of the point estimates at their lower margin, and between 114% and 126% of the point estimates at their upper margin.

### (2) Sensitivity analyses

The use of the minimum homogenous avian densities caused models to vary between 79% and 86% of the mass of the heterogenous density models, whereas the use of maximum homogenous avian densities caused models to vary between 125% and 137% of the mass of the heterogenous density models (Table S5). The use of homogenous segment densities mostly had a relatively minor effect on the relative anteroposterior centres of mass, with shifts of up to 5% of the glenoacetabular distance from the heterogenous density models in the majority of taxa. Somewhat more pronounced shifts of up to 11% of the glenoacetabular distance occurred in front‐heavy taxa with particularly large necks (e.g. *Barosaurus*) or heads (e.g. *Chasmosaurus*) (Table S5). In most taxa, shifts in the dorsoventral centre of mass between heterogenous and homogenous density models were less than 10% of the dorsoventral glenoacetabular distances, although this relative shift was considerably more pronounced in taxa with proportionally very narrow dorsoventral spacing between the glenoid and acetabulum, or with centres of mass ventral to the glenoid (e.g. *Staurikosaurus*, *Archaeopteryx*) (Table S5).

In the alternative heterogenous density sauropod models with low neck densities (500 kg/m^3^), changes in body mass estimates from the original models were mostly relatively minor. For example, lowering neck density in sauropod model estimates that used the bird‐based isometric neck volumes only led to a 2–9% reduction in total body mass (Table S5). Considerably larger drops in body mass of between 4% and 16% were seen when using the non‐avian sauropsid isometric expansions, but note that the extremely large neck volumes of these model variants were subjectively judged to be unrealistic for sauropods compared to the bird‐based expansions (see Section VI.1). The greatest drop in total body mass following neck density reduction was seen in the mamenchisaurid *Omeisaurus* (e.g. −9% in the preferred isometric models), which has the proportionally most voluminous neck of any dinosaur in the data set. As found in a similar sensitivity analysis by Bates *et al*. (2016), changes in relative centre of mass were variable. For example, when using the preferred isometric expansions, the centre of mass of most sauropods shifted posteriorly by less than 10% of glenoacetabular distance following the reduction of neck density. Greater posterior shifts were recovered in very long‐necked taxa (e.g. a posterior shift of 18% glenoacetabular distance in *Omeisaurus*). It is important to note, however, that sauropods with extreme neck proportions are still recovered as very front‐heavy animals even following such a large reduction in hypothetical neck density. For example, when using the preferred isometric expansions and a neck density of 500 kg/m^3^, *Omeisaurus* is still recovered with a highly anterior centre of mass of 78% glenoacetabular distance.

In the analysis of varying neural spine soft tissue reconstructions on *Acrocanthosaurus*, the ‘sail‐backed’ model variants with lower density sails (1000 kg/m^3^) ranged from 85% to 89% of the mass of the ‘hump‐backed’ models from the primary data set, while the ‘sail‐backed’ model variants with higher density sails (2000 kg/m^3^) ranged from 85% to 93% of the ‘hump‐backed’ models. The ‘sail‐backed’ models each had a somewhat more anteroventral centre of mass than the primary models (Fig. S1C, Table S5), shifting anteriorly by up to 7% of anteroposterior glenoacetabular distance, and shifting ventrally by up to 13% of dorsoventral glenoacetabular distance. Despite these shifts, the ‘sail‐backed’ *Acrocanthosaurus* models in each model set mostly still retained a more posterior centre of mass than other large theropods. The ‘sail‐backed’ variants of the lower mPPE allometric model estimates were an exception, as they were found to have an anteroposterior centre of mass between 33% and 38% of glenoacetabular distance, and were thus closely comparable to the tyrannosaurids from the corresponding model sets.

The exclusion of separately hulled major osteoderms had only a minor effect on total body mass in the thyreophorans, reducing mass by between 3% and 8% relative to the models in which they were included (Table S5). Major osteoderm exclusion resulted in centre of mass shifts of up to 5% anteroposterior glenoacetabular distance and up to 7% dorsoventral glenoacetabular distance relative to the models in which they were included (Fig. S2, Table S5). This corroborates previous studies which also found that thyreophoran major osteoderms only made up a small percentage of total body mass, and thus would only lead to small differences in centre of mass if excluded or rearranged (Henderson, 1999; Maidment *et al*., 2014b; Mallison, 2014).

Reducing the density of ceratopsian cranial ornamentation only had a minor effect on total body mass in ceratopsians, changing body mass by less than 6% (Table S5), and corroborating previous studies in which ceratopsian cranial ornamentation was found to make up only a small proportion of total body mass (Maidment *et al*., 2014b). Changes to whole‐body centres of mass were also relatively minor, mirroring previous models that also varied ceratopsian cranial ornamentation (Maidment *et al*., 2014b), changing by up to 7% of anteroposterior glenoacetabular distance, and up to 4% of dorsoventral glenoacetabular distance (Fig. S3, Table S5). Total head masses decreased to between 68% and 89% of the original head masses. The greatest changes in head mass and whole‐body centre of mass were seen in *Chasmosaurus*, as a result of the large size of the frill. Even with lower ornamentation densities, ceratopsians were still found to be considerably more front‐heavy than other ornithischians.

Pitching the necks dorsally by 45° from horizontal in a selection of the sauropod models resulted in a posterodorsal shift in whole‐body centres of mass relative to the original models, mirroring previous similar analyses (Bates *et al*., 2016) (Fig. S4, Table S5). In most of the model variants, the posterior shift was less than 10% of glenoacetabular distance. However, in the isometric model variants of *Barosaurus*, as well as the non‐avian sauropsid isometric model variant of *Omeisaurus*, this posterior shift was greater, ranging from between 14% and 19% of glenoacetabular distance. Major relative differences in centre of mass between the selected sauropods remained consistent across each variant set following the pitching of the necks. For example, even following the pitching of the neck, *Omeisaurus* was still found to be more front‐heavy than the corresponding model variants of most other sauropods with their necks oriented horizontally. The dorsal shift in whole‐body centres of mass following the pitching of the neck was very variable, and was much more pronounced in the heavier‐necked isometric models than the allometric models (Fig. S4). In the isometric and non‐avian sauropsid models, centre of mass moved considerably dorsal to the acetabulum in *Omeisaurus*, *Apatosaurus*, *Barosaurus*, and *Rapetosaurus*.

### (3) Comparisons of model masses and centres of mass with previous methods

Complete data comparing our models with previous approaches are provided in Table S2, with overall results summarised here. Our models were most easily compared with previous models produced *via* the 3D slicing method, and models produced by expanding minimal body segment hulls by 21% following the method of Sellers *et al*. (2012), due to the abundance of the former in the literature, and the ability to apply the workflow of the latter to the same skeletal models used for this study. When using heterogenous densities, the masses of our volumetric model sets were mostly considerably greater than previous 3D‐slicing models based on the same or equivalently sized skeletons (Figs 3, 4, 5). Of the 31 taxa we were able to compare, only 26% of the 3D‐slicing models overlapped with our heterogenous density model mass ranges, whereas the rest were lighter, ranging from between 25% and 98% the mass of our range of model estimates for any given taxon. Density assumptions in previously published 3D‐slicing model masses vary, with some setting segment‐specific densities in a similar approach to our models (e.g. Maidment *et al*., 2014b), others manually modelling air spaces in considerable detail (e.g. Henderson, 2006), and others opting for a combination of both approaches (e.g. Henderson, 2018). However, in most taxa, our model masses were only found to be closely aligned with 3D‐slicing models (i.e. most 3D‐slicing models were greater than 90% the mass of our least‐voluminous estimates) when minimal homogenous avian densities below those usually assumed for non‐avian dinosaurs were used (see Section VI.2 and Table S5). Despite large quantitative differences, the ranked similarity between the masses of each of our model variant sets and masses produced *via* the 3D‐slicing method was very high (Spearman's rho = ~0.98, Table S2). With the exception of *Archaeopteryx*, *Yixianornis*, and *Protoceratops*, model masses determined by the Macaulay *et al*. (2023) expansions were also greater than models of equivalent segment density generated from the same skeletal reconstructions using the 21% expansion factor approach (Figs 3, 4, 5), but were close to identical in ranked similarity (Spearman's rho = >0.99, Table S2). While fewer models were available for comparison, the range of total body masses produced by the Macaulay *et al*. (2023) scaling factors were found to overlap with the range of masses derived from more subjectively defined minimal and maximal outlines produced *via* the elliptical/octagonal hooping workflow of previous studies (e.g. Bates *et al*., 2009a; Hutchinson *et al*., 2011; Allen *et al*., 2013), but not uniformly so. For example, our 13–22 kg range of body mass estimates for *Coelophysis* lies within the 12–25 kg range of Allen *et al*. (2013), whereas our 425–741 kg range of body mass estimates for *Dilophosaurus* skews heavier than the 298–625 kg range of Allen *et al*. (2013).

**Fig. 6:**
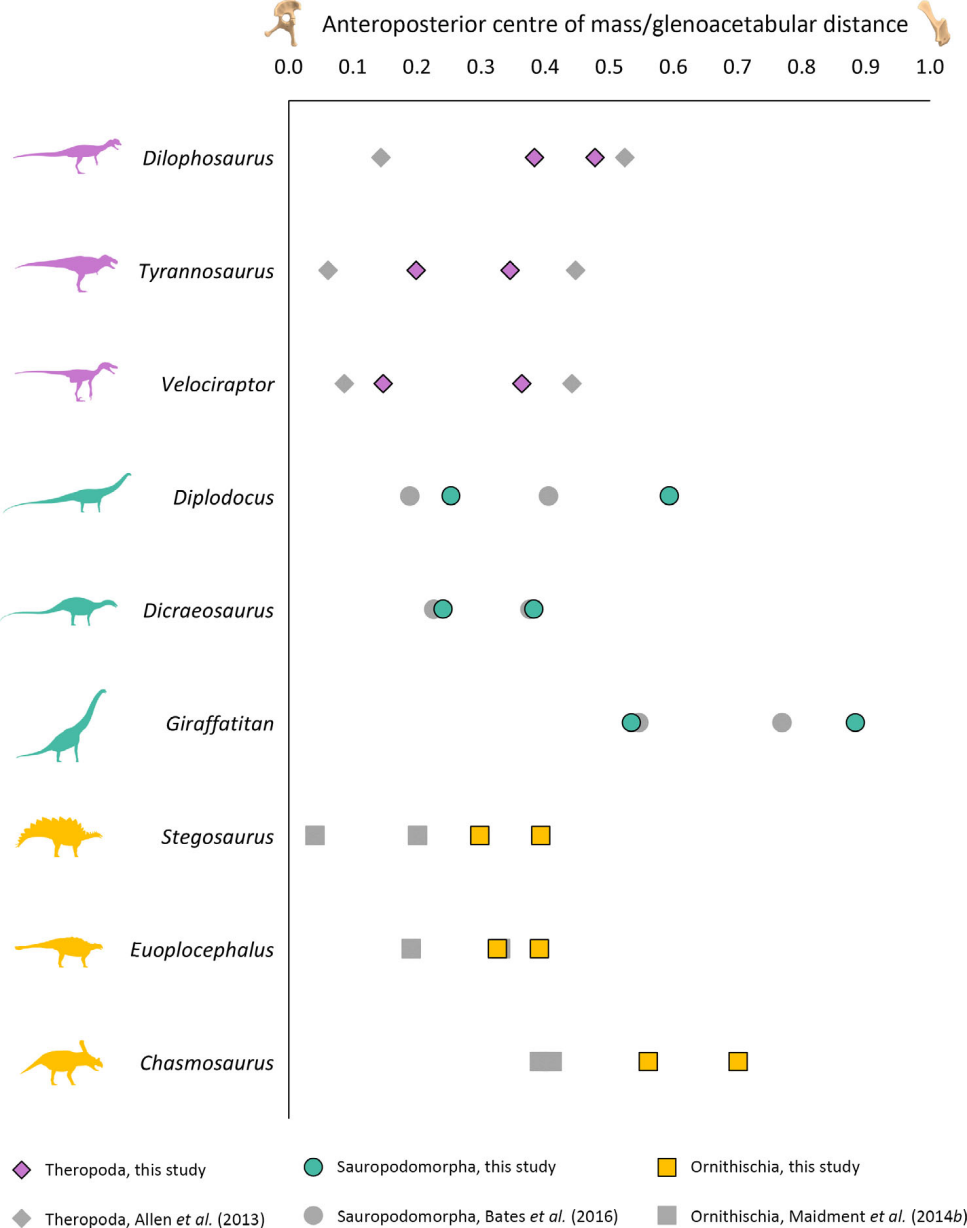
Comparisons of the maximum and minimum anteroposterior centres of mass in a selection of dinosaurs from this study (using the main heterogenous body segment densities as described in the text) with previous studies. On the *x*‐axis, 0 = position of acetabulum, 1 = position of glenoid.

In theropods, our models produced more anteroposteriorly constrained centre of mass ranges than previous volumetric models that subjectively tested variably expanded soft tissue volumes around a single skeletal reconstruction (e.g. Allen *et al*., 2013) (Fig. 6). However, in many sauropods, the range of centres of mass in our models were less constrained than previous models (Bates *et al*., 2016), as well as being more anteriorly skewed as a result of the considerably more massive (though potentially problematic, see Section VI.1) non‐avian sauropsid neck expansions (Fig. 6). Comparing relative constraints on ornithischian centres of mass between workflows is difficult because, to date, only one previous study (Maidment *et al*., 2014b) has performed a sensitivity analysis of alternative volumetric reconstructions that represent the overall disparity of the clade. These previous alternative reconstructions were also fundamentally different from those produced herein, each being built from scratch based on different body outline illustrations using the 3D‐slicing method, as opposed to a series of different volumes expanded around a standardised skeletal reconstruction. In the taxa which could be compared directly between studies, the centre of mass ranges in our *Euoplocephalus* models were close to the most anterior estimate of the Maidment *et al*. (2014b) models, whereas our *Stegosaurus* and *Chasmosaurus* models had considerably more anteriorly skewed centre of mass ranges (Fig. 6). Our hadrosaur centre of mass ranges also overlapped with the Maidment *et al*. (2014b) *Lambeosaurus* models.

Ranked similarity between MASSTIMATE point estimations and the model masses of the 38 taxa in our data set with available stylopodial shaft measurements was very high (Spearman's rho = ~0.96, Table S2). MASSTIMATE predictions were overall quantitatively similar to our model masses, with 70% of the comparable taxa (55% of the theropods, 75% of the sauropodomorphs, and 79% of the ornithischians) having heterogenous density model mass ranges that overlapped with the MASSTIMATE mPPE. However, distribution of the model mass estimates within the MASSTIMATE mPPE varied. In several sauropods, our model mass estimates were reasonably close to the MASSTIMATE point estimates (Fig. 4). Many of our theropod model masses were considerably higher than the MASSTIMATE point estimates (Fig. 3), whereas our ceratopsian and stegosaur model masses were considerably lower than the MASSTIMATE point estimates (Fig. 5). When the full range of homogenous densities from the sensitivity analyses were considered, overlap between model mass ranges and the MASSTIMATE mPPE for each taxon was much higher (95%).

### (4) Centre of mass evolution across Dinosauria

**Fig. 7:**
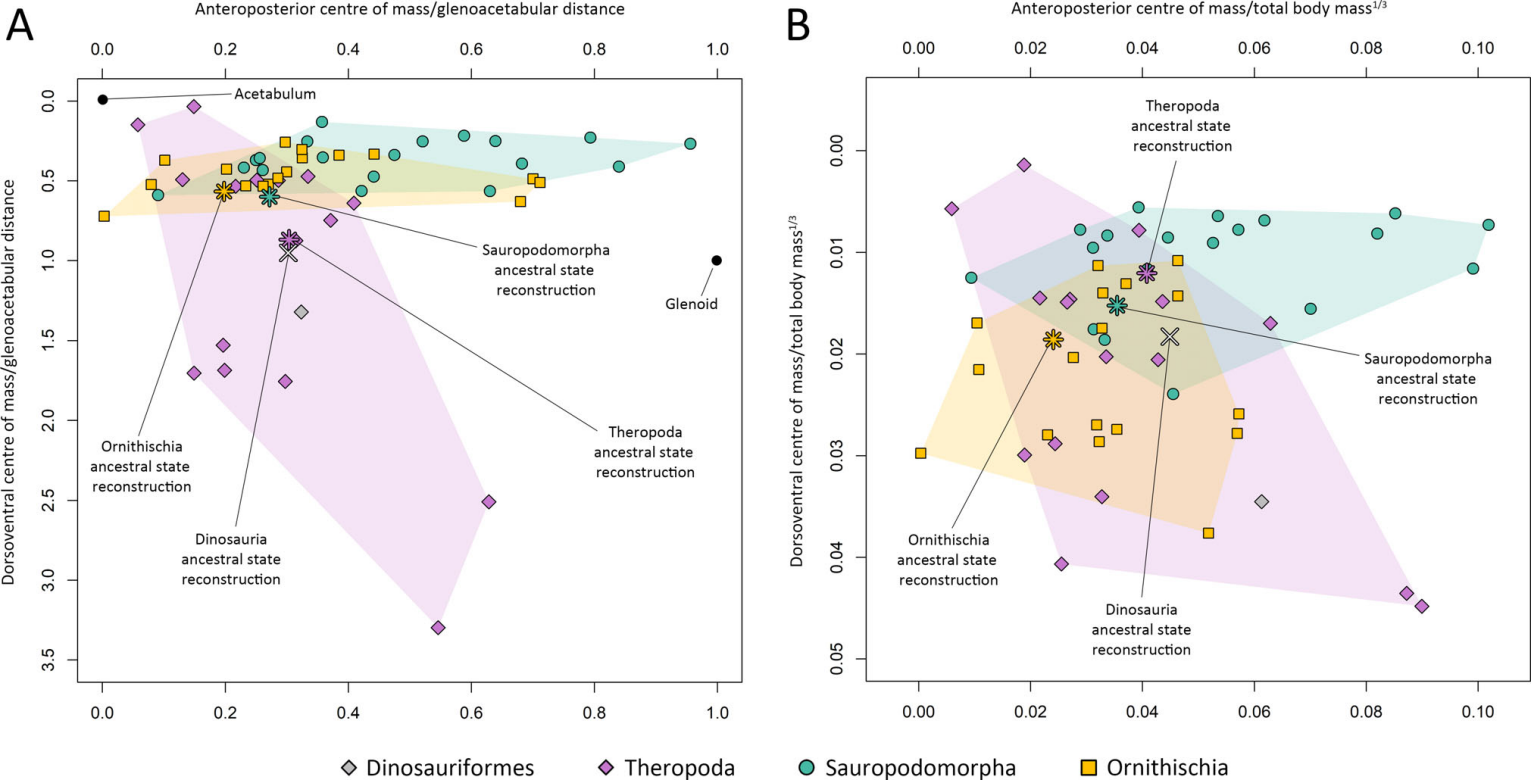
Scatter plots illustrating whole‐body centre of mass disparity and evolution across Dinosauria as a whole, based on the preferred isometric model set. (A) Centre of mass relative to glenoacetabular position. (B) Centre of mass relative to total body mass to the power of one‐third.

**Fig. 8:**
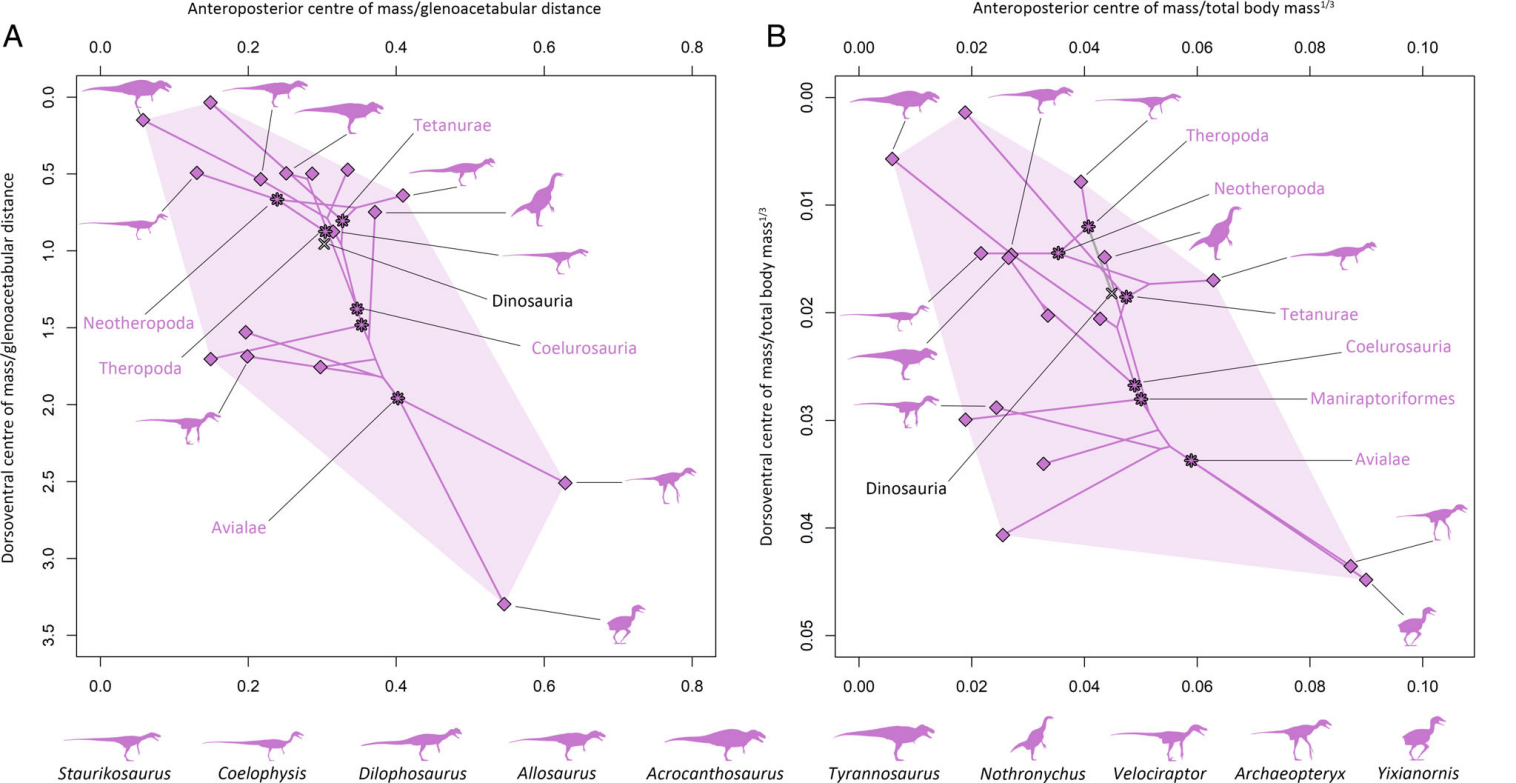
Phylomorphospace scatter plots illustrating whole‐body centre of mass evolution across Theropoda, based on the preferred isometric model set. (A) Centre of mass relative to glenoacetabular position. (B) Centre of mass relative to total body mass to the power of one‐third. Lines interconnecting nodes represent phylogenetic relationships, with internal nodes representing ancestral state reconstructions.

**Fig. 9:**
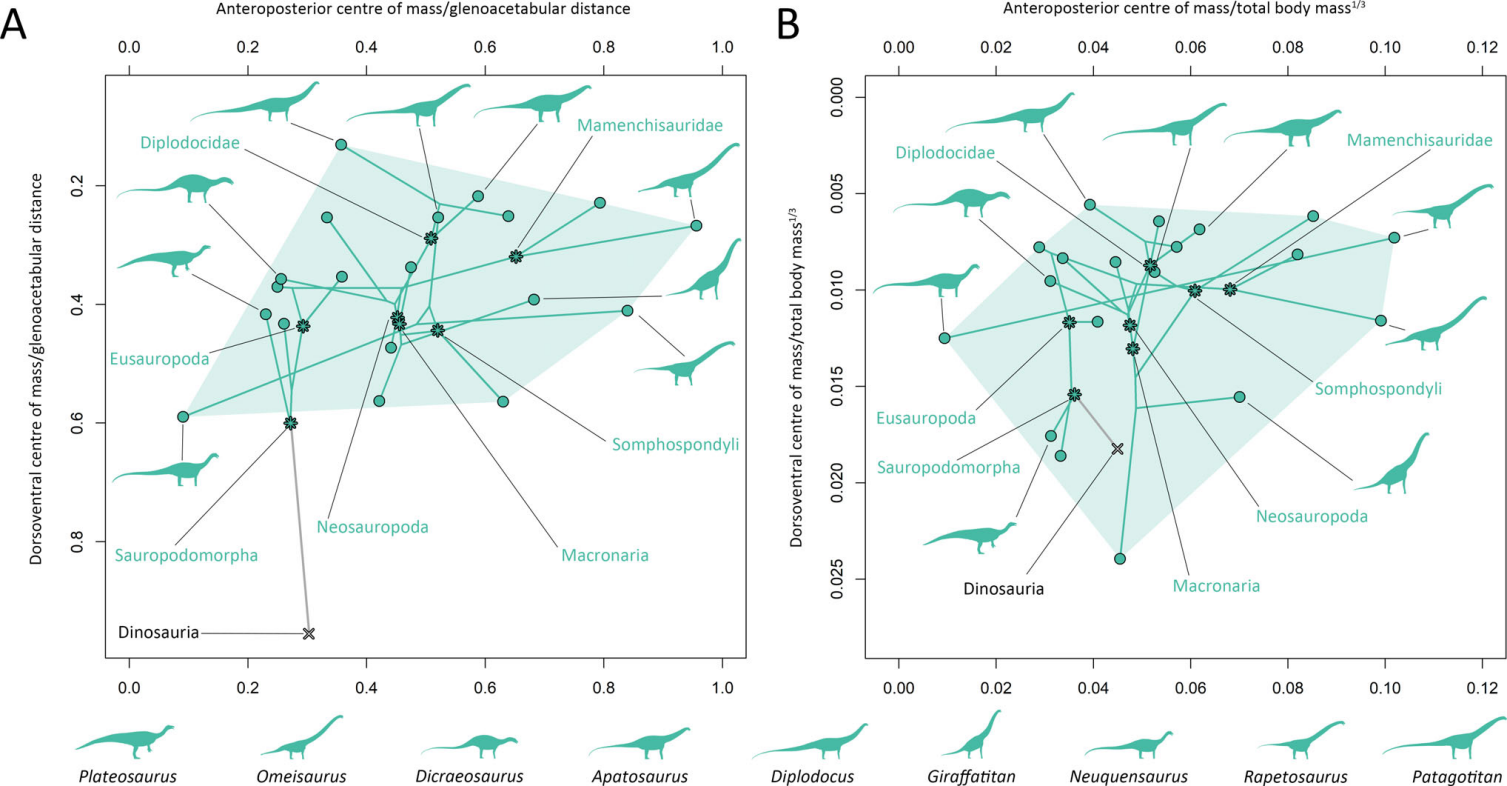
Phylomorphospace scatter plots illustrating whole‐body centre of mass evolution across Sauropodomorpha, based on the preferred isometric model set. (A) Centre of mass relative to glenoacetabular position. (B) Centre of mass relative to total body mass to the power of one‐third. Lines interconnecting nodes represent phylogenetic relationships, with internal nodes representing ancestral state reconstructions.

**Fig. 10:**
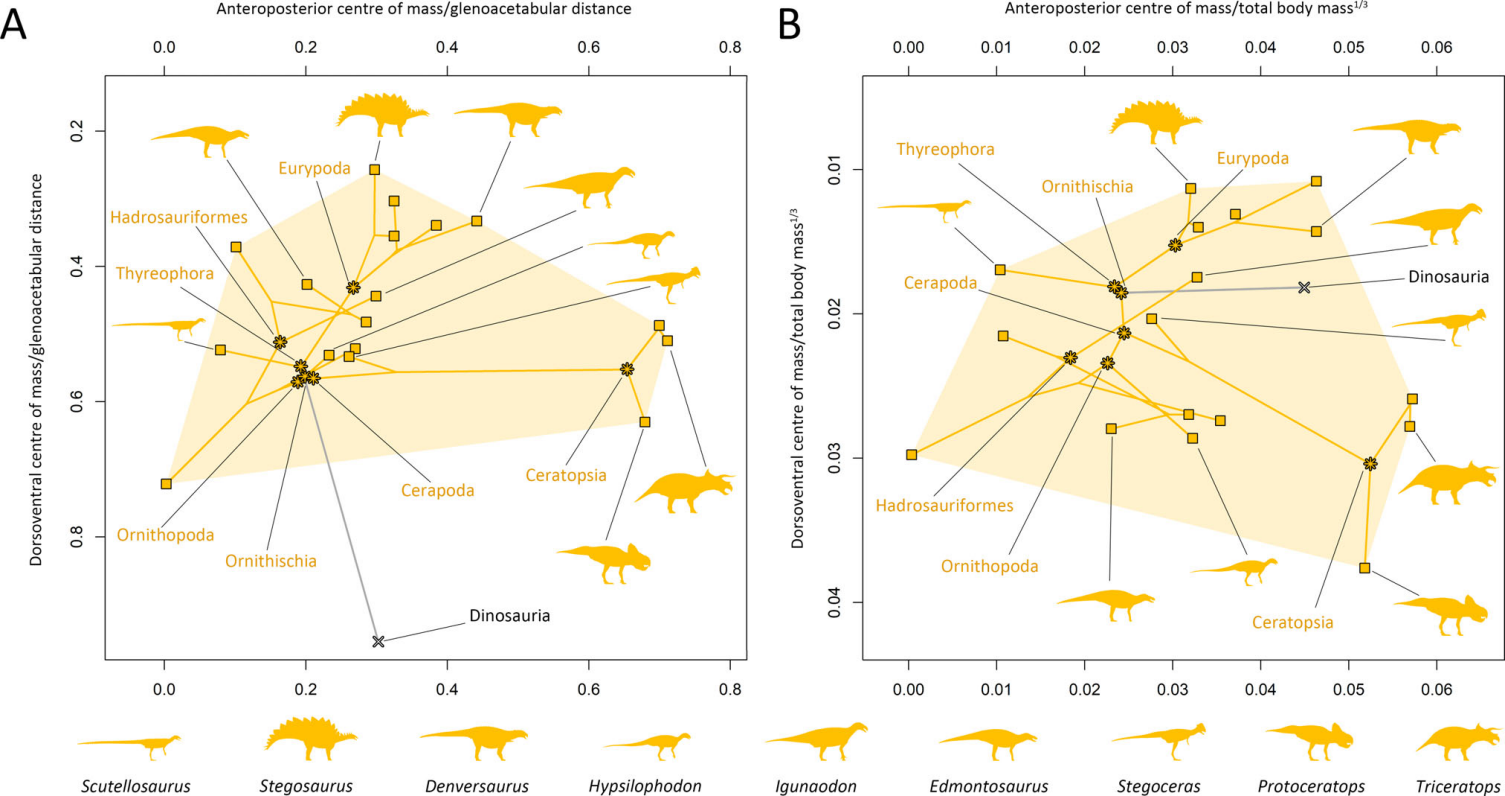
Phylomorphospace scatter plots illustrating whole‐body centre of mass evolution across Ornithischia, based on the preferred isometric model set. (A) Centre of mass relative to glenoacetabular position. (B) Centre of mass relative to total body mass to the power of one‐third. Lines interconnecting nodes represent phylogenetic relationships, with internal nodes representing ancestral state reconstructions.

Our results suggest that the evolution of centre of mass throughout each major dinosaur clade was a complex mix of convergence and divergence associated with changes in multiple body segments (Figs 7, 8, 9, 10). Complete centre of mass data for each taxon, as well as full regression results and ancestral node reconstructions, are provided Tables [Link], [Link], [Link] and S6, and prominent overall trends are discussed here. Overall patterns in relative centre of mass between taxa were similar between model sets (Spearman's rho = 0.87–0.99 for anteroposterior measures of centre of mass, and 0.58–0.99 for dorsoventral measures of centre of mass, see also Figs S6–S10 for overall spatial patterns in centre of mass evolution). However, in the taxa with the most voluminous necks, non‐avian sauropsid centres of mass were markedly more anterior than other models (Figs S6–S10).

The anterior and ventral displacement of the centre of mass from the acetabulum in theropods were overall found to be negatively allometric relative to body mass. In most model sets, increasingly posterior centres of mass within theropods were found to be associated with greater tail mass, while more ventral centres of mass in theropods were most associated with greater forelimb segment masses. Strong associations were also found between more anterior centres of mass and increased head and forelimb masses in multiple theropod model sets, but these appear to be an artifact caused by the inclusion of the avialans *Archaeopteryx* and *Yixianornis*, which have proportionally large heads and forelimbs, and are thus considerably more front‐heavy than the other theropods (when the avialans were excluded, such relationships were not recovered, Table S6). Relative to the glenoid and acetabulum, the ancestral centre of mass in Theropoda (Figs 7 and 8) was very close to the reconstructed ancestral state at the base of Dinosauria, but more dorsal relative to total body mass to the power of one‐third. Centre of mass shifted posteriorly at the Neotheropoda node. Centre of mass evolution throughout Neotheropoda was characterised by an anterior shift at the *Dilophosaurus* + Averostra node, followed by slight posterior reversions towards Tetanurae and Avetheropoda, and finally a series of pronounced anteroventral shifts towards Avialae (Fig. 8). Several multi‐tonne theropod taxa convergently departed from the overall trend *via* strong posterodorsal reversions in centre of mass. Centres of mass in the non‐avialan maniraptoriform taxa shifted posteriorly from overall trends, but with the exception of *Nothronychus*, remained more ventral than other taxa. Relative to the glenoid and acetabulum, centres of mass were more anteroposteriorly restricted in non‐avialan theropods than other dinosaurian clades (Figs 7A and 8A), although were still disparate. For example, in the preferred isometric model set, anteroposterior centres of mass ranged from very close to the acetabulum (e.g. *Acrocanthosaurus*) to approximately 40% of glenoacetabular distance (*Dilophosaurus*) (Fig. 8). The total range of relative centres of mass skewed more ventrally in theropods than in the other dinosaur clades, particularly when measured relative to the glenoid and acetabulum (Figs 7 and 8). This largely results from *Struthiomimus* and the pennaraptorans – several of which notably have proportionally elongate and massive forelimbs/wings (e.g. *Archaeopteryx*, *Yixianornis*) or hindlimbs (e.g. *Struthiomimus*, *Anzu*), as well as reduced tails (e.g. *Yixianornis*). Centres of mass between the major dinosaur clades overlapped to a greater extent when calculated relative to total body mass to the power of one‐third (Fig. 7B).

In sauropodomorphs, the anterior displacement of the centre of mass from the acetabulum was overall found to be positively allometric, although with confidence intervals narrowly encompassing isometry in the bird allometric and preferred allometric model sets. In most model sets, increasingly anterior centres of mass in sauropodomorphs were most associated with greater neck masses. In the bird allometric and preferred allometric model sets, which reconstructed many sauropods with minimally slim necks (see Section VI.1), neck mass was less strongly associated with anteroposterior centres of mass than in other model variants, whereas greater torso mass was found to have a relatively strong association with more anterior centres of mass. Increasingly posterior centres of mass in sauropodomorphs were associated with greater tail mass, as well as hind limb mass. The ancestral centre of mass in Sauropodomorpha (Figs 7 and 9) was anteroposteriorly close to the ancestral dinosaurian condition, but more dorsal relative to the acetabulum and glenoid, and more ventral relative to total body mass to the power of one‐third. An anterior shift in centre of mass towards the glenoid occurred between non‐sauropodan sauropodomorphs and neosauropods (Fig. 9). Highly posterior centres of mass in some sauropods (e.g. *Diplodocus*, *Dicraeosaurus*, *Neuquensaurus*) represented convergent reversions from the ancestral neosauropod condition, whereas very front‐heavy sauropods (mamenchisaurids and various titanosauriforms) represented convergent anterior shifts in centre of mass from the ancestral eusauropod condition (Fig. 9).

In ornithischians, the confidence intervals of the centre of mass allometric slopes overlapped with isometry, indicating that relative displacement of the centre of mass from the acetabulum was not strictly tied to body size. Within ornithischians, more posterior centres of mass were most associated with greater tail mass, and more anterior centres of mass were most associated with greater forelimb masses. More ventral centres of mass in ornithischians were associated with larger torso, shank, and foot mass. Larger head mass was also found to be positively associated with more anterior centres of mass in the OLS regressions of multiple ornithischian model variants, but not in the PGLS regressions. Positive OLS associations between head mass and anteroposterior centre of mass in ornithischians appear to result solely from the ceratopsians, which have uniquely massive heads relative to other front‐heavy ornithischians (when ceratopsians were excluded, such relationships were not recovered, Table S6). The ancestral centre of mass in Ornithischia (Figs 7 and 10) was more posterior and more dorsal relative to the glenoid, and more ventral when normalised to body mass to the power of one‐third than the ancestral dinosaurian condition. A relatively posterior centre of mass was retained at the base of Thyreophora and Cerapoda, although the ancestral cerapodan centre of mass was more ventral (Fig. 10). Centres of mass in both eurypodan thyreophorans and ceratopsians convergently shifted anteriorly from the ornithischian ancestral condition, but eurypodan centres of mass were more dorsal (Fig. 10). In ornithopods, ancestral state reconstructions showed a posterior reversion in centre of mass at the base of Iguanodontia; however, the relatively early diverging iguanodontian *Dysalotosaurus* was found to have a considerably more posterior centre of mass than the majority of other dinosaurs studied here, and may thus be an outlier from the overall body proportion evolutionary trends across Ornithopoda. Hadrosauriform centres of mass were mostly less anterior relative to the ancestral ornithischian condition than other secondarily quadrupedal ornithischians (Fig. 10).

### (5) Proportional variation in dinosaur body segments

**Fig. 11:**
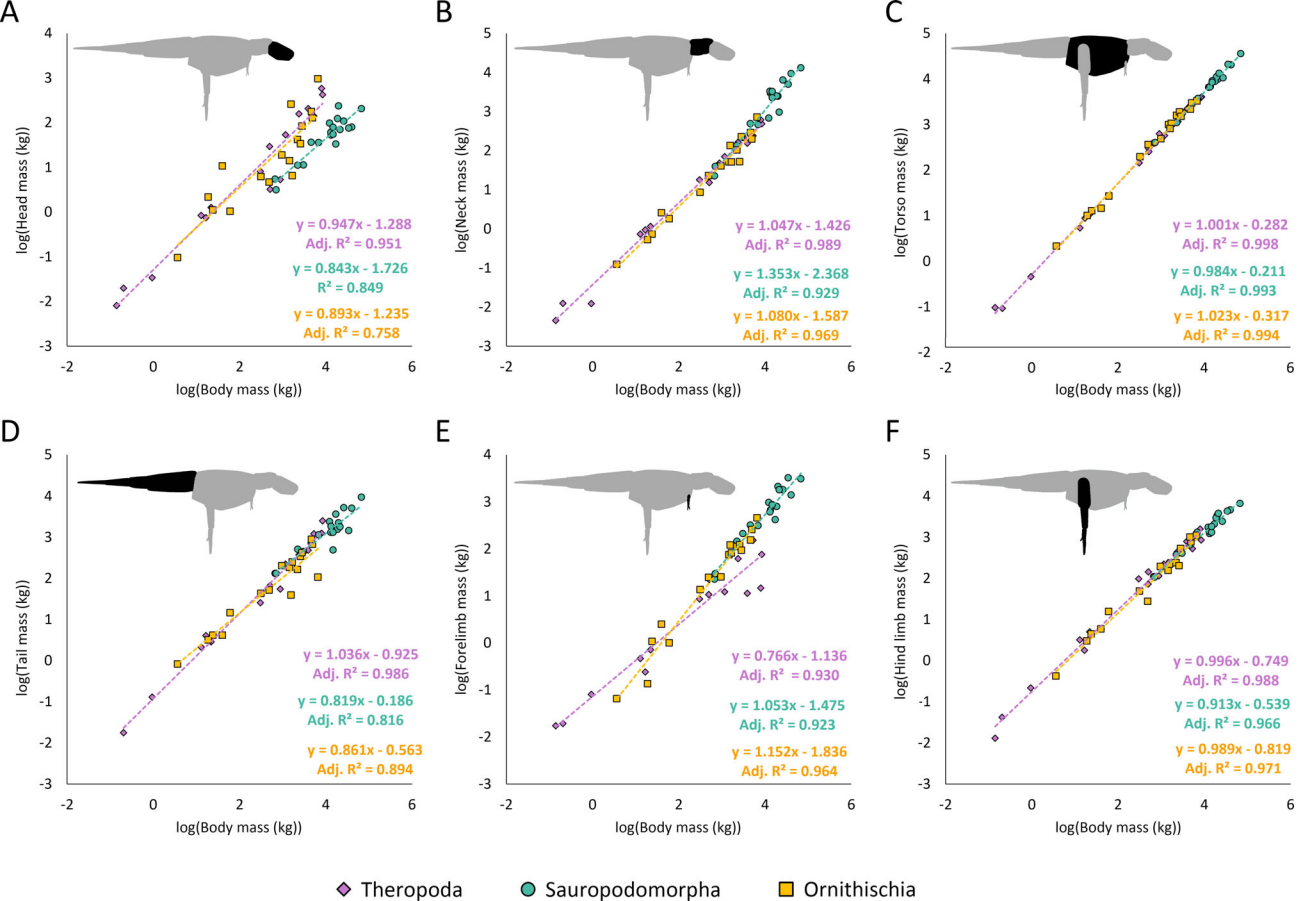
Relationships between body segment masses and total body masses across Dinosauria, using the preferred isometric model set: (A) head mass, (B) neck mass, (C) torso mass, (D) tail mass, (E) forelimb mass, (F) hind limb mass. Limb segment masses in E and F defined based on the sum of the left and right limbs.

Allometric scaling relationships between reconstructed body segment masses and total body masses between each of the major dinosaur clades were found to be variable (Fig. 11). Scaling relationships between body segment dimensions and total body masses were mostly found to have similar allometric exponents across different model sets. Complete body segment regression coefficients, as well as non‐dimensionalised body segment lengths and segment masses for all model variants, are provided in Tables S2 and S6 with major allometric trends and gross relative differences highlighted here.

**Fig. 12:**
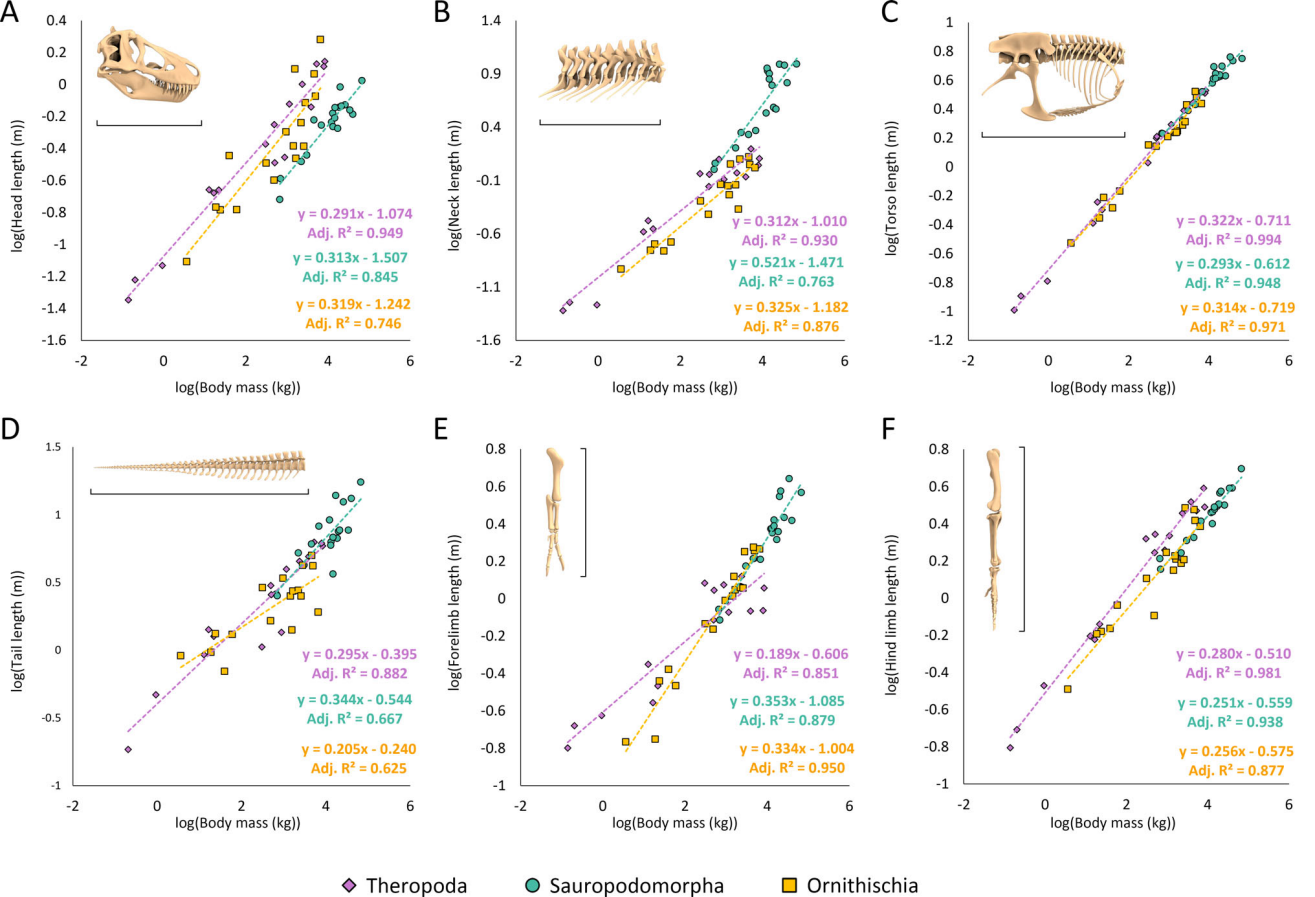
Relationships between body segment lengths and total body masses across Dinosauria, using the preferred isometric model set: (A) head length, (B) neck length, (C) torso length, (D) tail length, (E) forelimb length, (F) hind limb length.

In all dinosaur clades, torso masses had a tightly constrained isometric relationship with total body mass (Fig. 11C). In theropods, forelimb segment masses and lengths were strongly negatively allometric (Figs 11E and 12E). While most other body segment dimensions overall either scaled with weak allometry or close to isometry, considerable variation was still found among taxa. For example, in the preferred isometric model set, relative theropod head mass ranged from <1% of body mass in *Nothronychus*, to 10% in *Archaeopteryx*. Relative hindlimb masses in the preferred isometric model set ranged from ~10% of body mass in *Yixianornis*, *Coelophysis*, *Suchomimus*, and *Acrocanthosaurus*, to ~30% in *Struthiomimus* and *Anzu*. In the theropods from which tail dimensions were measured independently from torso size (which excludes *Yixianornis*, following the bird convex hulling protocol of Macaulay *et al*., 2023), relative tail masses in the preferred isometric model set ranged from 9% of body mass in *Archaeopteryx*, to 30% in *Acrocanthosaurus*.

In sauropodomorphs, neck lengths were overall found to scale with strong positive allometry (Fig. 12B), although the PGLS confidence intervals included isometry close to their lower boundaries. Sauropodomorph neck masses and tail masses overall scaled with positive allometry and negative allometry, respectively (Fig. 11A,D), with the broad confidence intervals from multiple model sets also including isometry close to their boundaries. This indicates a considerable spectrum of variability in sauropodomorph neck and tail dimensions that is somewhat independent from overall body size. For example, in the preferred isometric model set, relative sauropodomorph neck mass ranged from less than 5% of body mass in *Plateosaurus* and *Atlasaurus* to over 20% in the mamenchisaurids, *Apatosaurus*, *Barosaurus*, and *Rapetosaurus*. Relative sauropod tail mass in the preferred isometric model set ranged from 3% of body mass in *Rapetosaurus* to ~20% in the non‐sauropodan sauropodomorphs, *Patagosaurus*, diplodocines, and *Neuquensaurus*. Confidence intervals around sauropodomorph forelimb segment allometric slopes broadly encompassed isometry, indicating considerable variability in relative forelimb size as body mass increased. For example, in the preferred isometric model set, relative forelimb mass in sauropodomorphs ranged from 2% of body mass in *Diplodocus* to ~10% in *Jobaria* and *Giraffatitan*. By contrast, sauropodomorph hind limb segment lengths and masses scaled with either negative allometry or close to isometry.

In ornithischians, most body segment dimensions scaled either close to isometrically or showed considerable variation relative to body size (i.e. broad slope confidence intervals). Ornithischian tail masses and tail lengths overall scaled with negative allometry (Figs 11D and 12D), while ornithischian forelimb segment masses overall scaled with positive allometry (Fig. 11E), but only when bipedal taxa were included, with confidence intervals broadly encompassing isometry when only quadrupedal taxa were examined. Within large quadrupedal ornithischians, relative forelimb masses were thus highly variable. For example, ceratopsids possessed proportionally more massive forelimbs (e.g. 7–8% of body mass in the preferred isometric model set) than similarly large hadrosaurs (e.g. ~3% of body mass in the preferred isometric model set), with stegosaur and ankylosaur relative forelimb masses being found to be somewhat intermediate (e.g. ~5% of total body mass in the preferred isometric model set). Ornithischian hind limb lengths scaled with negative allometry (Fig. 12F), but again only when bipedal taxa were included. Among quadrupedal ornithischians, the distal hind limb segments of hadrosaurs and other quadrupedal iguanodontians were longer and proportionally more massive than other large taxa. For example, when normalised to body mass to the power of one‐third in the preferred isometric model sets, the pes of *Edmontosaurus* was found to be 1.6 and 2.7 times as long as its contemporaries *Triceratops* and *Denversaurus*, respectively.

## VI. DISCUSSION

### (1) Evaluating remaining subjectivity in dinosaur body models

Due to their quantitative basis in measured relationships between the hard skeletal tissues and soft tissues of extant sauropsids, the Macaulay *et al*. (2023) scaling factors allow for more empirically derived estimates of specific body segment volumetric properties than has been possible with previous methods. However, even when applied to accurately reconstructed skeletons, these scaling factors do not remove subjectivity entirely.

The body densities of non‐avian dinosaurs represent the largest unknown factor in any approach, with density estimates based on the comparative anatomy of extant tetrapods representing the most objective point of reference. Reconstructing differential density across the body likely represents the most realistic approach for representing the inertial properties of specific body regions, as it accounts for the localised presence of zero‐density air spaces, but mostly appears to have a relatively small effect on whole‐body centre of mass compared to homogenous densities, which is consistent with prior assessments (Bates *et al*., 2009a; Allen *et al*., 2009; Macaulay *et al*., 2017, 2023; Durston, Mahadik & Windsor, 2022). While included here for the sake of comparison, the lowest densities presented in our sensitivity analysis were based on flying birds of relatively small body sizes and with inflated air sacs, and are thus unlikely to be broadly applicable to most fossil archosaurs. For non‐avian dinosaurs, higher density values similar to extant taxa with more terrestrial habits are likely to be more realistic.

Subjectivities in segment‐specific densities are most apparent when attempting to reconstruct dinosaur necks, particularly in sauropods. Due to the inferred presence of extensive pneumatic diverticula throughout the cervical vertebrae of sauropods (e.g. Schwarz *et al*., 2007b), some studies have suggested neck density values as low as ~300 kg/m^3^ (e.g. Wedel, 2005; Henderson, 2006, 2013). However, recent measurements taken from bird cadavers (e.g. Macaulay, 2018; Bishop *et al*., 2021a), which are similarly known to possess cervical air sacs, as well as subjective sculpted reconstructions of sauropod airways based on high‐resolution skeletal models (Larramendi *et al*., 2021) suggest that neck density values considerably below our initial value of 800 kg/m^3^ may be unrealistic for sauropods. The lower neck density value of 500 kg/m^3^ used in our sensitivity analysis falls within the range of bird neck densities measured by Durston *et al*. (2022), however, these values also incorporated feather volumes, and are thus less applicable to extinct model estimates derived from the Macaulay *et al*. (2023) hull expansions, which do not incorporate feathers. Ultimately, the effect of this uncertainty on estimated body mass properties is mostly modest, as with the exception of the most extremely proportioned taxa, dinosaur necks are only a relatively small proportion of their total body volume. This uncertainty is only considerably amplified in the reconstructions of centre of mass in sauropods with particularly voluminous necks, even in comparison to other sauropods (e.g. mamenchisaurids, *Barosaurus*, and some titanosaurs), with lower neck density estimates corresponding to notably more posterior centres of mass. However, even if reconstructed with relatively low‐density necks, body mass distribution in sauropods is nonetheless highly variable, especially when compared to bipedal dinosaurs, as several taxa are still found to have highly anteriorly skewed centres of mass.

The Macaulay *et al*. (2023) extant sauropsid data set contains taxa ranging from 0.005 kg to 105 kg in total body mass, representing a considerable range of extant body sizes. However, the vast majority of non‐avian dinosaurs greatly exceed the skeletal dimensions of extant sauropsids, with resultant average body mass estimates for very large taxa exceeding the maximum masses of the Macaulay *et al*. (2023) data set by two orders of magnitude. Body masses predicted from the Macaulay *et al*. (2023) allometric equations are thus predicated on the assumption that fitted size‐relative trends from a sample of extant taxa can be considerably extrapolated beyond the upper body size limits of those taxa. The isometric expansions therefore provide an alternative but nonetheless anatomically grounded method of estimating body segment volumes that minimises potential artifacts of allometric extrapolation. Note, however, that discrepancies between isometric and allometric estimates of total body mass are minor (total isometric model masses generally either overlap with or approach the mPPE margins of the corresponding allometric models, Figs 3, 4, 5). The relative macroevolutionary patterns in centre of mass evolution are also consistent between isometric and allometric model sets (Figs S6–S10).

A possible drawback of the convex hull expansion factors is that they cannot precisely predict detailed mass distribution and shape within a single body segment, which can be subjectively depicted by the more naturalistic soft tissue outlines of sculpted or manually drawn volumetric reconstructions. In our estimates, the centre of mass of each individual body segment is therefore kept constant between the minimal hulls and different expansion iterations. Ultimately, the schematic nature of convex hulls is unlikely to be a major quantitative issue when estimating overall dimensions in a static context. Analyses of extant tetrapod CT data by both Macaulay *et al*. (2023) and Coatham *et al*. (2021) have shown that the centres of mass of minimally hulled individual skeletal segments are, relative to segment size, mostly only minimally different from the centres of mass of the real soft tissue segments. Macaulay *et al*. (2023) also showed that the effect of any larger discrepancies between the segment‐specific centres of mass of the convex hulls and the real soft tissue segments (e.g. the more proximal centres of mass of distally tapered bird limbs relative to the convex hulls) on whole‐body centres of mass are also very small. This, in spite of their relatively abstract shapes, increases confidence in the use of convex hulls to estimate whole‐body dimensions and overall centres of mass accurately. In more complex dynamic models, such as those used in simulations of specific functions (e.g. feeding behaviour or gait) subjective, manually modelled reconstructions of body segment outlines may still be necessary in order to estimate more realistic moments of inertia in body segments for which specific shape is important to consider (Kilbourne & Hoffman, 2013). However, empirically derived convex hull expansion factors such as those discussed here would nonetheless still act as a valuable guideline for the upper and lower bounds of the overall dimensions of these manual reconstructions.

Disparity in dinosaur body forms suggests that a given set of expansion factors should not be uniformly applied to all taxa indiscriminately. Some non‐avian dinosaurs may have body segment morphologies more closely analogous to extant birds, whereas others may have morphologies more closely analogous to extant non‐avian sauropsids, hence the definition of subjectively preferred expansion combinations in our models. These similarities and differences may result from either functional convergence or phylogenetic affinity. For example, the forelimbs of winged pennaraptorans bear a closer osteological resemblance to their extant bird relatives than the weight‐bearing forelimb bones of many quadrupedal dinosaurs, which often bear greater resemblance, at least superficially, to other non‐avian sauropsids. Other expansions may reflect morphologies specific to the extant group from which they were defined. The recovery of mostly more voluminous torso volumes when using bird‐based expansions than using non‐avian sauropsid expansions may be the result of most extant birds having far more massive pectoral muscles than non‐avian sauropsids as an adaptation to powered flight, with the pectoralis of flighted birds alone making up between 8 and 11% of body mass, compared to less than 1% of body mass in crocodylians (Biewener, 2011, Allen *et al*., 2014).

**Fig. 13:**
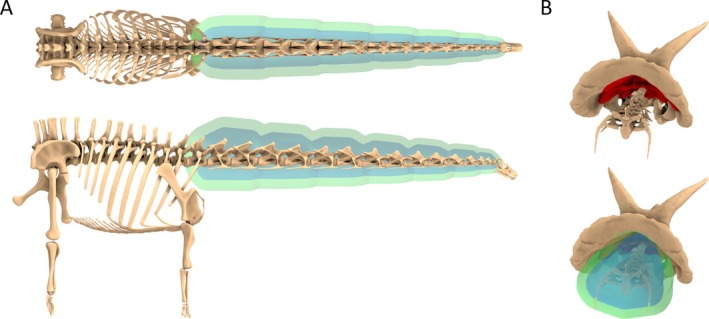
Illustrations demonstrating the varied suitability of different expansion factors for different dinosaurs, based on the average non‐avian sauropsid (green) and average bird (blue) isometric neck expansion factors in *Diplodocus* (A) and *Triceratops* (B). The red‐shaded region in B marks approximate total inferred origin area of neck musculature. The bird‐based neck volumes are herein considered to be more appropriate for sauropods, while the non‐avian sauropsid neck volumes are considered to be more appropriate for ceratopsians. While the silhouettes illustrated here are an abstract visualisation that corresponds with the values predicted by the expansion factors rather than a precise depiction of *in‐vivo* soft tissue outlines, the overall volumes nevertheless reflect empirical observations of soft tissue dimensions in extant sauropsids.

Dinosaur necks are another good example of a body region where the soft tissue scaling relationships of extant sauropsids should be considered with care. The Macaulay *et al*. (2023) scaling factors demonstrate that non‐avian sauropsids have far greater neck soft tissue volumes relative to the size of their cervical vertebrae than birds. However, with the exception of a small handful of taxa such as giant tortoises, which were not included in the Macaulay *et al*. (2023) soft tissue volume data set, extant non‐avian sauropsids seldom possess the proportionally long necks of many extant birds and extinct sauropsid clades (Wilkinson & Ruxton, 2012). Applying extant non‐avian sauropsid soft tissue expansion factors to the necks of dinosaurs with large cervical vertebrae thus produces extremely large neck volumes, considerably increasing both neck mass and total body mass (Fig. 13A, Table S4). In addition to the stark dissimilarity to extant long‐necked taxa, extant non‐avian sauropsid neck expansion factors produce extreme whole‐body centres of mass that may even be placed anterior to the shoulder joint in the longest necked taxa (Figs S7, S8), which provides an objective basis to consider these values unrealistic. While extant non‐avian sauropsid necks are therefore unlikely to be suitable analogues for reconstructing the necks of certain dinosaurs, there are a number of probable exceptions. Ceratopsians, for example (Fig. 13B), which have very large skulls relative to their cervical vertebrae, can be inferred from impressions on the posterior surface of their frills to have possessed extensive neck musculature (Tsuihiji, 2010). Hadrosaurs, which have relatively slender cervical vertebrae, can also be inferred from osteological correlates and examples of exceptional preservation to have possessed extensive neck soft tissues (Bertozzo *et al*., 2020).

While seemingly more applicable to long‐necked dinosaurs, using bird neck soft tissues as the basis for dinosaur models presents its own caveats. Macaulay *et al*. (2023) found that bird neck soft tissue volumes scaled with a much more negative allometric exponent relative to the skeleton than other axial body segments, meaning that the allometric bird neck expansion factors might consequently produce unexpectedly slender necks in very large dinosaurs. In fact, when extrapolated to sauropods with minimal neck skeletal hulls greater than a cubic metre in volume, the allometric bird neck expansion factors produce volumes smaller than the skeletal convex hulls, which represent a minimal threshold for the reconstruction of the soft tissue envelope. While the minimal hulls do contain empty convex space which could feasibly be redistributed or collapsed before the skeletal segments become entirely ‘shrink‐wrapped’, application of the strong negative allometric soft tissue scaling exponents to the largest sauropod necks produces volumes too small for this to be possible. Potential issues with negatively allometric expansion factors further highlight the utility of the average isometric expansion factors of Macaulay *et al*. (2023), or even the minimal hull volumes themselves where feasible, as alternative plausible options for body segment volume reconstruction.

Ultimately, relative overall patterns in body shape evolution (Figs S6–S10) and the ranked ordering of total body masses (Spearman's rho >0.99, Table S2) were found to be consistent in each of our variant model sets. Therefore, while the use of a preferentially selective approach to body volume reconstructions based on different extant sauropsid expansion factors may subjectively produce more realistic masses and proportions for a given taxon than uniformly applied expansions, this is unlikely to affect overall conclusions about dinosaur body shape and size evolution, as was found for theropod‐specific evolutionary patterns by Macaulay *et al*. (2023).

### (2) Comparing our model masses with previous volumetric soft tissue reconstructions

The primary benefit of the Macaulay *et al*. (2023) expansion factors is that they produce anatomically grounded volumes for specific body segments, addressing the uncertainty of previous approaches in which soft tissue outlines were sculpted or drawn more subjectively, and expanded to a range of values homogenously (e.g. Bates *et al*., 2009a,b, 2012; Hutchinson *et al*., 2011; Allen *et al*., 2013). Some variation in total mass between our models and other volumetric methods may be explained by subjective differences in skeletal articulation (resulting in differences in gross skeletal dimensions), or by different assumptions regarding overall density (e.g. the definition of overall segment densities as opposed to manual reconstruction of air spaces). However, many instances of previous models being lighter than or close to the lower end of our estimates (Figs 3, 4, 5) are likely due to their reconstructed soft tissue outlines adhering very tightly to skeletal landmarks (e.g. Paul, 1997; Henderson, 2006, 2023; Larramendi *et al*., 2021), particularly around the torso, hips, and neck. In many extant sauropsids, these regions support thick layers of muscle, fat, and integument, which are at least partly accounted for in our models by the Macaulay *et al*. (2023) expansions.

In most taxa, our reconstructed total body masses were only closely comparable with conservatively outlined models when we used minimal densities derived from flying birds (Tables S2 and S5), which are not only much lower than most previous non‐avian dinosaur models, but are unlikely to be applicable to non‐volant taxa. Most of our models were also heavier than models produced *via* the 21% expansion approach of Sellers *et al*. (2012), suggesting that extant sauropsids overall have proportionally greater soft tissue masses relative to their skeletons than the large mammals (90–2735 kg) from which the 21% expansion factors were derived (Figs 3, 4, 5) – note however that the body masses used by Sellers *et al*. (2012) were estimated based on taxon‐specific scaling equations rather than direct specimen measurements. While fewer taxa could be compared, general agreement between our model mass ranges and expanded elliptical/octagonal hooping models (e.g. Allen *et al*., 2013) reflects the more generous soft tissue volumes applied *via* the expanded hooping approach than the slimmer 3D‐slicing models.

Greater body masses in our models than previous methods are unlikely to be the result of over‐extrapolating extant soft tissue positive allometric exponents to larger dinosaurs, as the same differences are also observed when comparing previous reconstructions to allometric mass estimates for small dinosaurs well within the size ranges of extant sauropsids (e.g. Figures 3A and 5A). The models derived from the average isometric expansion factors are also considerably heavier than most previous models (Figs 3, 4, 5). If we assume that non‐avian dinosaur soft tissues were morphologically comparable to the extant sauropsids that phylogenetically bracket them, we can therefore conclude from our models that many previous reconstructions are likely to be too slim.

### (3) Placing our results within the context of limb bone scaling trends

Overlap between the mass ranges of our models and the MASSTIMATE stylopodial estimates demonstrates an overall encouraging agreement between methods, suggesting that relative to their limb bone dimensions, dinosaur body masses generally fell within the typical range of variation of extant tetrapods. However, assessing the variability of this overlap in different dinosaurs may be useful for inferring differences in form and function that are not apparent from limb bone shaft dimensions alone. Convergently quadrupedal (but morphologically disparate) dinosaur groups provide an interesting example for discussion here. In several sauropods, our volumetric model mass estimates were very close to the point estimates of the MASSTIMATE equations (e.g. *Apatosaurus* MASSTIMATE = 41 tonnes, *Apatosaurus* preferred isometric model = 40 tonnes; Fig. 4), whereas our volumetric mass estimates of several large ornithischians were considerably lower (e.g. *Stegosaurus* MASSTIMATE = 3.7 tonnes, *Stegosaurus* preferred isometric model = 1.7 tonnes) (Fig. 5). This distinction was also highlighted by Campione & Evans (2012, 2020) when comparing previous volumetric estimates, and suggests that these ornithischians had proportionally very robust limb bones for their body mass. This may reflect differences in limb mechanics between clades. In quadrupedal dinosaurs, posture can be inferred from multiple lines of musculoskeletal evidence to have been highly disparate (e.g. Wilson & Carrano, 1999; Maidment *et al*., 2012, 2014a,b; Maidment & Barrett, 2012a,b; Dempsey *et al*., 2023), which may have resulted from limitations imposed by their morphologically variable ancestors (Dempsey *et al*., 2023) in addition to later divergences in overall bauplans and limb functions (Maidment *et al*., 2014a,b). Irrespective of the specific ecomorphological drivers of this disparity, the limbs of different quadrupedal dinosaur clades would likely have experienced a varied suite of loading regimes. Perhaps the inferred abducted or bent‐elbow postures of ceratopsians and stegosaurs (e.g. Maidment & Barrett, 2012a; Dempsey *et al*., 2023) led to their limb bones experiencing greater torsional and multi‐directional bending loads across their shafts (e.g. Garcia & da Silva, 2006), leading to an adaptive increase in their relative limb bone thickness compared to sauropods, which are generally considered to have retained more columnar limbs, even in taxa with wide‐gauge stances (e.g. Wilson & Carrano, 1999). Similar observations were made by Wright *et al*. (2024), who found that volumetrically estimated body masses in large sprawling Permo‐Triassic tetrapods were also lower than would have been predicted by stylopodial scaling estimates.

**Fig. 14:**
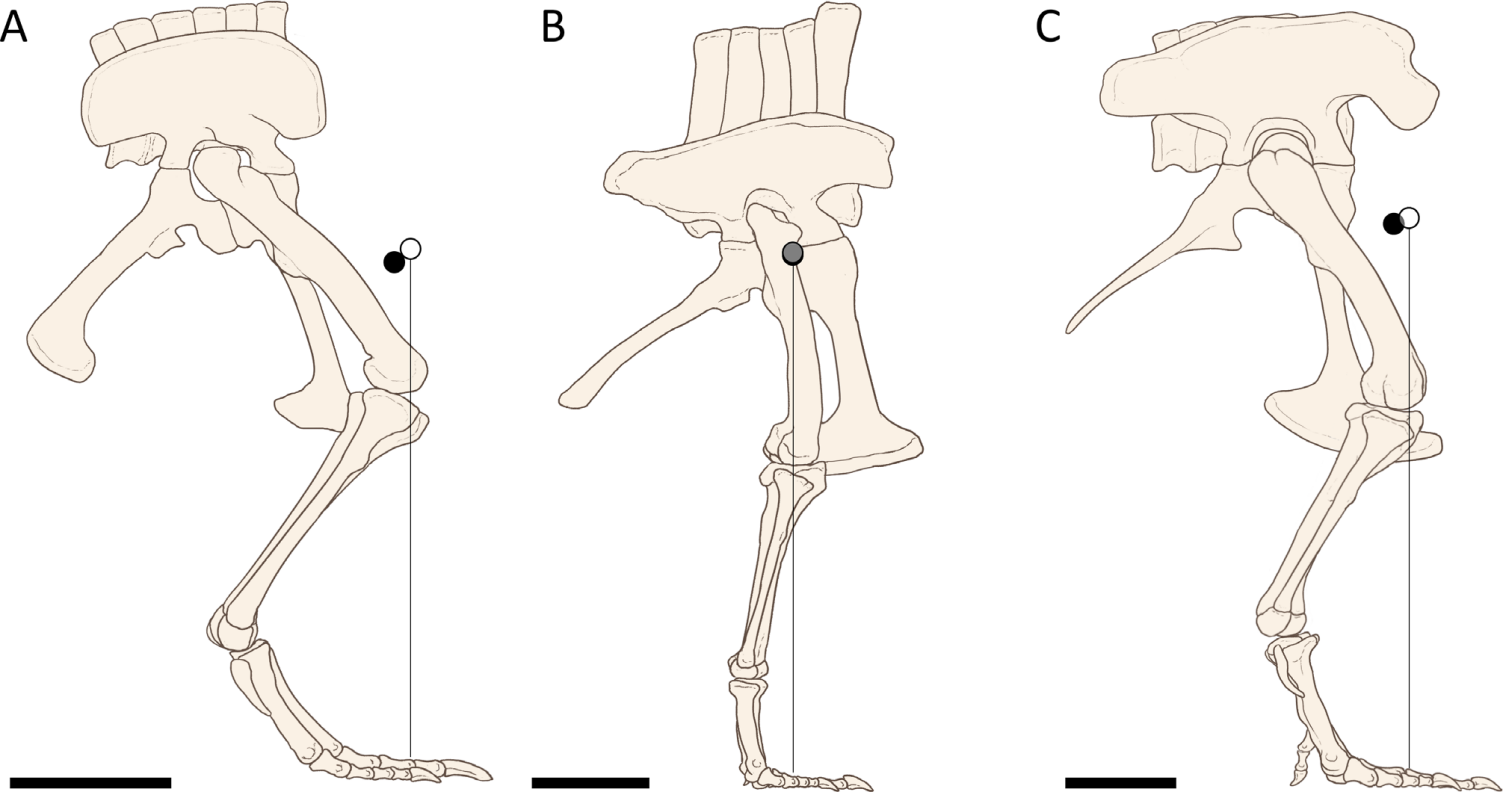
Illustrations showing hypothetical large theropod standing postures, approximated from the overall flexion required to bring the knee and foot below the whole‐body centres of mass. Centres of mass shown are based on the preferred isometric models of this study, with the black circle representing the centre of mass of the model in the reference pose, and the white circle representing the centre of mass when the hindlimbs are positioned as illustrated. (A) *Sinraptor*, (B) *Acrocanthosaurus*, (C) *Tyrannosaurus*. Scale bars = 0.5 m.

Variations in posture and locomotion may also explain major differences between volumetric model masses and stylopodial equation masses in large bipedal dinosaurs. For example, *Tyrannosaurus* and *Acrocanthosaurus* can, from their similar overall skeletal dimensions, be reasonably assumed to have been similar in body mass. Indeed, in the models constructed for this study, average body masses for each were close to 9 tonnes (Tables S2 and S4). However, due to its relatively narrow femoral shaft, the stylopodial mass estimate for *Acrocanthosaurus* is considerably lower than volumetric estimates (Table S2). *Tyrannosaurus* volumetric mass estimates on the other hand are reasonably agreeable with stylopodial estimates (Fig. 3D). Perhaps *Acrocanthosaurus*, which has previously been highlighted for its unusual limb proportions among large theropods (Gatesy & Middleton, 1997), did not require particularly robust femora to bear its weight, and instead solved the mechanical demands of large body size by adopting a more upright limb posture than *Tyrannosaurus* (Fig. 14), which would mitigate bending stresses across the limb shafts, as well as reducing external moments across the joints (Gatesy *et al*., 2009). Some support for this postural difference is found in the centre of mass estimates, which are more posterior in *Acrocanthosaurus* than *Tyrannosaurus* in most model sets (Figs 8 and 14, Table S2). At the more extreme end of these differences, the anteroposterior centre of mass in the preferred isometric model of *Acrocanthosaurus* is only 6% of glenoacetabular distance, compared to 25% in *Tyrannosaurus* (Figs 8 and 14, Table S2). If such estimates are accurate, then as well as reducing the magnitude of external loads applied to the hip joint, the more posterior centre of mass of *Acrocanthosaurus* would mean that compared to *Tyrannosaurus*, its hind limb would not need to be as strongly flexed for the foot to be placed below the centre of mass, which is a requirement for static stability in bipeds (Fig. 14) (Gatesy *et al*., 2009). The narrower limb bone shafts of *Acrocanthosaurus* may also reflect a greater decline in locomotor performance with increasing body size in carcharodontosaurs than in tyrannosaurs (see also Section VI.5). While multiple studies have cast considerable doubt on the fast running ability of *Tyrannosaurus* (e.g. Hutchinson & Garcia, 2002; Hutchinson, 2004; Hutchinson *et al*., 2007, 2011; Gatesy *et al*., 2009; Sellers *et al*., 2017), the robust shafts of its femur may still have been associated with the greater mechanical demands of greater locomotor performance (e.g. higher peak stresses) than similarly sized carcharodontosaurs, although without more complex mechanical modelling the extent of these hypothesised differences cannot be determined from body segment dimensions alone. While *Acrocanthosaurus* itself may be a more extreme case study, it is nonetheless probable that the femora of different large bipedal dinosaurs were proportionally variable relative to body mass, and that many additional factors drove different femoral shaft dimensions.

### (4) Interpreting centre of mass evolution across Dinosauria

As single‐data‐point summaries of total body proportions, and critical determinants of locomotion and posture, whole‐body centres of mass are a useful reference point for interpreting form–function relationships in a wide evolutionary context, as has been demonstrated in many previous studies (e.g. Allen *et al*., 2013; Maidment *et al*., 2014b; Bates *et al*., 2016; Clemente *et al*., 2017; Bishop *et al*., 2020, Macaulay *et al*., 2023). We found that each major dinosaur clade was characterised by complex webs of convergence and divergence in their centres of mass (Figs 7, 8, 9, 10), suggesting that their body shape evolution was driven by a broad range of ecomorphological factors.

With the exclusion of volant taxa, bipedal dinosaurs were reconstructed with a more posteriorly constrained range of centres of mass relative to glenoacetabular distance than in quadrupeds (Figs 7, 8, 9, 10), which is agreeable with trends found in previous studies (e.g. Bishop *et al*., 2020). This reflects the constraints of a stable bipedal body plan during stance, in which the centre of mass cannot be so anterior that the foot/feet of the supporting limb(s) cannot be placed beneath it (Gatesy *et al*., 2009). The convergent evolution of markedly posterodorsal centres of mass in multiple massive theropods (e.g. spinosaurids, allosauroids, tyrannosaurids), which deviate from the gradual anterior shift in theropod centre of mass towards the bird line (Fig. 8), suggests that large body size imposed further constraints on theropod mass distribution. More posterior centres of mass in the heaviest theropods would have reduced the magnitude of the external moments applied to the hip joint (Gatesy *et al*., 2009).

Despite these general constraints, our models reveal surprising disparity in the centres of mass of theropods (Fig. 14) and other bipedal dinosaurs, ranging from very close to the acetabulum to over 40% of the anteroposterior glenoacetabular distance (Figs 7 and 8, Table S2). More detailed biomechanical modelling and consideration of additional factors such as muscle moment arms and joint ranges of motion would be required to quantify precisely the exact effects of this disparity on reconstructed postures (e.g. Gatesy *et al*., 2009). Yet, by following the base assumption that a more anterior centre of mass requires a more flexed/crouched hind limb in bipedal dinosaurs (Fig. 14) (e.g. Christiansen & Bonde, 2002; Allen *et al*., 2013, Gatesy *et al*., 2009), we can speculate about functional differences between taxa and the evolutionary constraints and drivers behind them. In volant theropods (i.e. birds and their closest relatives), a relatively flexed hind limb was necessitated by the evolution of more front‐heavy body forms (Gatesy, 1990; Christiansen & Bonde, 2002; Allen *et al*., 2013; Macaulay *et al*., 2023), whereas a more ventral centre of mass might have been conducive to stability during flight (Thomas & Taylor, 2001). The evolutionary drivers and constraints of postural disparity in non‐volant bipedal dinosaurs would likely have been numerous, ranging from the reduction of high joint or soft tissue loads in taxa with more upright limbs, to the benefits of dynamic stability during fast movement or greater jumping performance afforded by a more flexed limb (Biewener, 1989b; Gatesy & Biewener, 1991; Blum *et al*., 2011; Bishop *et al*., 2021b). An alternative interpretation is that relatively anterior centres of mass in bipeds may reflect variation in the habitual pitching of the torso. For example, in the therizinosaur *Nothronychus*, which has a highly anterior centre of mass compared to other theropods (Fig. 8), a pitched body may have brought the centre of mass in line with the knee and foot without the hind limb needing to be highly flexed. While distinct from typically reconstructed large theropod postures, this would have been advantageous to browsing behaviour, which is consistent with other herbivorous dietary adaptations in therizinosaurs (Zanno *et al*., 2009). This would also be consistent with the hypothesis of Smith & Gilette (2023), who proposed based on the morphology of the acetabulum that the pelvis of *Nothronychus* would have been held at a greater incline than less front‐heavy theropods.

Multiple large ornithischians and sauropods trend towards highly anterior centres of mass, corroborating previous models (e.g. Henderson, 2006; Maidment *et al*., 2014b) and starkly contrasting with much more posterior centres of mass in the largest theropods (Figs 7, 8, 9, 10). In large bipedal dinosaurs, the need to mitigate external loads upon the hind limb was likely to have been a key driver of body shape evolution, whereas the ability to bear weight on all four limbs would have relaxed physical constraints on the body plans of large quadrupedal dinosaurs. Centre of mass evolution and the initial onset of quadrupedality in different dinosaur clades was likely to have been driven by varying ecological and behavioural factors. For example, in sauropodomorphs, the evolution of heavier necks and more anterior centres of mass as body and feeding envelope size increased would have necessitated the use of their forelimbs to support increasingly anterior loads. In ceratopsians, the proportionally massive heads associated with highly anterior centres of mass were already present in small early‐diverging taxa (Fig. 10), and appear to have evolved in association with both specialised powerful jaws and elaborate cranial ornamentation (Maidment *et al*., 2014b; Nabavizadeh, 2023). Anterior shifts in ceratopsian centres of mass may therefore have been driven by both feeding ecology and, depending on the interpreted function of the cranial ornamentation, socio‐sexual signalling (e.g. Knapp, Knell & Hone, 2021) or defensive factors (Nabavizadeh, 2023). In contrast to other quadrupedal ornithischians, most quadrupedal ornithopods retain a relatively posterior centre of mass (Fig. 10), corroborating previous models (Maidment *et al*., 2014b). This suggests retention of some bipedal capability, and may imply that the initial evolution of ornithopod quadrupedality took place as a result of ecological drivers distinct from other quadrupedal dinosaurs. Hadrosaurs, for example, are hypothesised to have engaged in long‐distance migratory behaviour (Prierto‐Márquez, 2010), and thus may have mechanically benefitted from the ability to distribute their weight across all four limbs during the longest and most challenging journeys.

### (5) Interpreting variation in dinosaur body segment proportions

The reconstructed masses of individual body segments may provide focused perspectives on dinosaur form and function, particularly their relationships with total body mass. Limb segment masses are particularly informative, as given that a large proportion of a limb segment's volume is expected to be made up of muscle, our reconstructed dinosaur limb segment masses can be interpreted as reasonable proxies for total limb muscle masses, as in previous works (e.g. Bates *et al*., 2009a,b, 2012; Hutchinson *et al*., 2007, 2011). For example, a lack of strong positive allometry in theropod hind limb masses and lengths (Fig. 11) corroborates previous studies suggesting that locomotor performance was not maintained across increases in body size, and that large theropods in general were not well adapted for high‐speed locomotion (Hutchinson & Garcia, 2002; Hutchinson, 2004; Hutchinson *et al*., 2007, 2011; Gatesy *et al*., 2009; Bates *et al*., 2012; Sellers *et al*., 2017). However, functionally informative differences between similarly massive taxa can still be inferred. Proportionally short, light hind limbs in *Suchomimus* and *Acrocanthosaurus* compared to similarly massive tyrannosaurids are consistent with other lines of evidence summarised both herein and in previous studies (e.g. Snively & Russell, 2003; Persons & Currie, 2016; Snively *et al*., 2019), suggesting that tyrannosaurids were, at least relatively, more agile in their habits than other large theropods. However, it is also possible that the shorter stride lengths and smaller hind limb muscles of *Suchomimus*, *Acrocanthosaurus,* and similar taxa were compensated for by their massive *caudofemoralis longus* muscles (which is a key hip extensor, see Gatesy, 1990), inferred here from the large reconstructed tail volumes, and possibly further supported in spinosaurids by the notably large transverse processes of the anterior caudals seen in some specimens (e.g. Ibrahim *et al*., 2020; Sereno *et al*., 2022). Further benefits of large tail muscles in these taxa may also have included stabilisation of the tail and trunk during locomotion (e.g. Díez Díaz *et al*., 2020). Negative allometry in hind limb mass may also have had certain benefits to very large dinosaurs such as sauropods. For example, a relatively less massive limb may have been less energetically expensive to swing (e.g. Kilbourne & Hoffman, 2013). While the energetics of non‐avian dinosaurs are not well understood, it is therefore possible that the evolution of proportionally less massive limbs in the largest taxa compromised speed for metabolic benefits, driven by the need to optimise the cost of supporting and moving their heavy bodies. As with our speculations on posture, more sophisticated modelling and simulation techniques could be used to test these ideas in future studies.

Differences in relative limb segment dimensions in quadrupedal dinosaurs corroborate previous studies suggesting that the locomotion of each convergently quadrupedal clade would have been distinctly different (e.g. Wilson & Carrano, 1999; Maidment *et al*., 2012, 2014a,b, Maidment & Barrett, 2012a,b; Dempsey *et al*., 2023). For example, our hadrosaur models have both longer and more massive distal hind limb segments relative to body mass than their ceratopsid and ankylosaurian contemporaries, likely indicating greater stride lengths, as well as proportionally more massive ankle extensor muscles. This is consistent with previous studies identifying adaptations to a more cursorial, parasagittal gait in hadrosaurs than other ornithischians (e.g. Maidment *et al*., 2012, 2014a,b, Maidment & Barrett 2012a,b; Dempsey *et al*., 2023), which may have assisted in travel over longer distances, or, in the absence of clear defensive structures, aided in the evasion of predators. In ceratopsians, proportionally more massive forelimb segments than other quadrupeds may reflect their splayed, wider‐gauge postures, inferred previously from reconstructed muscle moment arms and trackway evidence (Maidment & Barrett, 2012a; Dempsey *et al*., 2023). While beneficial for stability in wider‐bodied taxa (Henderson, 2006), splayed postures result in greater torsional and mediolaterally oriented loads being placed upon the limb than narrower postures (Wilson & Carrano, 1999; Garcia & da Silva, 2006). The need to equilibrate this loading may therefore have driven the evolution of more massive forelimb muscles and generally more robust forelimb osteology in ceratopsians. Differences in limb dimensions may also be associated with factors other than locomotor performance. For example, proportionally longer forelimbs in *Giraffatitan* and *Atlasaurus* than similarly massive sauropods would have expanded the total verticality of their feeding envelope, allowing them to feed on vegetation beyond the reach of other herbivores.

Variation in axial segment dimensions may also reflect ecological and behavioural distinctiveness. For instance, while sauropod neck lengths and masses were overall positively allometric, the ~7 m long neck of *Apatosaurus* is found to be considerably heavier than the longer necks of several other sauropods (e.g. the 8–10 m long necks of the mamenchisaurids, *Barosaurus*, *Giraffatitan* and *Sauroposeidon*/*Paluxysaurus*). The high volume of apatosaurine necks relative to other sauropods results from well‐developed protuberances of the cervical vertebrae and ribs (Woodruff, 2016; Wedel & Taylor, 2023), each of which likely indicate relatively massive cervical muscles. Rather than being used for high browsing as is often hypothesised for the sauropods with the longest necks (Christian, 2010), the robust, muscular neck of apatosaurines may instead have been associated with a more mechanically demanding feeding style requiring tight control or strong flexion of the neck as it moved across a wide area (Stevens & Parrish, 1999; Stevens, 2013; Woodruff, 2016; Wedel & Taylor, 2023), with some speculating that it may even have been adapted to intraspecific combat (Taylor *et al*., 2015).

### (6) Potential future applications of our models

While empirically determined segment‐specific mass properties aid in the interpretation of dinosaur form and function at the broadest scale, they also highlight the opportunity for future investigation. There is a great deal of potential in this modelling approach to inform biomechanical models, particularly those produced *via* multi‐body dynamic methods, in which segment‐specific masses and inertial properties are critical to simulating the dynamics of motion. Many of the hypotheses raised herein about posture, joint loading, and locomotor performance could be tested in more intricate detail by the production of such models. However, estimation of segment mass properties is only one half of the puzzle – dynamic simulations of locomotion and posture also require the specific architectural properties of the muscles driving the motion of those segments to be estimated, among other biological properties. We herein considered total limb segment masses to be a reasonable proxy for muscle masses. However, it is possible that differential scaling of the constituent components within a given limb segment, such as higher muscle mass to fat mass ratios, or a greater ratio of extensors to flexors, would significantly affect hypotheses about locomotion, or at least offer more detailed quantitative perspectives. Further research and modelling efforts could seek to use data‐driven, well‐evaluated methods of total soft tissue volume reconstruction in conjunction with similarly quantified relationships between skeletal geometries and muscle properties, offering a highly holistic and integrative approach to the study of dinosaur form and function.

## VII. CONCLUSIONS

(1) By using a whole‐body modelling approach underpinned by relatively objective, segment‐specific scaling factors between skeletal volumes and soft tissue volumes derived from extant sauropsids (Macaulay *et al*., 2023), we produced anatomically grounded body mass and centre of mass estimates for a representative data set of 52 non‐avian dinosaurs. The use of empirical data from extant taxa addresses subjectivities in previous volumetric approaches.
(2) Our model mass estimates suggest that if non‐avian dinosaur soft tissue anatomy scaled comparably to extant sauropsids, then previous more conservatively reconstructed soft tissue envelopes are likely to be too minimal. Our reconstructed range of whole‐body centres of mass are also, at least in theropods, more anteroposteriorly constrained than previous subjectively defined maximally front‐heavy and maximally rear‐heavy models.
(3) The ability to derive empirical constraints on body segment volumes highlights the utility of extant anatomical data to reduce uncertainty in the reconstruction of extinct organisms. This expansion upon previous hybrid approaches that combine volumetric modelling methods with extant‐scaling approaches [e.g. Sellers *et al*. (2012) and subsequent studies] represents a major step towards a unified framework (Campione & Evans, 2020) for dinosaur body dimension estimation.
(4) Our workflow produces dinosaur body masses consistent with the range of variability expected from relationships between body mass and limb bone shaft size in extant tetrapods, suggesting overall agreement between different extant scaling methods. However, volumetric models show the potential to differentiate the masses of fossil taxa within that expected variability, allowing for more direct comparison and interpretation of their body dimensions from both a whole‐body and segment‐specific perspective.
(5) The evolution of dinosaur body proportions and mass distribution was a complex web of convergence and divergence, likely driven by numerous ecomorphological factors. For example, proportional variation within theropods likely reflects differences in their locomotion, posture, and hunting/feeding strategies. Sauropodomorphs and ornithischians are more proportionally disparate than theropods, especially at large sizes, reflecting relaxed constraints on body morphology and the ability to proliferate into a broader range of niches when weight can be supported on four limbs.
(6) In addition to allowing for gross comparisons among taxa, empirically grounded volumetric models such as those presented here have the future potential to underpin complex dynamic simulations of dinosaur posture and gait.

## Supporting information


**Table S1.** Primary reference specimens, reconstruction notes, and model sources for each of the skeletal models used in this study.
**Appendix S1**. Macaulay *et al*. (2023) hull expansions used in the ‘preferred’ methods.
**Fig. S1**. Alternative convex hull models for *Acrocanthosaurus*.
**Fig. S2**. Whole‐body centre of mass in *Stegosaurus* in the preferred isometric model variant, with major osteoderms included (black circle) and excluded (white circle).
**Fig. S3**. Whole‐body centre of mass in *Chasmosaurus* in the preferred isometric model variant, with cranial ornamentation at 2000 kg/m^3^ (black circle) and 1000 kg/m^3^ (white circle).
**Fig. S4**. Whole‐body centres of mass in *Omeisaurus* with the neck in the reference pose (black circle) and the neck pitched at 45° (white circle).
**Fig. S5**. Time‐calibrated phylogenetic tree of the taxa modelled in this study.
**Table S4**. Total body masses for each model variant, using the preferred density approach.
**Fig. S6**. Phylomorphospace scatter plots illustrating whole‐body centre of mass evolution across Dinosauria, based on the preferred allometric model set.
**Fig. S7**. Phylomorphospace scatter plots illustrating whole‐body centre of mass evolution across Dinosauria, based on the non‐avian sauropsid allometric model set.
**Fig. S8**. Phylomorphospace scatter plots illustrating whole‐body centre of mass evolution across Dinosauria, based on the non‐avian sauropsid isometric model set.
**Fig. S9**. Phylomorphospace scatter plots illustrating whole‐body centre of mass evolution across Dinosauria, based on the bird allometric model set.
**Fig. S10**. Phylomorphospace scatter plots illustrating whole‐body centre of mass evolution across Dinosauria, based on the bird isometric model set.

**Appendix S2.** Phylogenetic tree used in analyses.

**Table S2.** Complete body segment length, volume, and centre of mass data for each primary model set.

**Table S3.** Node dates used to construct the phylogenetic tree, and centre of mass ancestral state reconstructions.

**Table S5.** Data from variant models used in the sensitivity analyses.

**Table S6.** Complete results for all body segment and centre of mass regressions.

## Data Availability

Convex hull model files are provided at the following repository https://doi.org/10.6084/m9.figshare.27283401.
