# Supplementary material for: New perspectives on body size and shape evolution in dinosaurs
Source: Biol Rev Camb Philos Soc. 2025 May 8;100(5):1829–60. doi: 10.1111/brv.70026 (PMC12407065; doi:10.1111/brv.70026)
Supplement: Supplementary file 1 — Table S1. Primary reference specimens, reconstruction notes, and model sources for each of the skeletal models used in this study. Appendix S1. Macaulay et al. (2023) hull expansions used in the ‘preferred’ methods. Fig. S1. Alternative convex hull models for Acrocanthosaurus. Fig. S2. Whole‐body centre of mass in Stegosaurus in the preferred isometric model variant, with major osteoderms included (black circle) and excluded (white circle). Fig. S3. Whole‐body centre of mass in Chasmosaurus in the preferred isometric model variant, with cranial ornamentation at 2000 kg/m3 (black circle) and 1000 kg/m3 (white circle). Fig. S4. Whole‐body centres of mass in Omeisaurus with the neck in the reference pose (black circle) and the neck pitched at 45° (white circle). Fig. S5. Time‐calibrated phylogenetic tree of the taxa modelled in this study. Table S4. Total body masses for each model variant, using the preferred density approach. Fig. S6. Phylomorphospace scatter plots illustrating whole‐body centre of mass evolution across Dinosauria, based on the preferred allometric model set. Fig. S7. Phylomorphospace scatter plots illustrating whole‐body centre of mass evolution across Dinosauria, based on the non‐avian sauropsid allometric model set. Fig. S8. Phylomorphospace scatter plots illustrating whole‐body centre of mass evolution across Dinosauria, based on the non‐avian sauropsid isometric model set. Fig. S9. Phylomorphospace scatter plots illustrating whole‐body centre of mass evolution across Dinosauria, based on the bird allometric model set. Fig. S10. Phylomorphospace scatter plots illustrating whole‐body centre of mass evolution across Dinosauria, based on the bird isometric model set. [file BRV-100-1829-s002.docx]

**New perspectives on body size and shape evolution in dinosaurs**

Matthew Dempsey, Samuel R. R. Cross, Susannah C. R. Maidment, John R. Hutchinson & Karl T. Bates

**SUPPORTING INFORMATION**

**Table S1.** Primary reference specimens, reconstruction notes, and model sources for each of the skeletal models used in this study.

| Taxon | Group | Primary reference specimen | Reconstruction notes | Skeleton model source |
| --- | --- | --- | --- | --- |
| *Marasuchus* | Non-dinosaurian Dinosauriformes | NHMUK PV R 14101 | Reconstructed from laser scan of near-complete individual, supplemented with new digitally sculpted ribcage with dimensions estimated based on *Silesaurus* (Piechowski & Dzik, 2010). | Previously in Allen *et al.* (2013) |
| *Staurikosaurus* | Theropoda | MCZ 1669 | Digital sculpt by Orlando Grillo based on measurements; reconstructed missing elements are consistent with *Herrerasaurus*. | Previously in Macaulay *et al.*(2023); Allen *et al.* (2013); Grillo & Azevedo (2011). |
| *Coelophysis* | Theropoda | CMNH 10971 | Reconstructed from laser scan of complete composite mount. | Previously in Macaulay *et al.* (2023); Bishop *et al.* (2021*b*); Allen *et al.* (2013). |
| *Dilophosaurus* | Theropoda | UCMP 37302 | Reconstructed from laser scan of near-complete individual, ribcage/chest dimensions scaled after *Coelophysis*, + digitally sculpted hallux. | Previously in Macaulay *et al.* (2023); Allen *et al.* (2013). |
| *Suchomimus* | Theropoda | MNBH GAD500 | Composite reconstruction from computed tomography (CT) data, based on several individuals, with sculpted elements inferred from other spinosaurids. | Sereno *et al.* (2022) *via* MorphoSource (https://www.morphosource.org/projects/000460619) |
| *Sinraptor* | Theropoda | ZDM 0024 | Reconstructed from laser scan (point cloud) of ZDM mounted skeleton, which is a near-complete individual with reconstructed forelimbs and distal hind limb segments that are proportionally consistent with other allosauroids. | Clauss *et al.* (2016) *via* Morphobank https://morphobank.org/index.php/Projects/ProjectOverview/project_id/2404 |
| *Allosaurus* | Theropoda | SMA 0005 | Digital sculpt scaled to previous scan data (Macaulay *et al.* 2023; Allen *et al.* 2013; Bates *et al.* 2012). | New for this study |
| *Acrocanthosaurus* | Theropoda | NCSM 14345 | Reconstructed from laser scan of mounted cast skeleton, which is based primarily on a single individual with elements restored based on other referred specimens. | Previously in Bates *et al.* (2009*b*, 2012). |
| *Gorgosaurus* | Theropoda | NMC 2120 | Digital sculpt based on published measurements and photographs of a near-complete specimen, supplemented with similarly sized individuals (Lambe, 1914; Russell, 1970; Currie, 2003). | New for this study |
| *Tyrannosaurus* | Theropoda | AMNH 5027 | Digital sculpt based on scan data and measurements from multiple equivalently sized near-complete specimens (e.g. AMNH 5027, USNM 555000/MOR 555) (Clauss *et al.*, 2016; Digitisation Program Office, Smithsonian Institution, 2019*a*). | New for this study |
| *Struthiomimus* | Theropoda | BHI 1266 | Reconstructed from laser scan of complete mounted cast skeleton. | Previously in Macaulay *et al.* (2023); Allen *et al.* (2013); Bates *et al*. (2009*b*) |
| *Nothronychus* | Theropoda | MSM P2117 | Composite reconstruction primarily based on the holotypes of *Nothronychus mckinleyi* and *Nothronychus graffami*, incorporating laser scans from Hedrick *et al.* (2015), rescaled skull of *Erlikosaurus* from Lautenschlager (2015), and sculpted elements following the measurements of Hedrick *et al.* (2015) and Kirkland & Wolfe (2001), also referencing CT data of the iliosacral block provided by David K. Smith (see Smith & Gillette, 2023). | New for this study |
| *Anzu* | Theropoda | CM 78001 | Digital sculpt by Jason Adam Bannister based on measurements of two individuals (CM 78000 and CM 78001). | Previously in Macaulay *et al.* (2023); Allen *et al.* (2013). |
| *Microraptor* | Theropoda | IVPP V13352 | Scan of physical sculpt by Jason Brougham based on measurements of complete specimen. | Previously in Macaulay *et al.* (2023); Allen *et al.* (2013). |
| *Velociraptor* | Theropoda | IGM 100/986 | Reconstructed from laser scan of near-complete mounted skeleton, missing tail length reconstructed based on other dromaeosaurid specimens (e.g. Ostrom 1969; Hone *et al.*, 2021). | Previously in Macaulay *et al.* (2023); Allen *et al.* (2013). |
| *Archaeopteryx* | Theropoda | MfN MB.Av.101 | Scan of physical sculpt by Janice Hertel based on measurements of complete specimen. | Previously in Macaulay *et al.* (2023); Allen *et al.* (2013). |
| *Yixianornis* | Theropoda | IVPP V12631 | Reconstructed from CT data of complete specimen, shape of crushed skull estimated based on other avialans. | Previously in Macaulay *et al.* (2023); Allen *et al.* (2013). |
| *Plateosaurus* | Sauropodomorpha | (IFGT) GPIT-PV-30784 | Reconstructed from CT data of near-complete skeleton. | Previously in Macaulay *et al.* (2023); Allen *et al.* (2013); Mallison (2010). |
| *Lufengosaurus* | Sauropodomorpha | IVPP V15 | Reconstructed from laser scan of BMNH mounted cast of near-complete skeleton. | Clauss *et al.* (2016) *via* Morphobank (https://morphobank.org/index.php/Projects/ProjectOverview/project_id/2404). |
| *Patagosaurus* | Sauropodomorpha | PVL 4170 | Reconstructed from photogrammetry of composite mounted skeleton of multiple individuals. Mounted skull is a hypothetical reconstruction, but gross proportions are consistent with other early-diverging non-neosauropodan sauropods (e.g. *Shunosaurus*). | Previously in Bates *et al.* (2016). |
| *Shunosaurus* | Sauropodomorpha | ZDM T5404 | Reconstructed from laser scan of near-complete ZDM mounted skeleton. | Clauss *et al.* (2016) *via* Morphobank (https://morphobank.org/index.php/Projects/ProjectOverview/project_id/2404). |
| *Mamenchisaurus* | Sauropodomorpha | ZDM 0126 | Reconstructed from laser scan of near-complete ZDM mounted skeleton, which is mostly complete, with some sculpted elements. Dimensions cross-referenced with Yong *et al.* (2001) and Young & Zhao (1972). | Clauss *et al.* (2016) *via* Morphobank (https://morphobank.org/index.php/Projects/ProjectOverview/project_id/2404). |
| *Omeisaurus* | Sauropodomorpha | ZDM T5701 | Reconstructed from laser scan of near-complete ZDM mounted skeleton, which is a composite of ZDM T5701-T5704 (He *et al.*, 1988). | Clauss *et al.* (2016) *via* Morphobank (https://morphobank.org/index.php/Projects/ProjectOverview/project_id/2404). |
| *Jobaria* | Sauropodomorpha | MNN TIG | Reconstructed from photogrammetry of near-complete mounted skeleton. | Previously in Bates *et al.* (2016). |
| *Apatosaurus* | Sauropodomorpha | CM 3018 | Digital sculpt scaled to previous scan data (Bates *et al.*, 2016) and with reference to Gilmore (1936). | New for this study |
| *Diplodocus* | Sauropodomorpha | CM 84 | Digital sculpt scaled to previous scan data and measurements primarily derived from CM 84, supplemented with data from multiple equivalently sized specimens (e.g. CM 94, USNM 10865, YPM 1906) (Clauss *et al.*, 2016; McIntosh & Carpenter, 1998; Holland, 1905). | New for this study. |
| *Barosaurus* | Sauropodomorpha | AMNH 6341 | Reconstructed from photogrammetry of near-complete mounted skeleton. Mounted skull is based on *Diplodocus*. Digitally reconstructed pedal elements based on *Diplodocus*. | Previously in Bates *et al.* (2016). |
| *Dicraeosaurus* | Sauropodomorpha | MfN MB.R.4886 | Reconstructed from photogrammetry of near-complete mounted skeleton, which has a sculpted skull. | Previously in Bates *et al.* (2016). |
| *Amargasaurus* | Sauropodomorpha | MACN N-15 | Reconstructed from photogrammetry of near-complete mounted skeleton, with tail length additionally digitally reconstructed based on *Dicraeosaurus*. Mounted skull is largely reconstructed, but is consistent with the morphology of other dicraeosaurids. | Previously in Bates *et al.* (2016). |
| *Camarasaurus* | Sauropodomorpha | AMNH 664 | Digital model by Kent Stevens based on measurements of near-complete skeleton. | Previously in Bates *et al.* (2016); Stevens (2013). |
| *Atlasaurus* | Sauropodomorpha | Holotype, Musée des sciences de la Terre de Rabat unnumbered | Reconstructed from laser scan of mounted near-complete skeleton. Missing posteriormost portions of tail digitally sculpted based on *Giraffatitan*. | Clauss *et al.* (2016) *via* Morphobank (https://morphobank.org/index.php/Projects/ProjectOverview/project_id/2404). |
| *Giraffatitan* | Sauropodomorpha | MfN MB.R.2181 | Reconstructed from photogrammetry of mounted near-complete skeleton derived mostly from a single individual, with sculpted elements. | Previously in Bates *et al.* (2016). |
| *Paluxysaurus*  (=*Sauroposeidon*?) | Sauropodomorpha | FWMSH 93B-10 | Reconstructed from photogrammetry of mounted composite skeleton, which has some sculpted elements. | Previously in Bates *et al.* (2016). |
| *Rapetosaurus* | Sauropodomorpha | FMNH PR 2255 | Reconstructed from photogrammetry of mounted FMNH PR 2209 subadult-type skeleton, scaled up based on femur length to match the largest adult specimens (FMNH PR 2255). | Previously in Bates *et al.* (2016). |
| *Neuquensaurus* | Sauropodomorpha | MLP composite | Reconstructed from photogrammetry of mounted composite skeleton. Mounted skull is a hypothetical reconstruction, rescaled here to match the proportions of titanosaurs with associated cranial and cervical material more closely (e.g. *Sarmientosaurus*; Martinez *et al.*, 2016) | Previously in Bates *et al.* (2016). |
| *Patagotitan* | Sauropodomorpha | MPEF-PV 3400 | Composite of surface scans from the holotype and paratype series, with some sculpted elements as mounted. Skull is a hypothetical reconstruction, rescaled here to match the proportions of similarly long-necked titanosaurs more closely (e.g. *Rapetosaurus*). | Previously in Carballido *et al.* (2017). |
| *Scutellosaurus* | Ornithischia | MNA PL175 | Digital sculpt by Stuart Pond based on photographs and measurements, and incorporating photogrammetric scan data. | Previously in Anderson *et al.* (2023). |
| *Gigantspinosaurus* | Ornithischia | ZDM 0019 | Reconstructed from laser scan of near-complete ZDM mounted skeleton. | Clauss *et al.* (2016) *via* Morphobank (https://morphobank.org/index.php/Projects/ProjectOverview/project_id/2404). |
| *Stegosaurus* | Ornithischia | NHMUK PV R36730 | Reconstructed from laser scans of a near-complete mounted skeleton. | Previously in Maidment *et al.* (2015); Brassey *et al.* (2015). |
| *Gastonia* | Ornithischia | USUEPM composite | Reconstructed from photogrammetry of mounted composite skeleton, with additional digitally sculpted tail elements following Kinneer *et al*. (2016). | Clauss *et al.* (2016) *via* Morphobank (https://morphobank.org/index.php/Projects/ProjectOverview/project_id/2404). |
| *Denversaurus* | Ornithischia | FPDM-V9673 | Digital sculpt by Stuart Pond based on scan data, photographs, and measurements, primarily from a near-complete skeleton cast by BHI (original skeleton housed at FPDM). | Sellers & Pond (2015) *via* figshare (https://figshare.com/articles/dataset/Kinect_controlled_dinosaur_simulations_for_education_and_public_outreach/2008977). |
| *Euoplocephalus* | Ornithischia | ROM 1930 | Reconstructed from photogrammetry of NMSF mounted cast skeleton. | Clauss *et al.* (2016) *via* Morphobank (https://morphobank.org/index.php/Projects/ProjectOverview/project_id/2404). |
| *Hypsilophodon* | Ornithischia | NHMUK PV R 5829 | Reconstructed primarily from laser scan of mounted near-complete skeleton, CT-based model of skull from Button *et al.* (2023), with additional sculpted elements following Galton (1974). | New for this study. |
| *Tenontosaurus* | Ornithischia | MANC LL.12275 | Reconstruction from photogrammetry of limb and girdle elements, with sculpted axial skeleton and manual/pedal elements based on additional photogrammetry and measurements. Near-complete individual with minor missing elements supplemented by measurements from Forster (1990). | New for this study, forelimb and pectoral girdle scans previously in Dempsey *et al.* (2023). |
| *Dysalotosaurus* | Ornithischia | MfN MB.1 composite | Reconstructed from photogrammetry of mounted composite skeleton. | Scan data provided by Heinrich Mallison. |
| *Mantellisaurus* | Ornithischia | NHMUK PV R 5764 | Reconstructed from laser scans, supplemented with additional digitally sculpted axial and manual elements following Norman (1986). | Bonsor *et al.* (2023) *via* MorphoSource (https://www.morphosource.org/concern/biological_specimens/000394030). |
| *Iguanodon* | Ornithischia | IRSNB 1536 | Laser scan (point cloud) of NMSF mounted near-complete cast skeleton, supplemented with additional digitally sculpted tail elements following Norman (1980). | Clauss *et al.* (2016) *via* Morphobank (https://morphobank.org/index.php/Projects/ProjectOverview/project_id/2404). |
| *Brachylophosaurus* | Ornithischia | NMC 8893 | Digital sculpt based on measurements and photographs of NMC 8893 and MOR 794, (following Prieto-Marquez, 2001; Cuthbertson & Holmes, 2010), forelimb and pectoral girdle elements informed by scan data provided by Jordan Mallon, previously used in Dempsey *et al.* (2023). | New for this study. |
| *Edmontosaurus* | Ornithischia | AMNH 5886 | Reconstructed from photogrammetry of complete mounted skeleton, with digitally sculpted ischium. | Clauss *et al.* (2016) *via* Morphobank (https://morphobank.org/index.php/Projects/ProjectOverview/project_id/2404). |
| *Stegoceras* | Ornithischia | UALVP-2 | Composite reconstruction from laser scans of limb, girdle, and skull elements, with sculpted axial skeleton and distal limb elements based on *Homalocephale* following Maryańska & Osmólska (1974). | New for this study, scanned elements from Moore *et al*. (2022) and WitmerLab *via* MorphoSource (https://www.morphosource.org/concern/media/000018284). |
| *Protoceratops* | Ornithischia | MAS 100.502 | Reconstructed from photogrammetry of near-complete skeleton, with digitally sculpted ischium, caudalmost tail, phalanges, and broken rostral elements. | New for this study, scan data provided by Andrew Knapp. |
| *Chasmosaurus* | Ornithischia | NHMUK R4948 | Reconstructed from laser scans of skull, photogrammetry of humerus, antebrachium and pectoral girdle, with sculpted elements modifying *Triceratops* elements (see below) to match *Chasmosaurus* following Maidment & Barrett (2011) and related chasmosaurines (e.g. Holmes, 2014). | New for this study, skull scan data provided by Andrew Knapp, forelimb and pectoral girdle scans previously in Dempsey *et al.* (2023). |
| *Triceratops* | Ornithischia | USNM 4842 | Reconstructed from scan of composite mount USNM PAL500000, which was digitally rescaled prior to mounting proportionally to match USNM 4842, from which the largest proportion of the mount is derived. | Digitisation Program Office, Smithsonian Institution, 2019*b* (https://3d.si.edu/object/3d/triceratops-horridus-marsh-1889%3Ad8c623be-4ebc-11ea-b77f-2e728ce88125). |

**Institutional Abbreviations**

**AMNH** = American Museum of Natural History, New York City, US; **BHI** = Black Hills Institute of Geological Research; Hill City, US; **CM** = Carnegie Museum of Natural History, Pittsburgh, US; **CMNH** = Cleveland Museum of Natural History, Cleveland, USA; **FMNH** = Field Museum of Natural History, Chicago, US; **FPDM** = Fukui Prefectural Dinosaur Museum, Katsuyama, JP; **FWMSH** = Fort Worth Museum of Science and History, Fort Worth, US; **IFGT** = Institut für Geowissenschaften, Universität Tübingen, Tübingen, DE; **IGM** = Instituto de Geología, Universidad Nacional Autónoma de México, Mexico City, MX; **IRSNB** = Institut royal des Sciences naturelles de Belgique, City of Brussels, BE; **IVPP** = Institute of Vertebrate Paleontology and Paleoanthropology, Beijing, CN; **MACN** = Museo Argentino de Ciencias Naturales ‘Bernardino Rivadavia’, Buenos Aires, AR; **MANC** = Manchester Museum, Manchester, UK; **MAS** = Mongolian Academy of Sciences, Ulan Bator, MN; **MCZ** = Museum of Comparative Zoology, Harvard University, Cambridge, US; **MfN** = Museum für Naturkunde Berlin, Berlin, DE; **MLP** = Museo de La Plata, La Plata, AR; **MNA**; Museum of Northern Arizona, Flagstaff, US; **MNBH** = Musée National Boubou Hama, Niamey, NE; **MNN** = Musée National du Niger, Niamey, NE; **MPEF** = Museo Paleontológico Edigio Feruglio, Trelew, AR; **MSM** = Mesa Southwest Museum, Mesa, US; **NCSM**; North Carolina Museum of Natural Sciences; Raleigh, US; **NHMUK** = Natural History Museum, London, UK; **NMC** = Canadian Museum of Nature, Ottawa, CA; **PVL** = División de Paleontología de Vertebrados del Museo de Ciencias Naturales y Universidad Nacional de San Juan, San Juan, PR; **ROM** = Royal Ontario Museum, Toronto, CA; **SMA** = Sauriermuseum Aathal, Seegräben, CH; **UALVP** = University of Alberta Laboratory for Vertebrate Paleontology, Edmonton, CA; **UCMP** = University of California Museum of Paleontology, Berkeley, US; **USNM** = National Museum of Natural History, Smithsonian Institution, Washington DC, US; **USUEPM** = USU Eastern Prehistoric Museum, Price. US; **ZDM** = Zigong Dinosaur Museum, Zigong, CN.

**Appendix S1. Macaulay *et al.* (2023) hull expansions used in the ‘preferred’ models**

**Heads**

Non-avian sauropsid head expansions produced volumes slightly greater than the bird expansions, with the allometric bird equations leading to volumes slightly lower than the minimal hulls. This is not deemed to be infeasible, as non-concave shapes in the skulls of many taxa leave empty space in the minimal hulls which could hypothetically be collapsed or redistributed. In addition to variations in non-convex skull shapes, higher non-avian sauropsid head expansions may be due to an increased volume of extraoral tissue relative to birds. In the preferred models, non-avian sauropsid head expansions were therefore used for most taxa, with bird head expansions only being used for toothless beaked taxa (*Struthiomimus*, *Anzu*, *Yixianornis*).

**Necks**

As discussed in greater detail in the main text (Section VI.1, Fig. 13), non-avian sauropsid neck expansions lead to much greater neck volumes than bird neck expansions. With the exception of taxa such as ceratopsians and hadrosaurs, which can be inferred to have had very extensive neck soft tissues either *via* osteological correlates or examples of exceptional preservation (Tsuihiji, 2010; Bertozzo *et al.*, 2020), the preferred models used bird neck expansions. The negative allometry of the bird neck allometric expansion equations produced volumes lower than the minimal hulls in the largest sauropods (neck minimal hulls > 1 m^3^). Given that the neck minimal hulls adhered more tightly to overall skeletal forms than other segments, this was deemed infeasible, and thus the minimal hulls were used as a threshold which the reconstructed neck volumes could not fall below. For *Amargasaurus*, we followed the hypothesis of Cerda *et al.* (2022), and treated the elongate neural spines of the cervical vertebrae as supporting a pair of continuous sails. Each set of neural spines was hulled separately from the rest of the neck and set to a density of 1000 kg/m^3^.

**Torsos**

With the exception of the allometric expansions in very large dinosaurs with torso minimal hull volumes greater than ~4 m^3^, bird torso expansions produced greater volumes than the non-avian sauropsid torso expansions. This may be due to the more voluminous pectoral muscles of birds: the avian m. pectoralis makes up 8–11% of body mass, compared to less than 1% of body mass in crocodylians (Biewener, 2011; Allen *et al.*, 2014). With the exception of *Yixianornis*, which possessed a deep bird-like sternum which likely supported extensive pectoral musculature, non-avian sauropsid torso expansions were used in the preferred models.

**Tails**

Only non-avian sauropsid tail expansions were defined by Macaulay *et al.* (2023), as the convex hulling protocol enveloped the reduced tails of birds into the torso volumes. All models except *Yixianornis* therefore used non-avian sauropsid tail expansions.

**Forelimb segments**

While birds have extensive shoulder musculature, the expansions show that the more distal regions of their forelimbs support far less voluminous soft tissues than in non-avian sauropsids. In the preferred models, bird forelimb expansions were used for the pennaraptoran (winged) theropods, with other taxa using the non-avian sauropsid forelimb expansions.

**Hind limb segments**

Bird hind limb segment expansions produced lower volumes than non-avian sauropsid expansions. The distal segments of bird limbs are distally tapered and more tendinous, whereas the pelvic musculature of birds distinctly differs from non-avian sauropsids in many aspects, notably, the absence of several internal thigh muscles (Carrano & Hutchinson, 2002). With the exception of the avialans, the preferred models therefore used non-avian sauropsid thigh expansions. For highly digitigrade taxa with more elongate distal hind limb segments, in which much of the weight is expected to be supported by the pedal phalanges, bird expansions were used for the shanks and feet in the preferred models, with non-avian sauropsid expansions being used in taxa with shorter, more compact and functionally plantigrade feet (stegosaurs, sauropods). Due to the distal ends of bird tarsometatarsi typically being much wider than the narrow shafts, and the soft tissues of bird tarsometatarsi being largely tendinous and wrapping tightly to the bone, the bird metatarsal expansions produced volumes considerably lower than the minimal hulls, especially when the allometric equations were applied to large taxa. While the soft tissues around the metatarsals of digitigrade dinosaurs may indeed have been similarly minimal and tendinous, the metatarsals themselves lack the same ‘pinched’ shape as bird tarsometatarsi, meaning that there is far less empty space within the convex hulls that can be feasibly collapsed. In the bird-based models, the minimal convex hull volumes were therefore used instead to represent a minimal soft tissue volume. The feet of sauropods form a compact plantigrade unit in which the pedal phalanges are less independent from the overall volume of the foot. In several sauropods, limited scan resolution also meant that the feet could not be precisely divided into additional segments. An average of the metatarsal and pedal phalange expansion factors was therefore applied to single foot hulls in each sauropod model set.


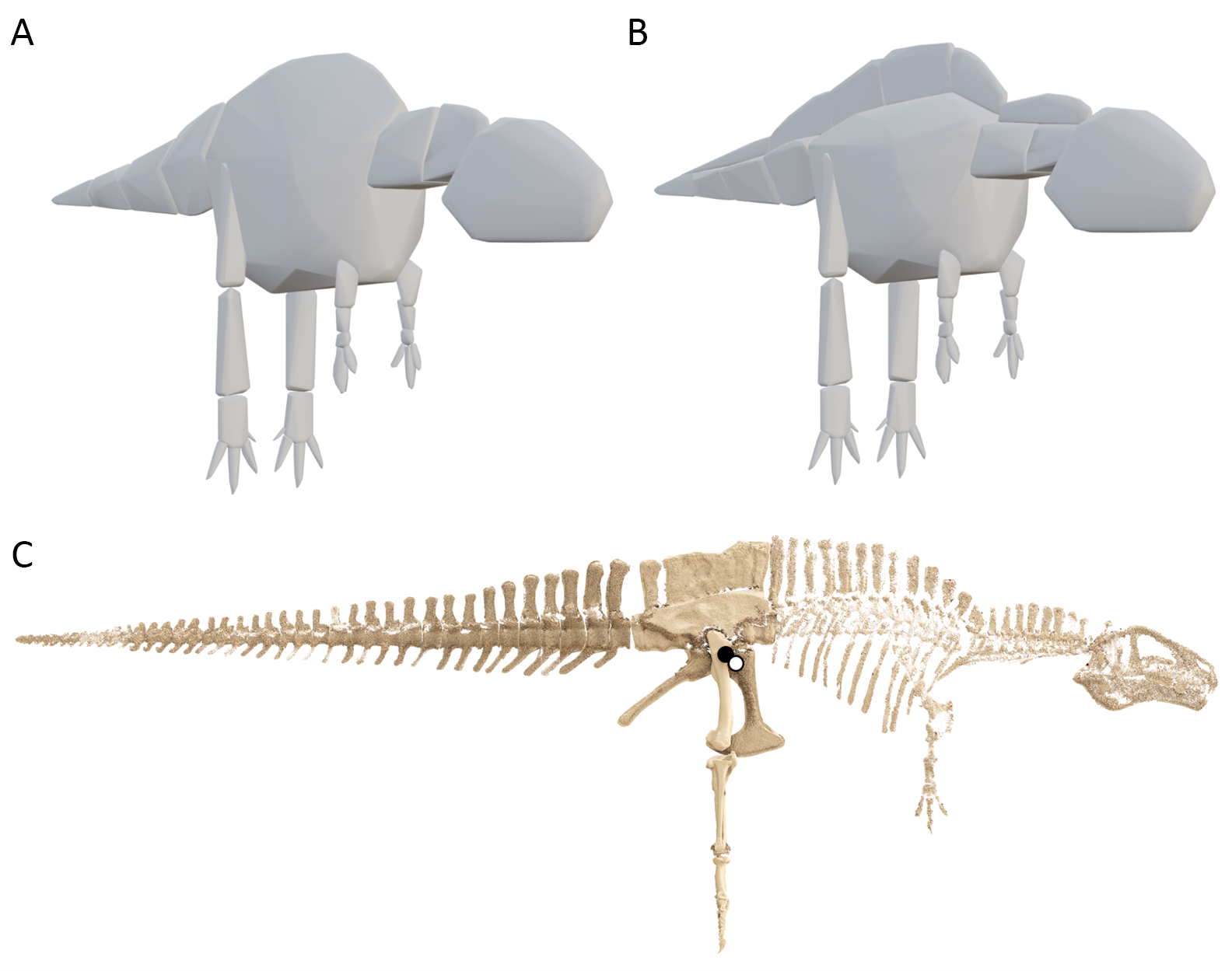


**Fig. S1.** Alternative convex hull models for *Acrocanthosaurus*. (A) ‘Hump-backed’ model, in which elongate neural spines are contained within the main axial hulls. (B) ‘sail-backed’ model, in which the elongate neural spines are separately hulled. (C) Whole-body centre of mass in *Acrocanthosaurus* using the preferred isometric workflow for the ‘hump-backed’ model (black circle) and ‘sail-backed’ model with a sail density of 1000 kg/m^3^ (white circle).


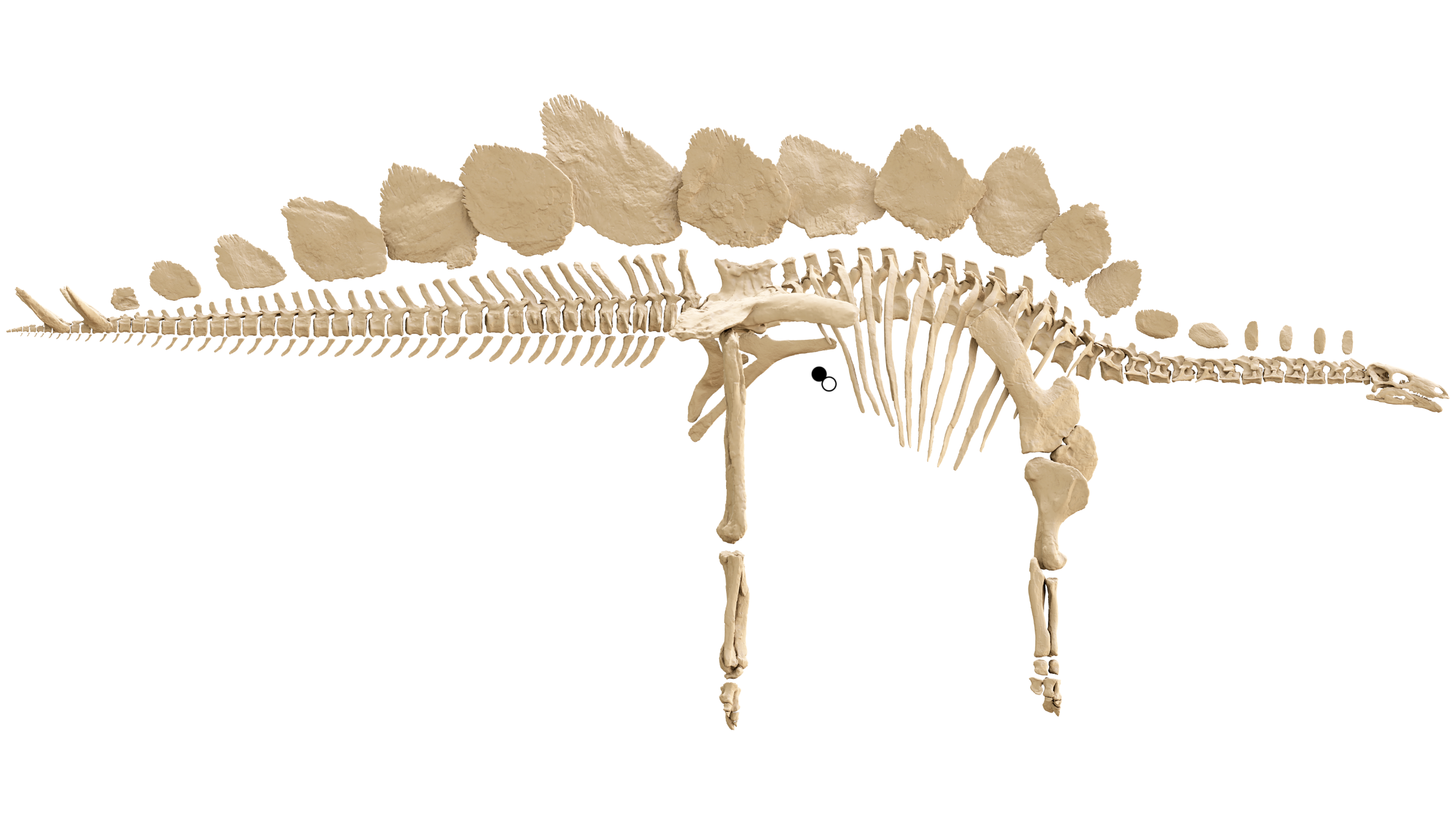


**Fig. S2.** Whole-body centre of mass in *Stegosaurus* in the preferred isometric model variant, with major osteoderms included (black circle) and excluded (white circle).


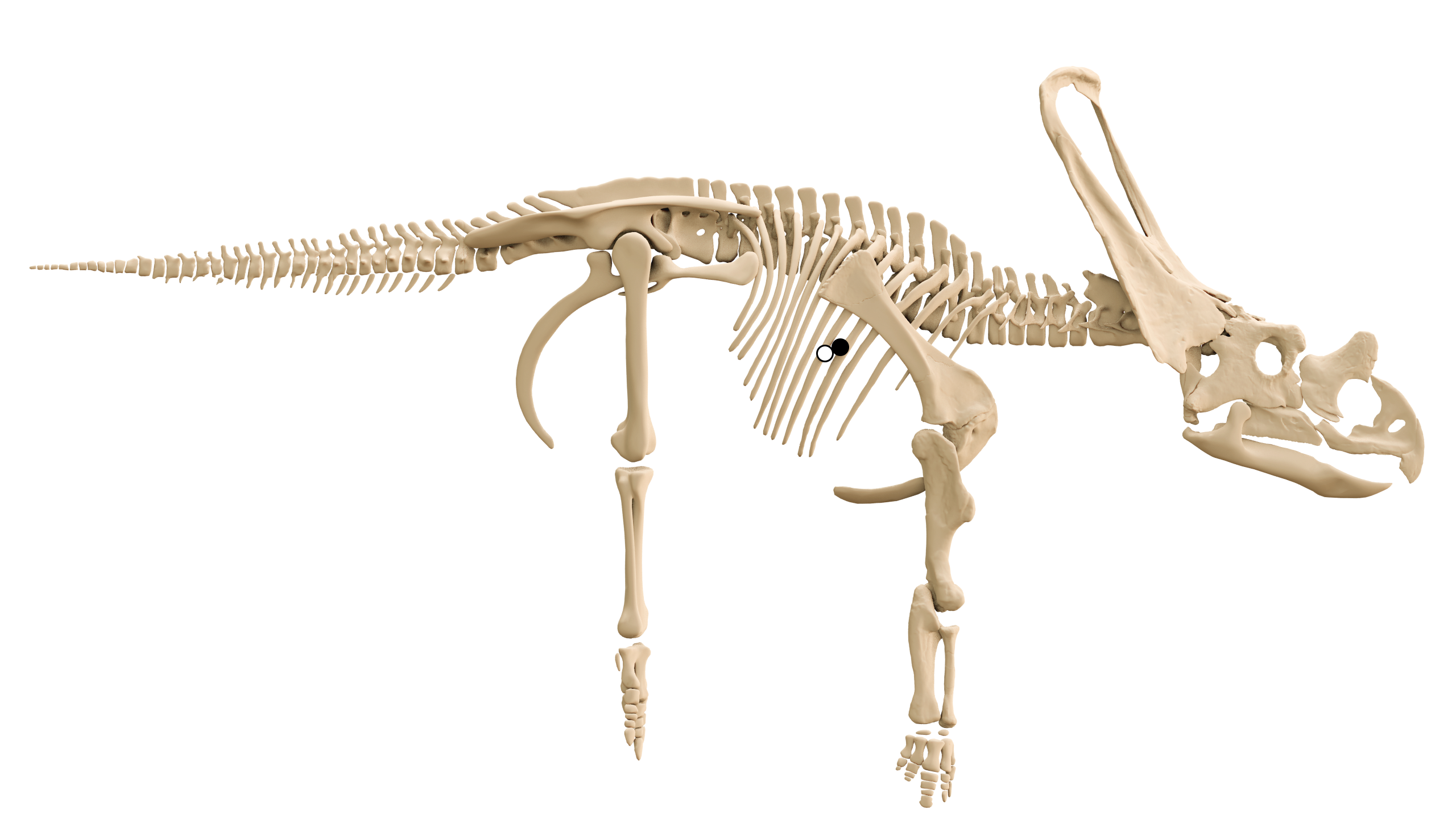


**Fig. S3.** Whole-body centre of mass in *Chasmosaurus* in the preferred isometric model variant, with cranial ornamentation at 2000 kg/m^3^ (black circle) and 1000 kg/m^3^ (white circle).


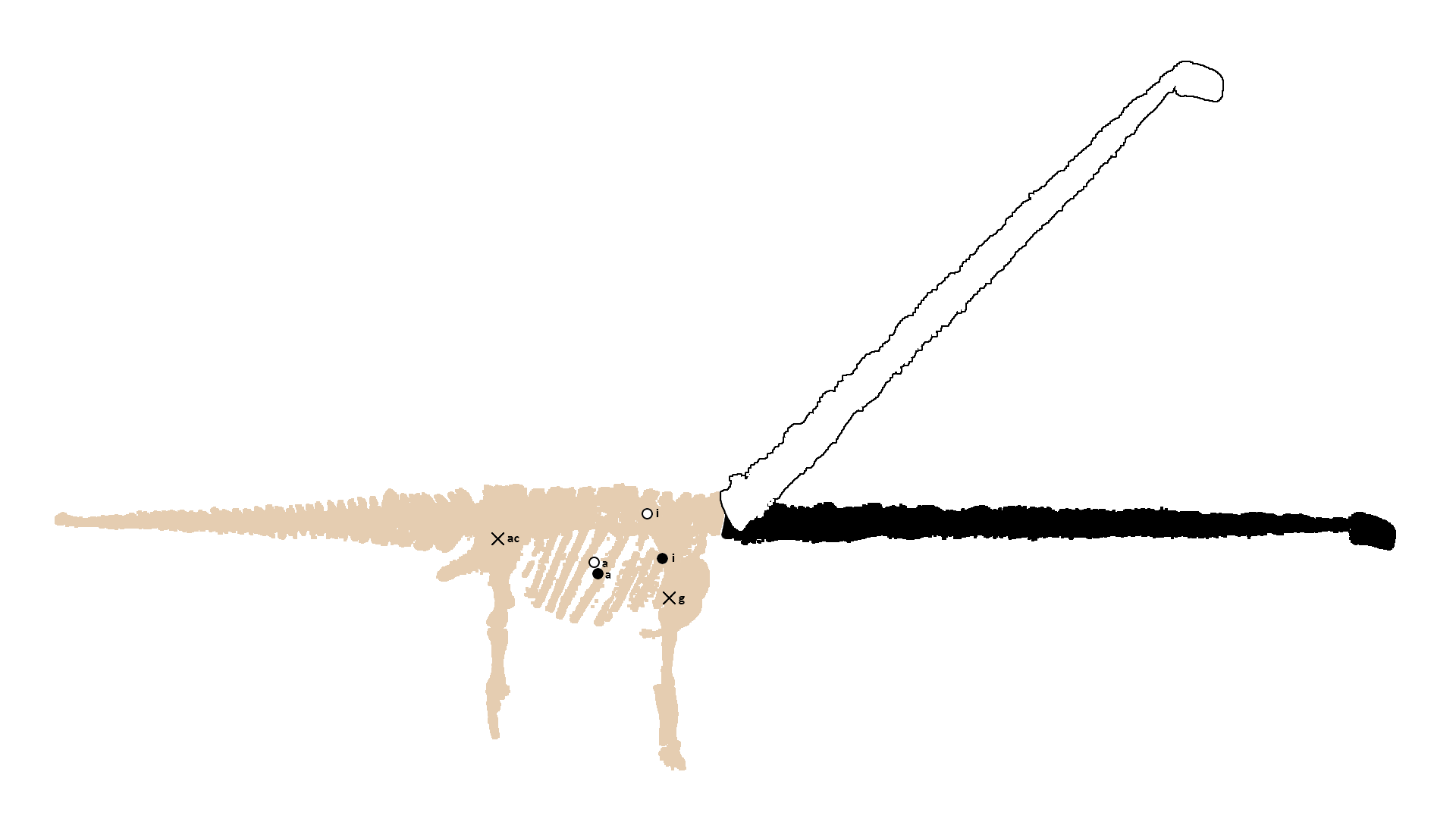


**Fig. S4.** Whole-body centres of mass in *Omeisaurus* with the neck in the reference pose (black circle) and the neck pitched at 45° (white circle). a = preferred allometric model; i = preferred isometric model; X ac = acetabulum; X g = glenoid.

**
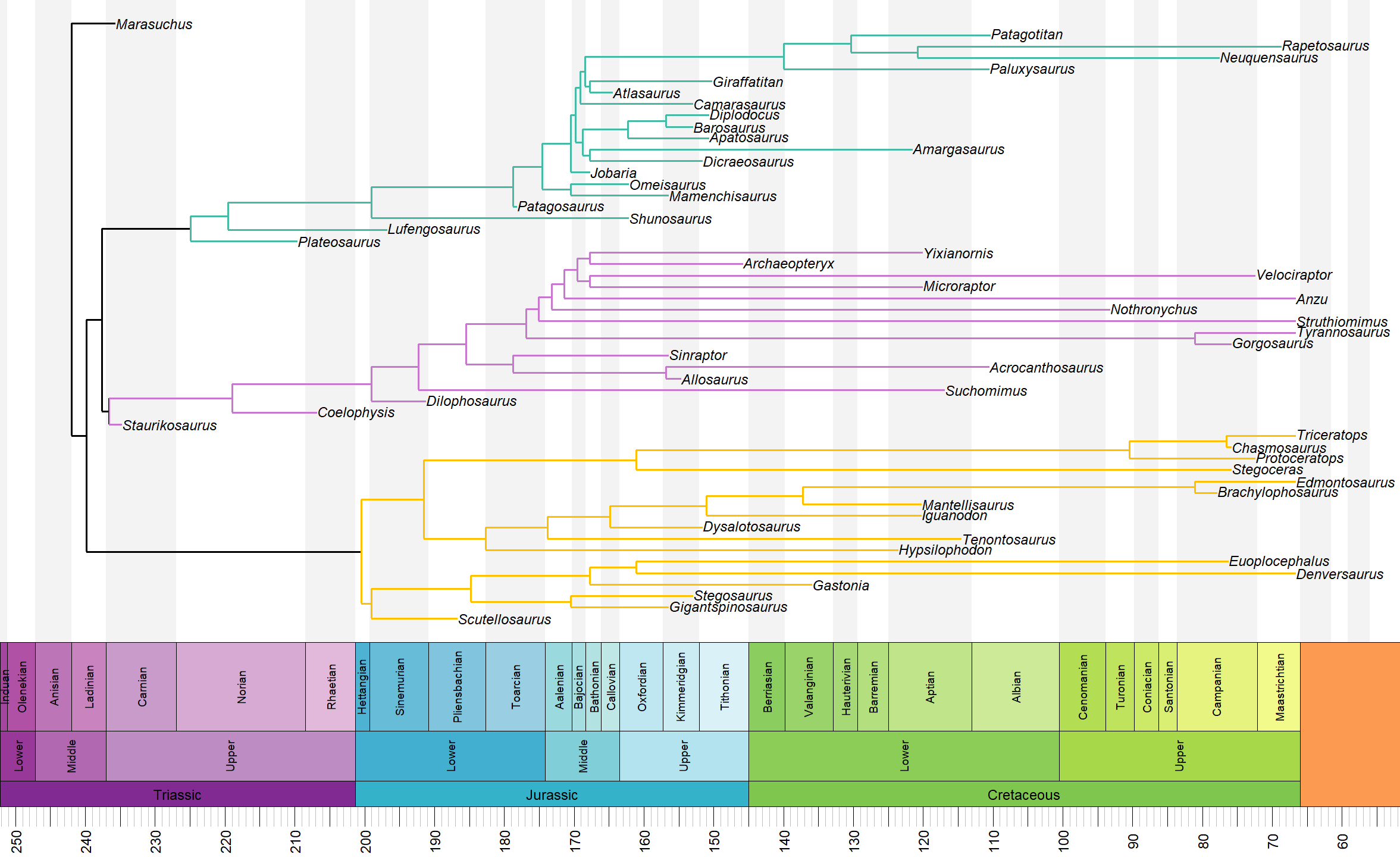
**

**Fig. S5.** Time-calibrated phylogenetic tree of the taxa modelled in this study.

**Table S2.** (see separate file, Table_S2.xlsx). Complete body segment length, volume, and centre of mass data for each primary model set.

**Appendix S2.** (see separate file, Appendix_S2.nex). Phylogenetic tree used in analyses.

**Table S3.** (see separate file, Table_S3.xlsx). Node dates used to construct the phylogenetic tree, and centre of mass ancestral state reconstructions.

**Table S4.** Total body masses for each model variant, using the preferred density approach. Allometric model ranges calculated based on the mean absolute percentage prediction error (mPPE) of each body segment. NAS = non-avian sauropsid.

| Taxon | Preferred allometric model mass (kg) | NAS allometric model mass (kg) | Bird allometric model mass (kg) | Preferred isometric model mass (kg) | NAS isometric model mass (kg) | Bird isometric model mass (kg) |
| --- | --- | --- | --- | --- | --- | --- |
| *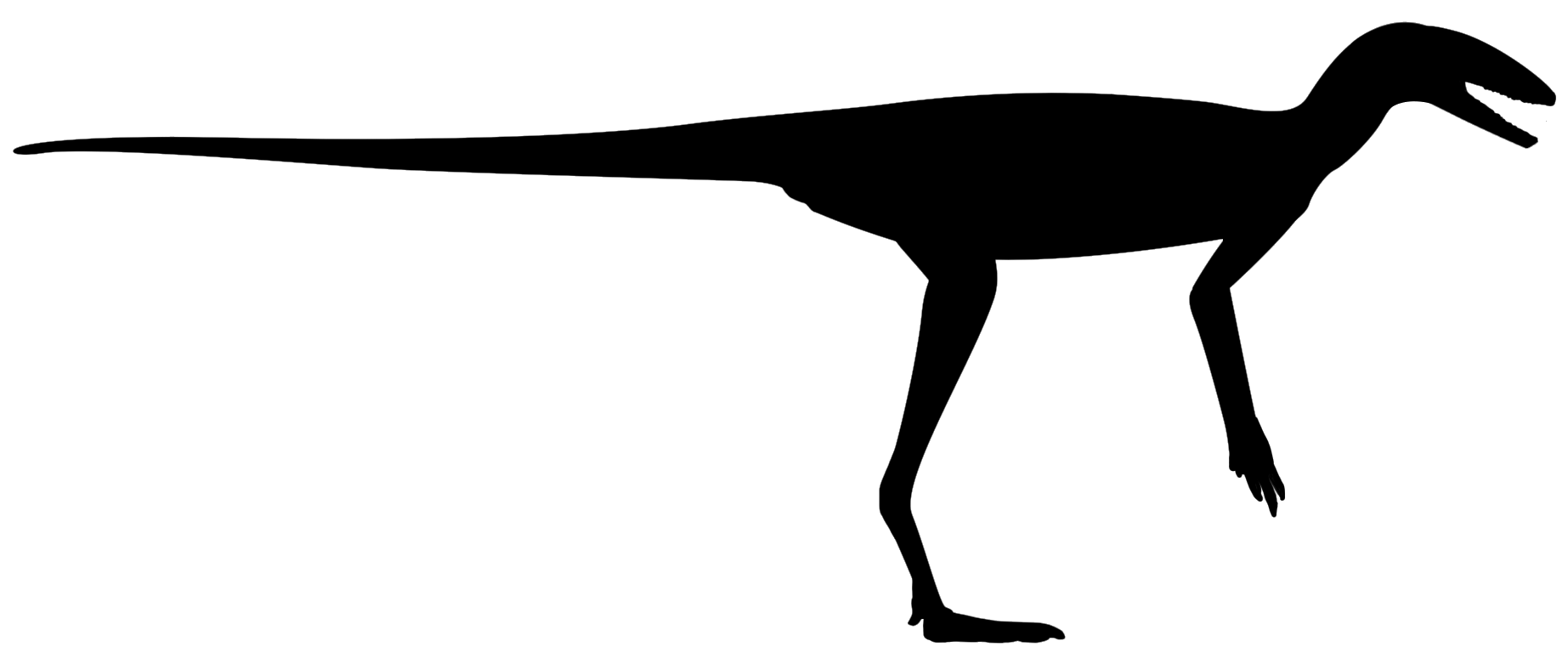*  *Marasuchus* | 0.232  (0.185–0.278) | 0.255  (0.204–0.307) | 0.246  (0.186–0.306) | 0.252 | 0.279 | 0.258 |
| 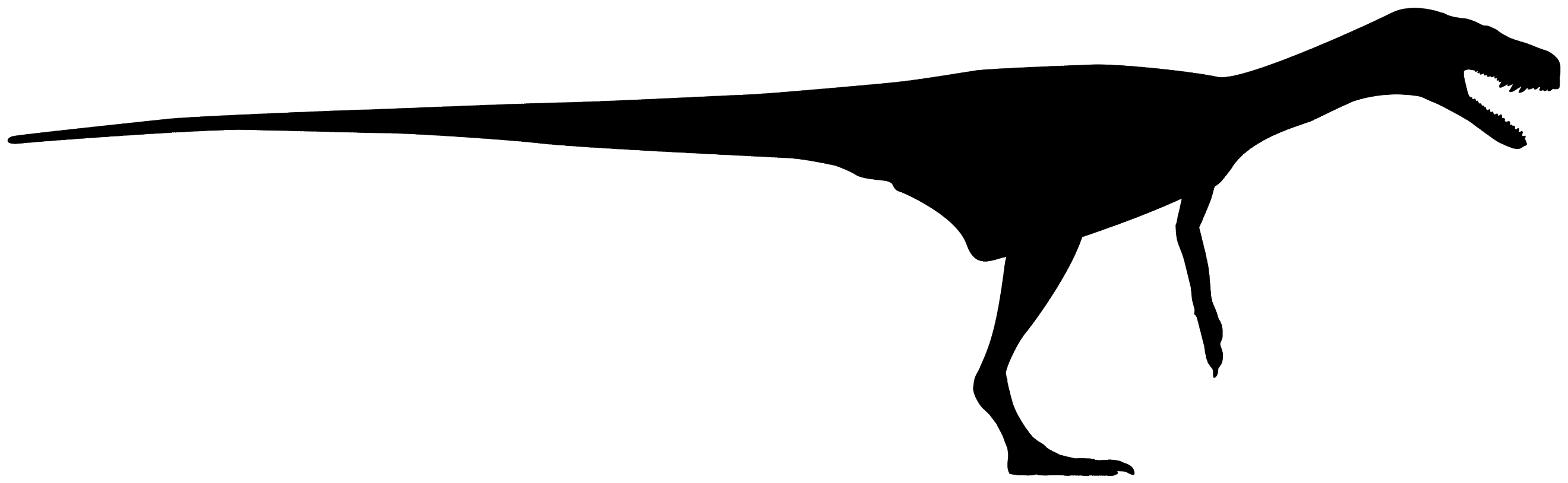*Staurikosaurus* | 22.4  (18.1–26.6) | 26.5  (21.4–31.6) | 22.0  (17.0–27.0) | 21.9 | 25.0 | 22.9 |
| 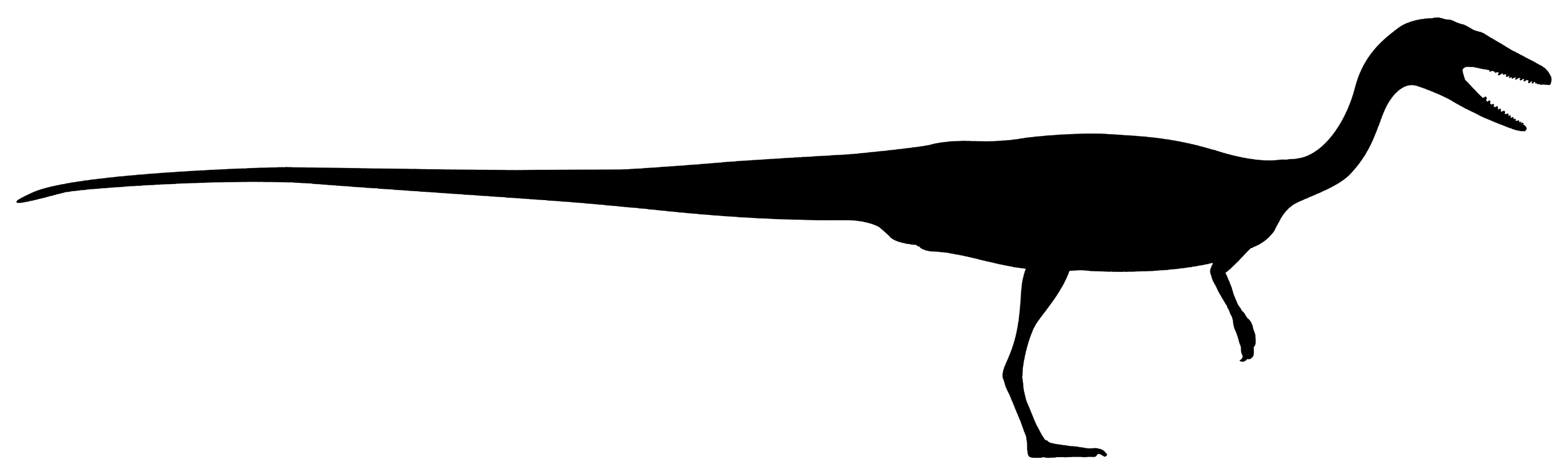*Coelophysis* | 16.2  (12.7–19.8) | 18.1  (14.3–21.9) | 16.9  (12.7–21.0) | 16.6 | 18.2 | 17.7 |
| 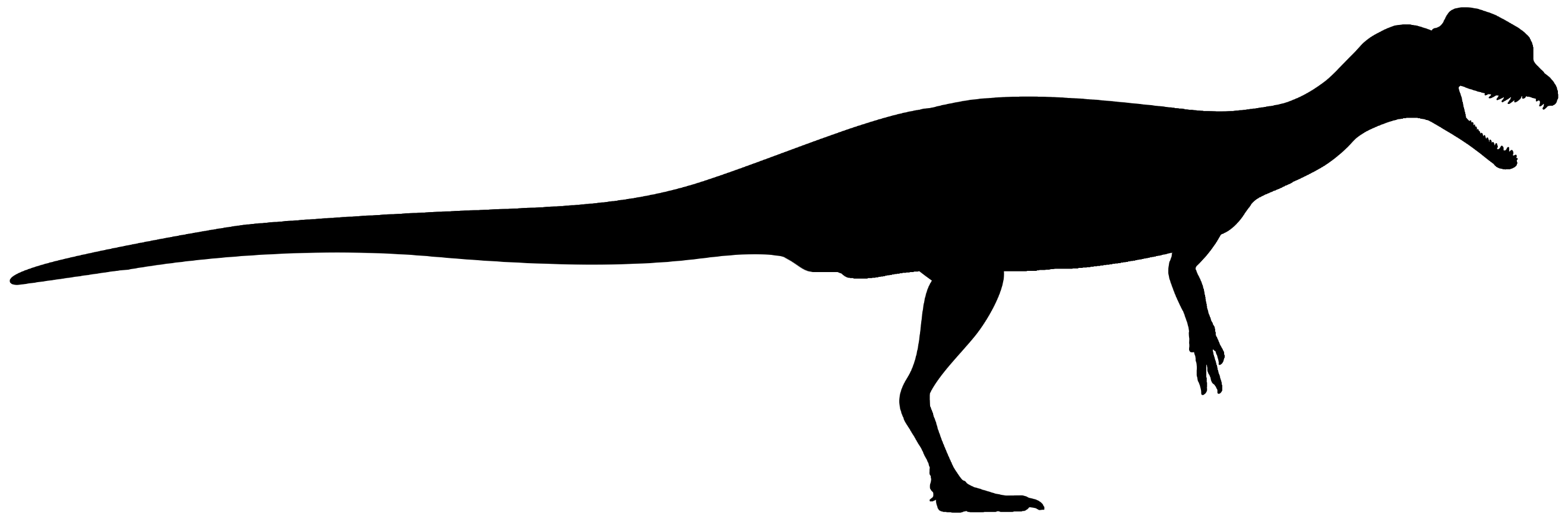*Dilophosaurus* | 555  (460–649) | 631  (521–741) | 533  (425–641) | 494 | 544 | 531 |
| 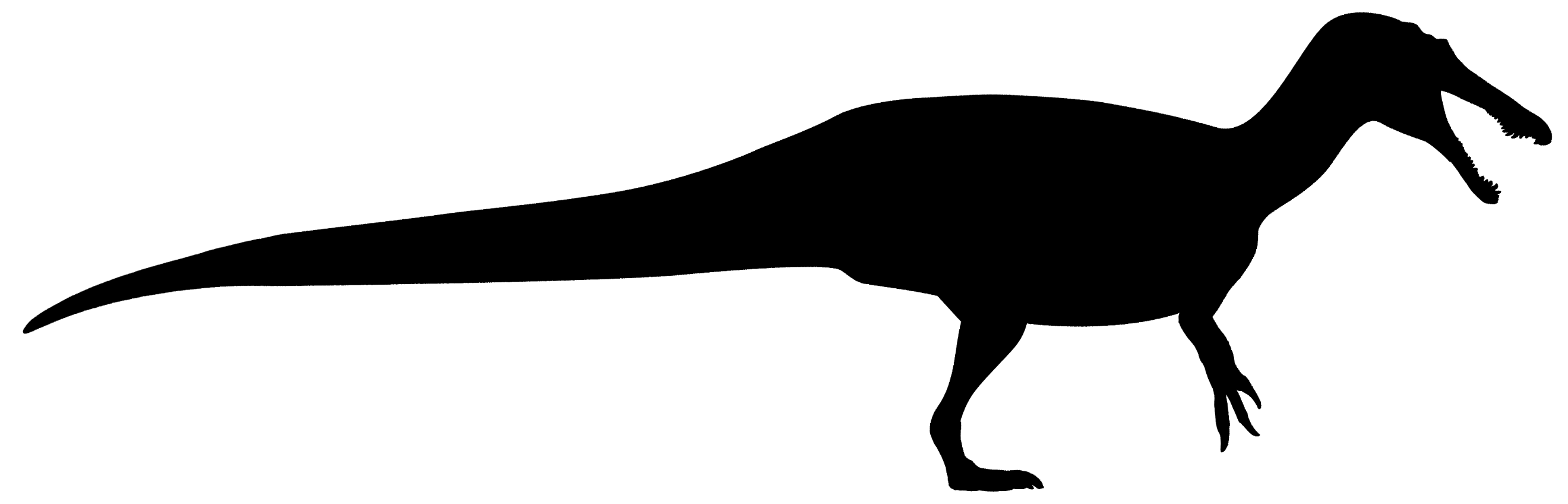*Suchomimus* | 6150  (4999–7314) | 6710  (5429–7992) | 5611  (4372–6871) | 5260 | 5673 | 5652 |
| 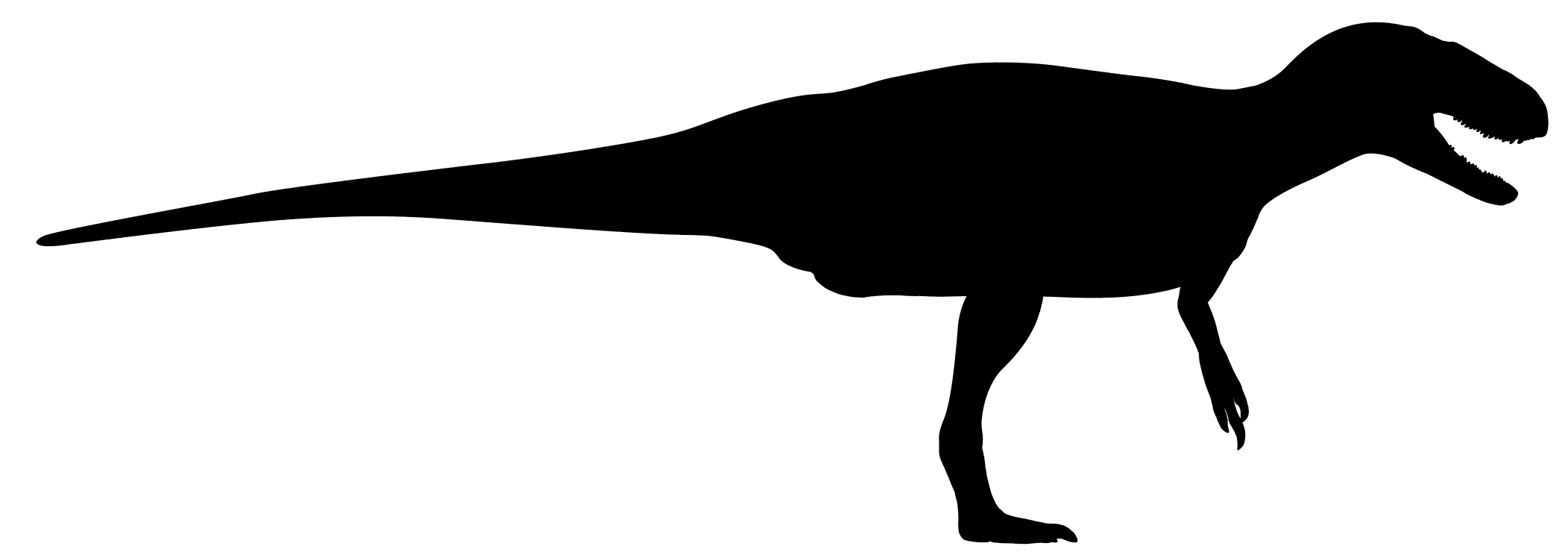  *Sinraptor* | 2676  (2189–3171) | 3253  (2633–3873) | 2398  (1880–2926) | 2361 | 2721 | 2478 |
| 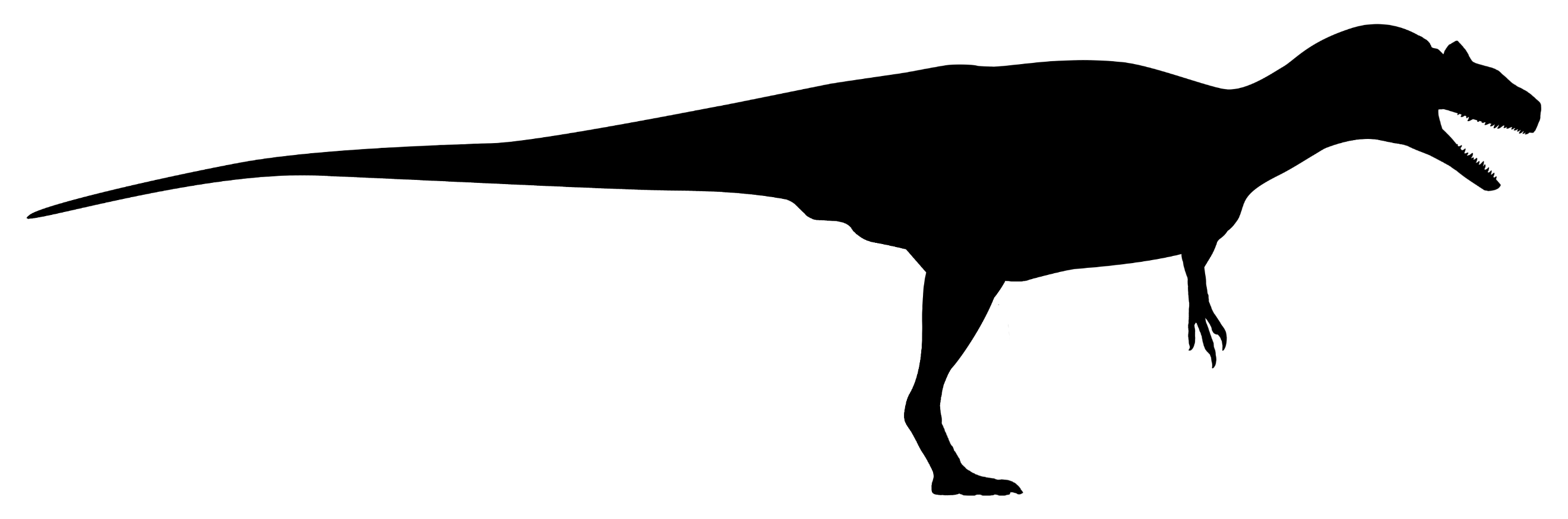  *Allosaurus* | 1282  (1038–1528) | 1488  (1202–1773) | 1172  (901–1445) | 1153 | 1293 | 1221 |
| 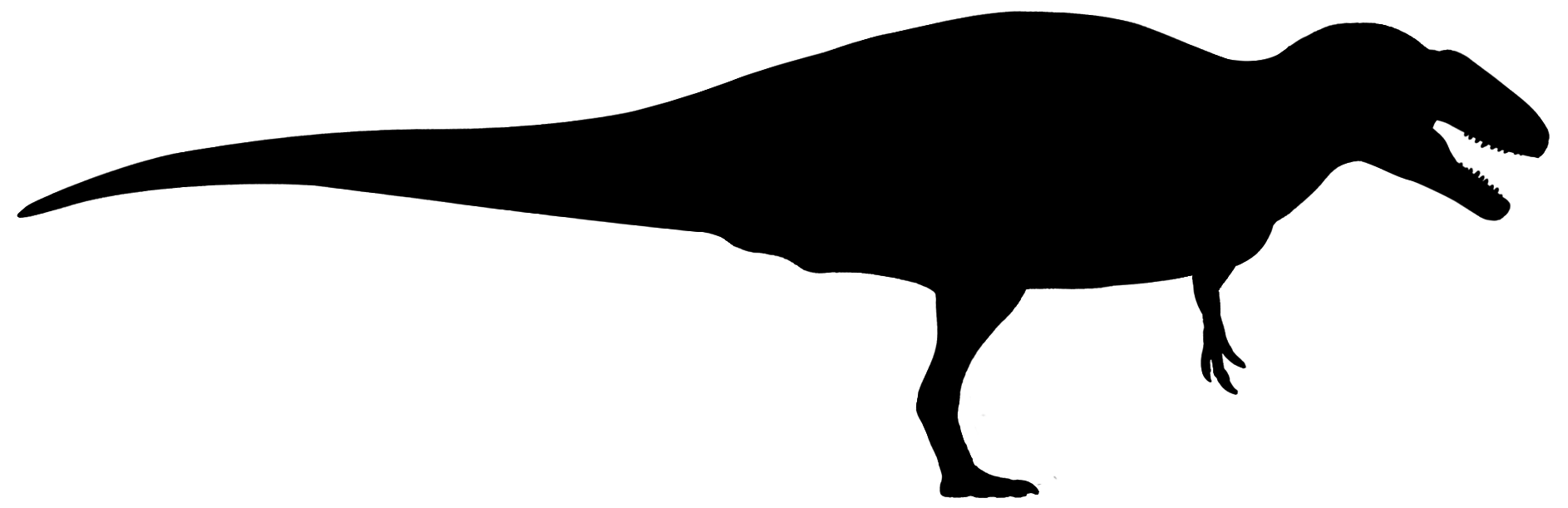  *Acrocanthosaurus* | 9174  (7274–11109) | 10219  (8083–12354) | 8458  (6419–10537) | 8364 | 9153 | 8888 |
| 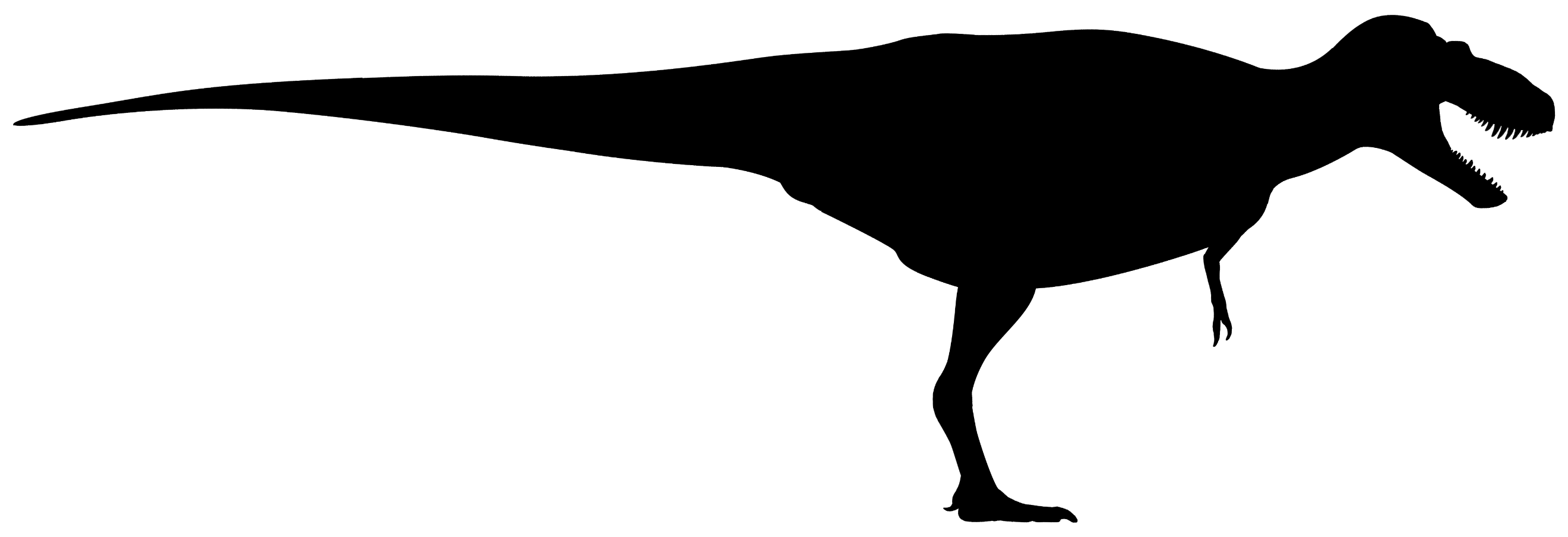  *Gorgosaurus* | 4697  (3928–5473) | 5438  (4505–6370) | 4228  (3367–5098) | 3886 | 4291 | 4177 |
| 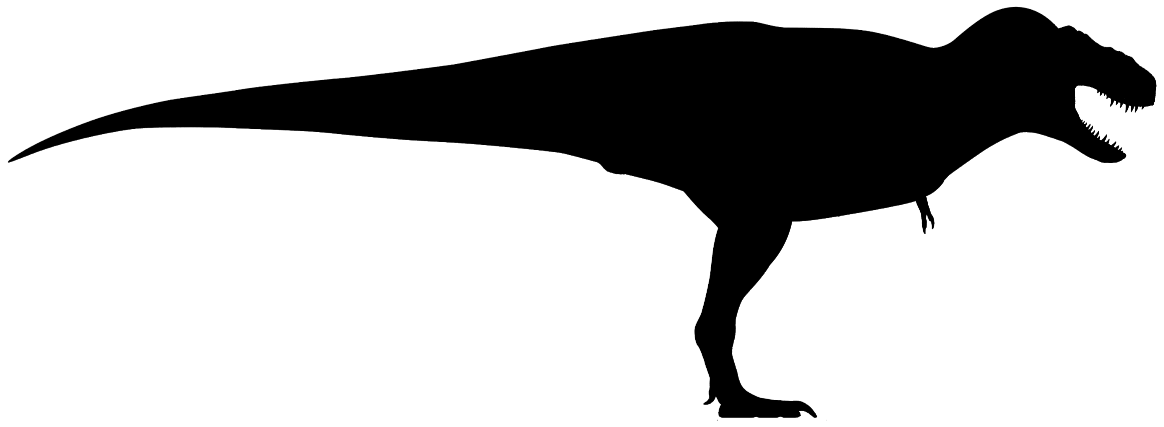  *Tyrannosaurus* | 9193  (7579–10854) | 10919  (8892–12947) | 8024  (6295–9802) | 7926 | 9072 | 8363 |
| 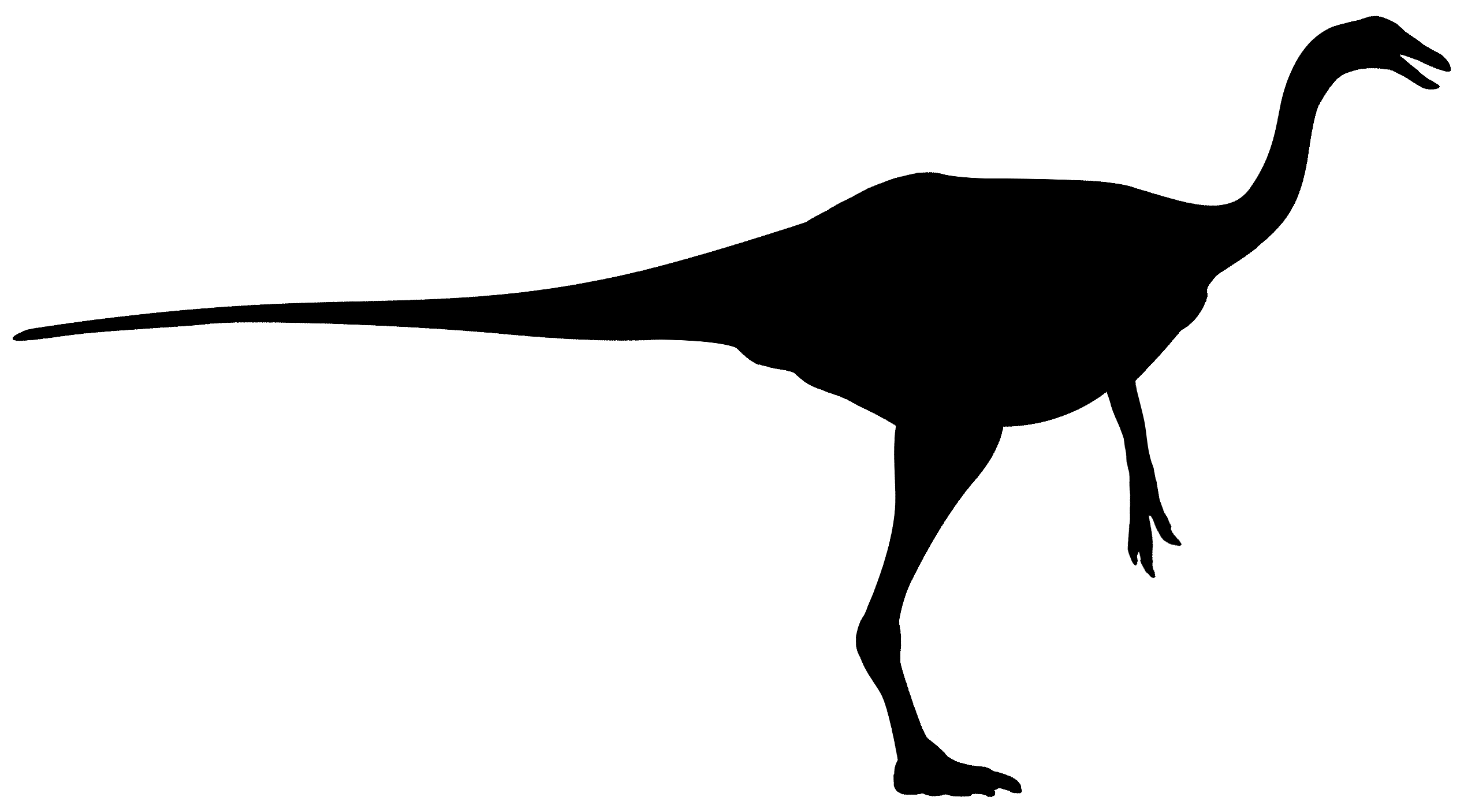  *Struthiomimus* | 592  (484–700) | 690  (559–821) | 518  (398–638) | 506 | 561 | 530 |
| 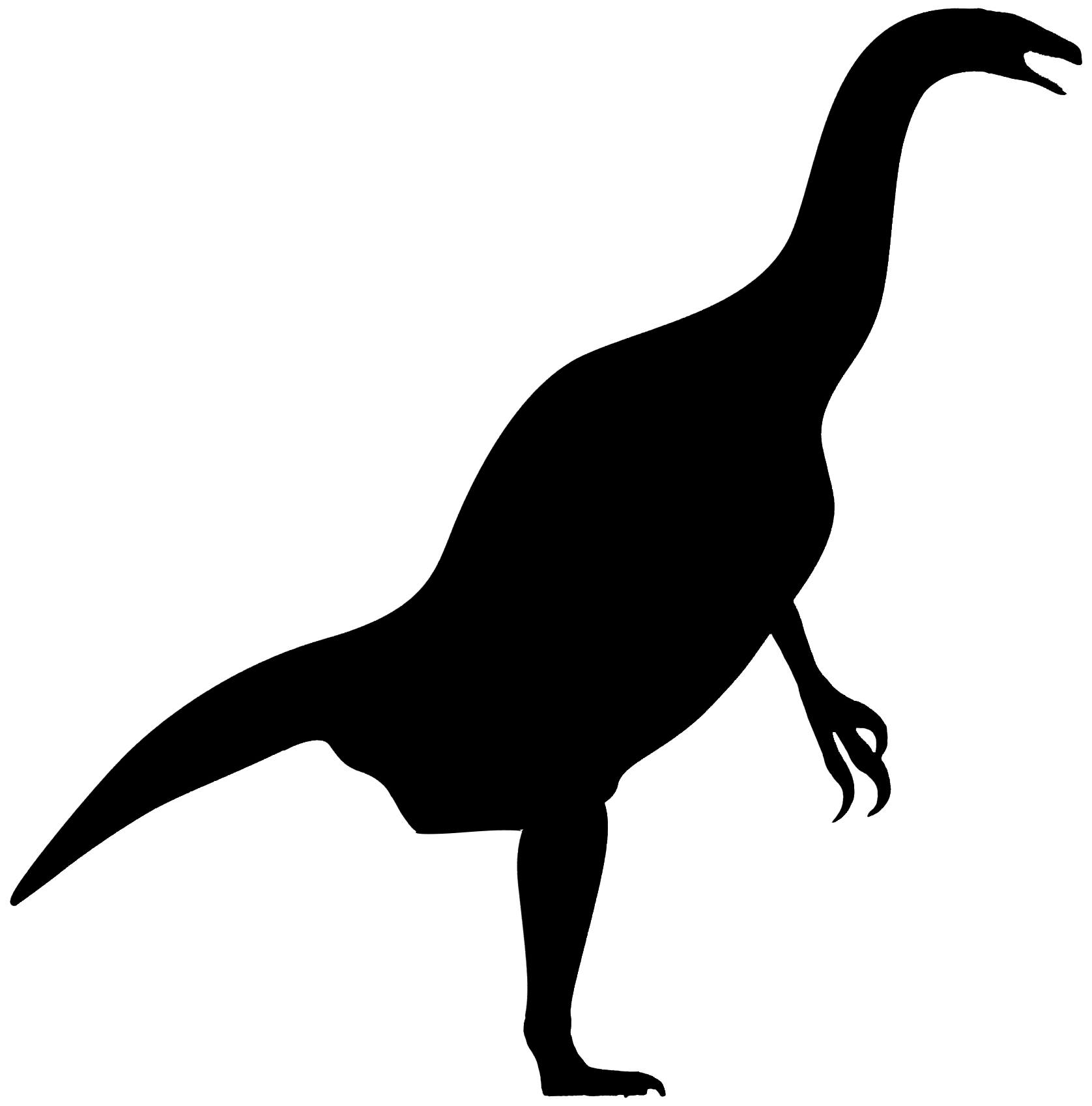  *Nothronychus* | 1056  (903–1211) | 1172  (994–1350) | 999  (815–1184) | 876 | 964 | 966 |
| 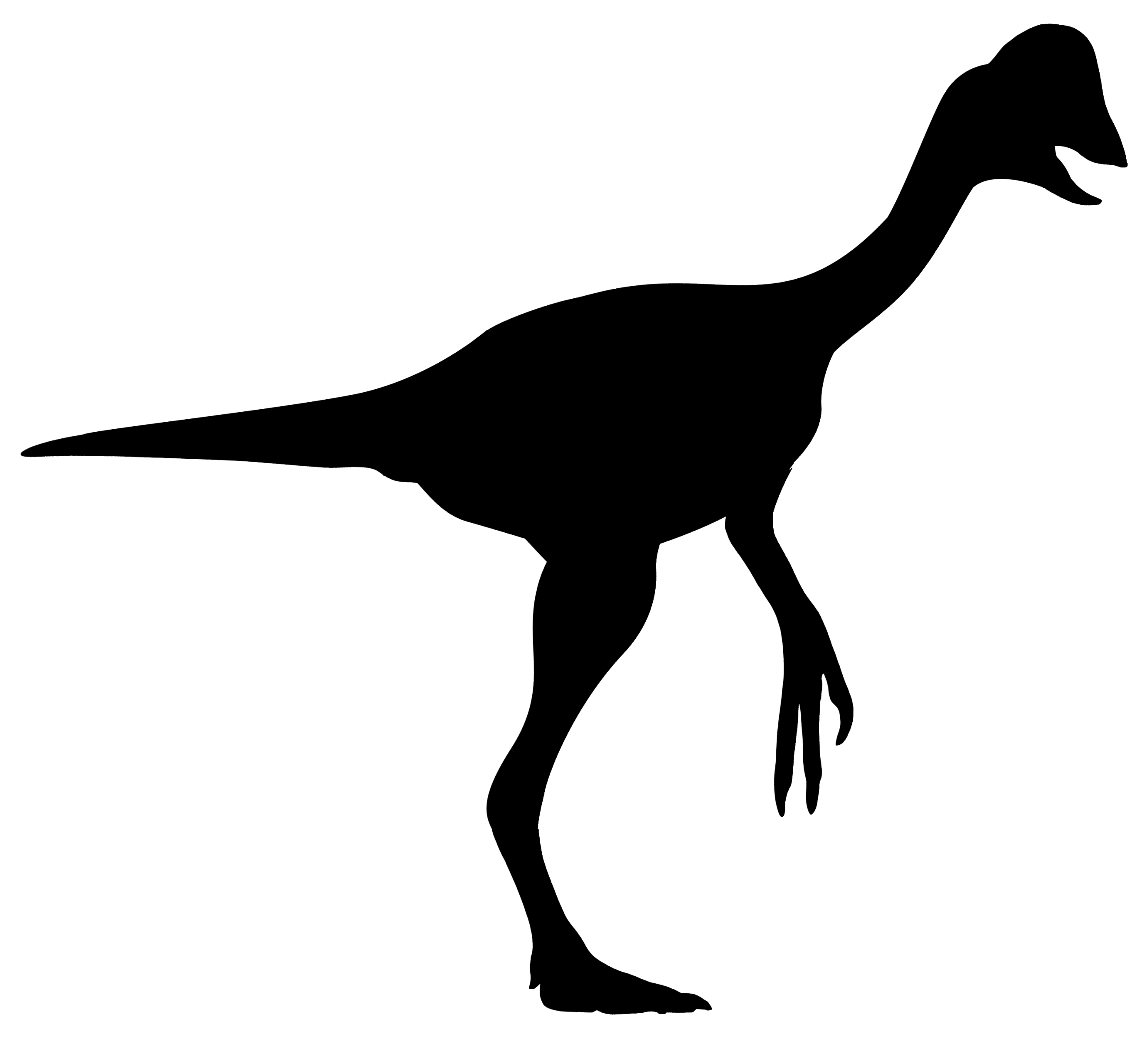  *Anzu* | 338  (279–398) | 442  (359–524) | 307  (237–377) | 302 | 363 | 320 |
| 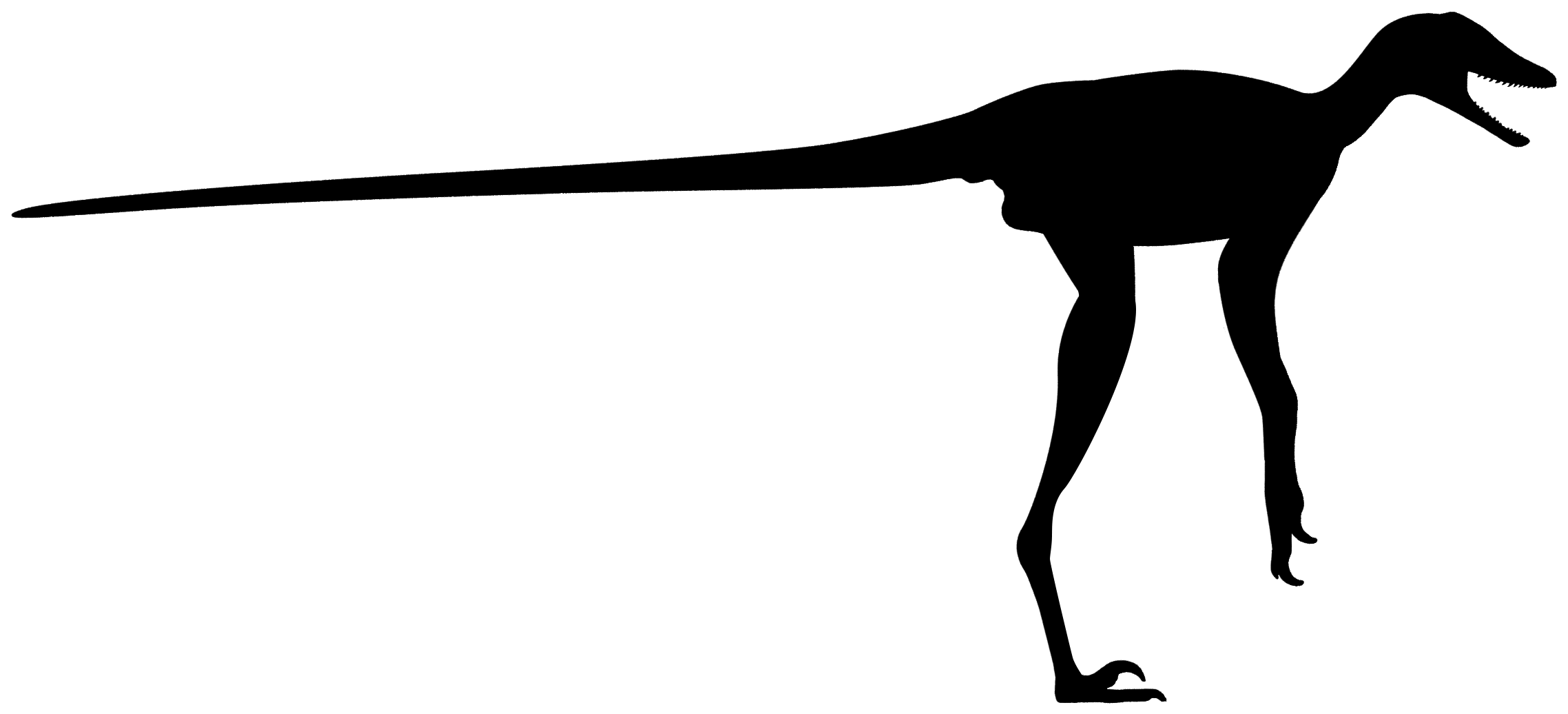  *Microraptor* | 0.885  (0.701–1.07) | 1.053  (0.835–1.27) | 0.949  (0.719–1.18) | 0.935 | 1.094 | 0.996 |
| 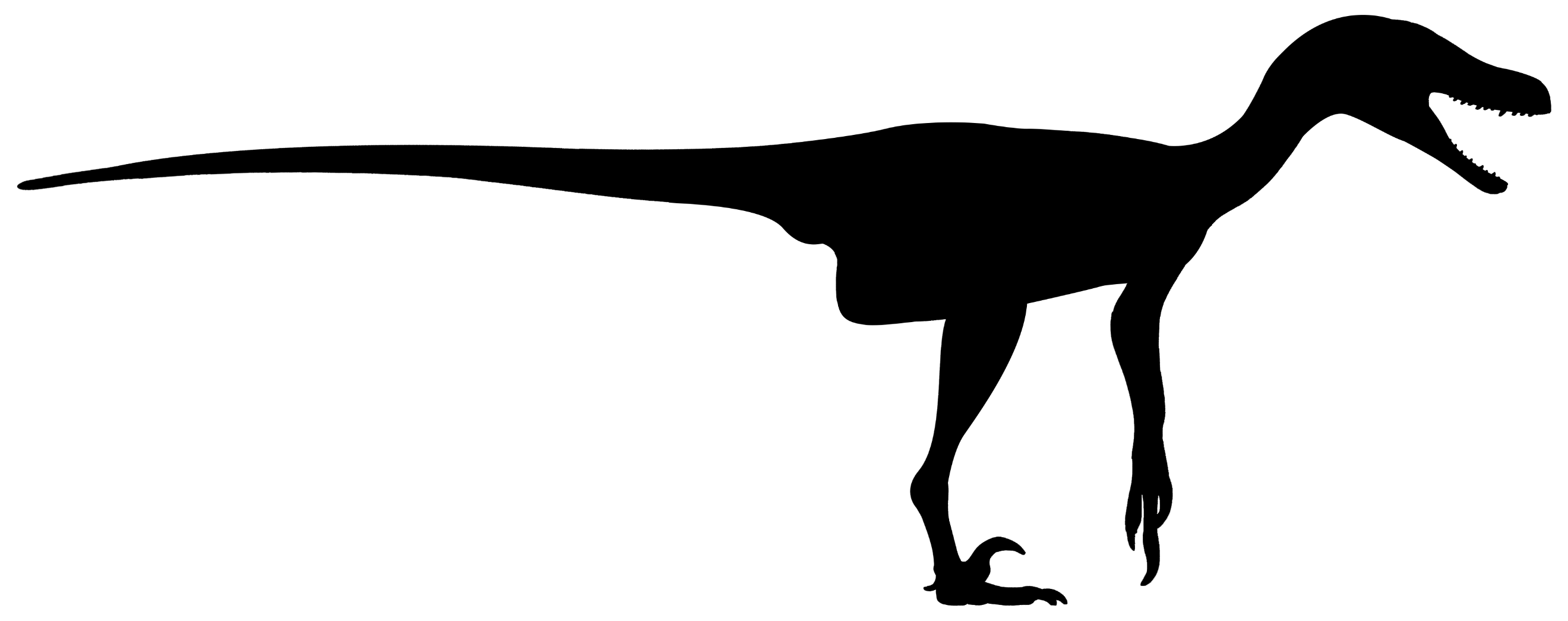  *Velociraptor* | 12.7  (9.93–15.4) | 15.4  (12.2–18.6) | 12.5  (9.30–15.7) | 12.8 | 14.9 | 13.4 |
| 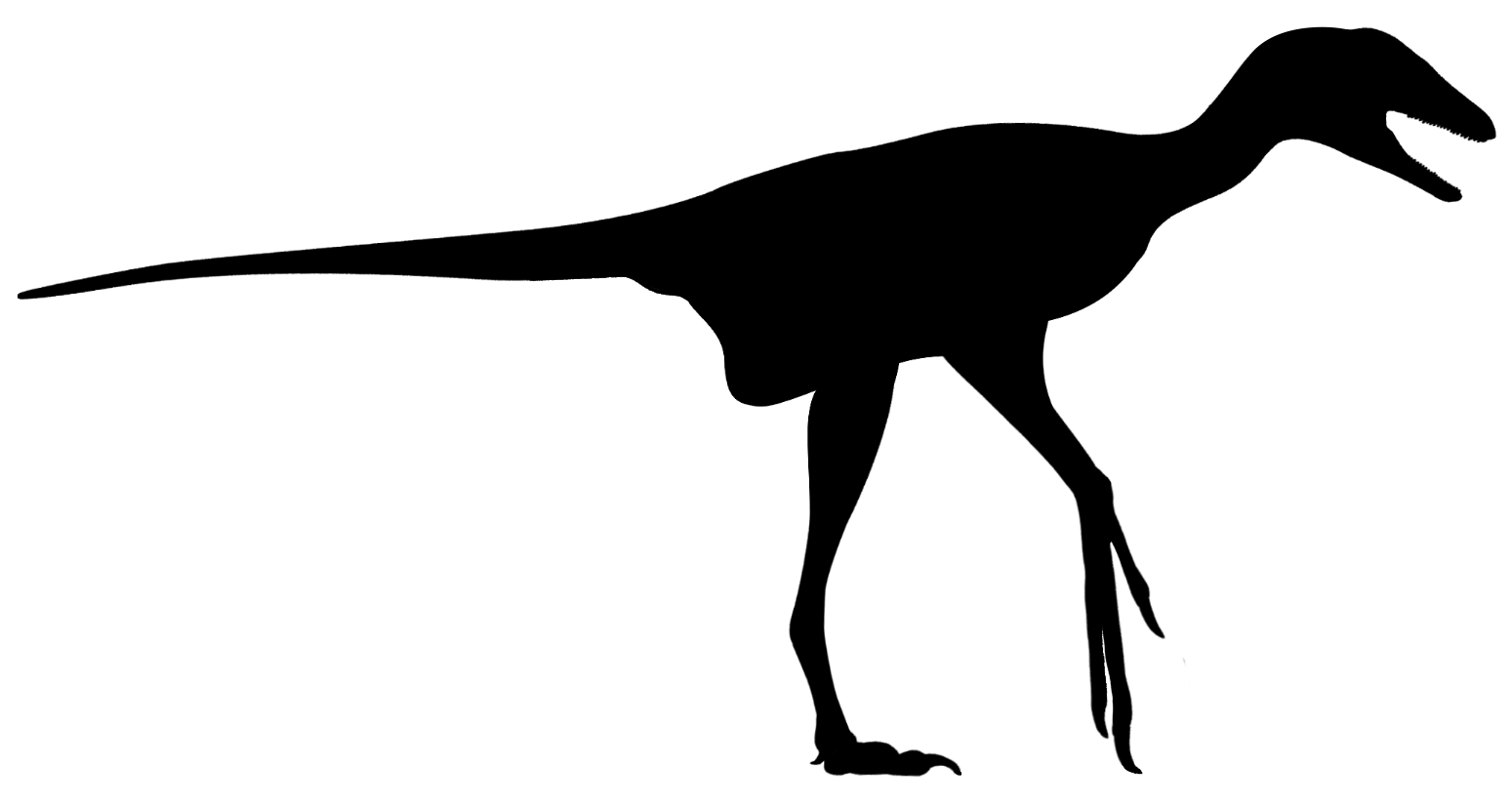  *Archaeopteryx* | 0.189  (0.142–0.237) | 0.228  (0.183–0.274) | 0.206  (0.155–0.258) | 0.204 | 0.249 | 0.216 |
| 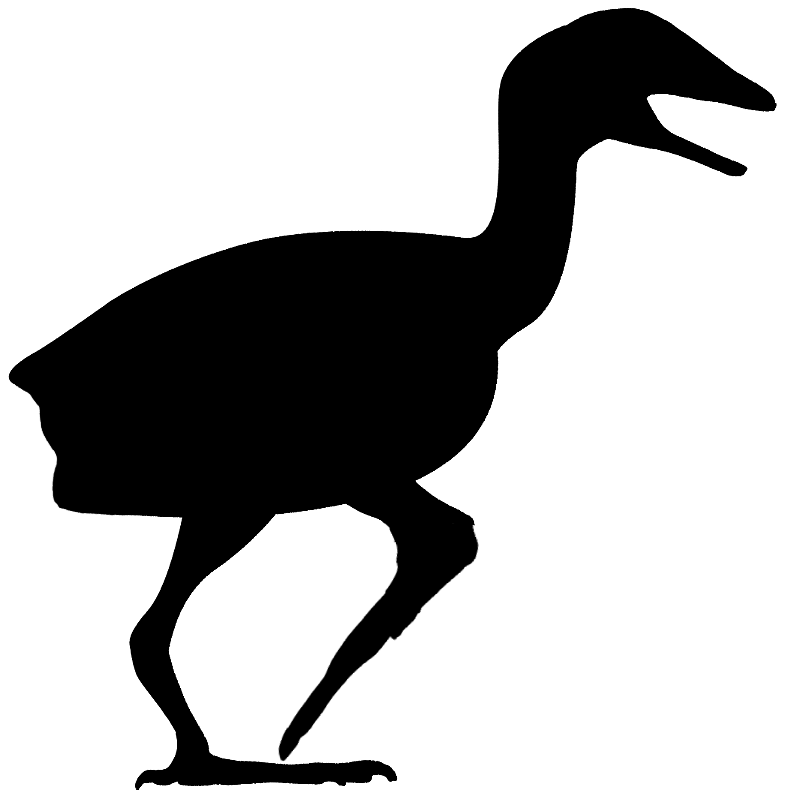  *Yixianornis* | 0.135  (0.108–0.162) | 0.136  (0.114–0.159) | 0.135  (0.108–0.162) | 0.140 | 0.150 | 0.140 |
| 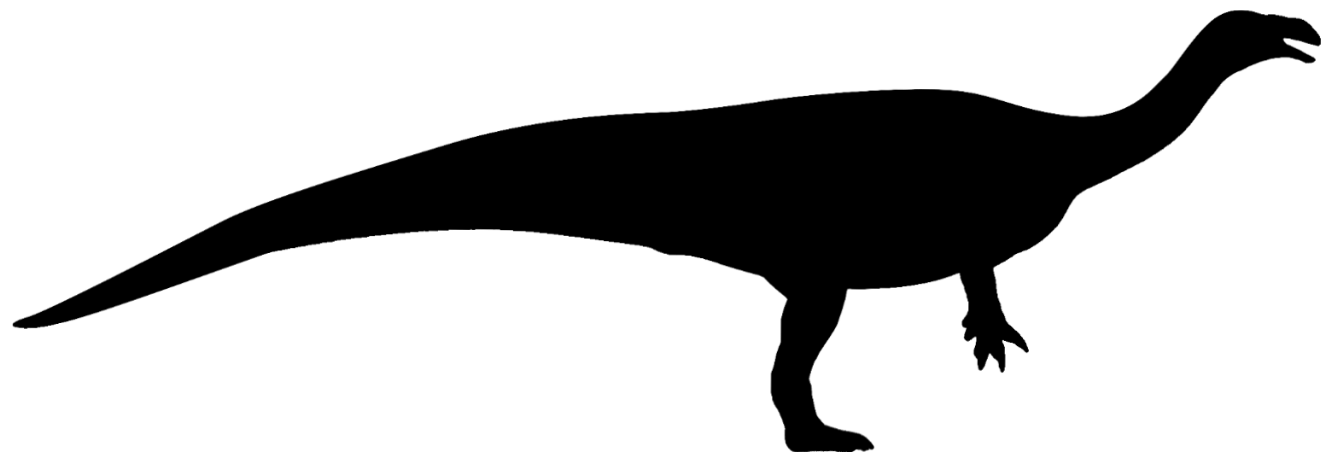  *Plateosaurus* | 769  (626–913) | 853  (693–1013) | 719  (558–881) | 676 | 729 | 727 |
| 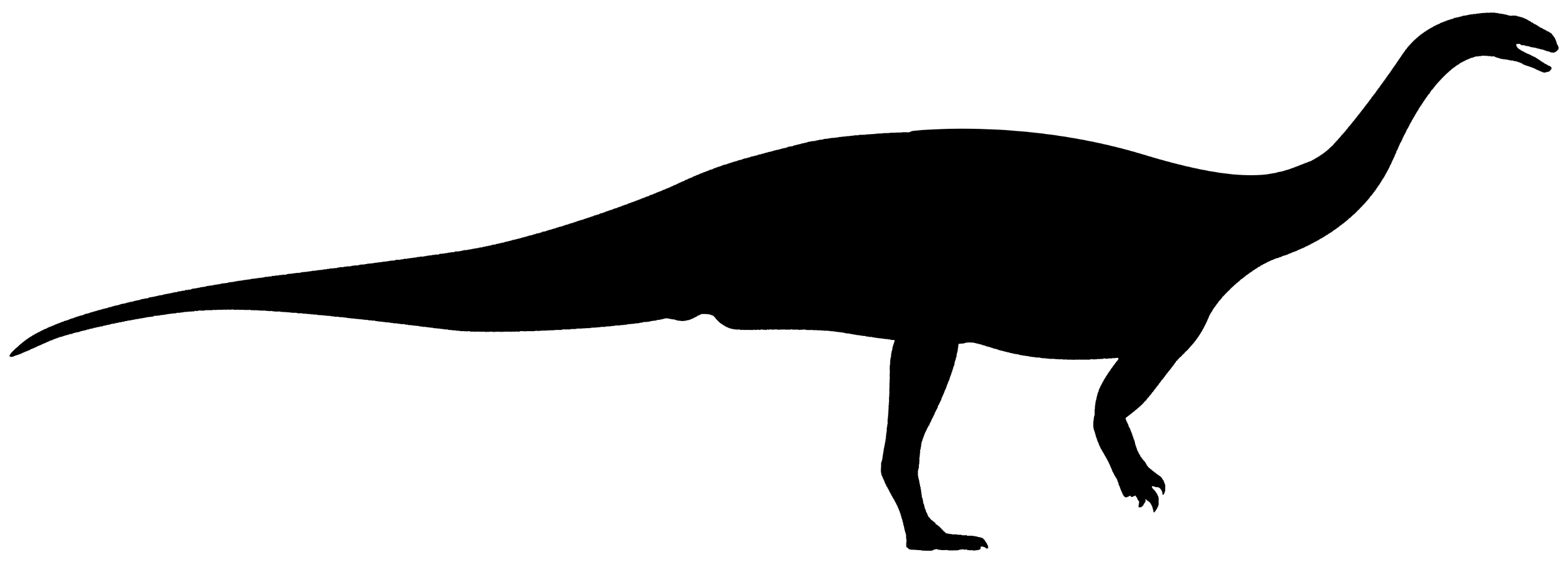  *Lufengosaurus* | 797  (648–947) | 902  (733–1072) | 741  (576–909) | 706 | 781 | 757 |
| 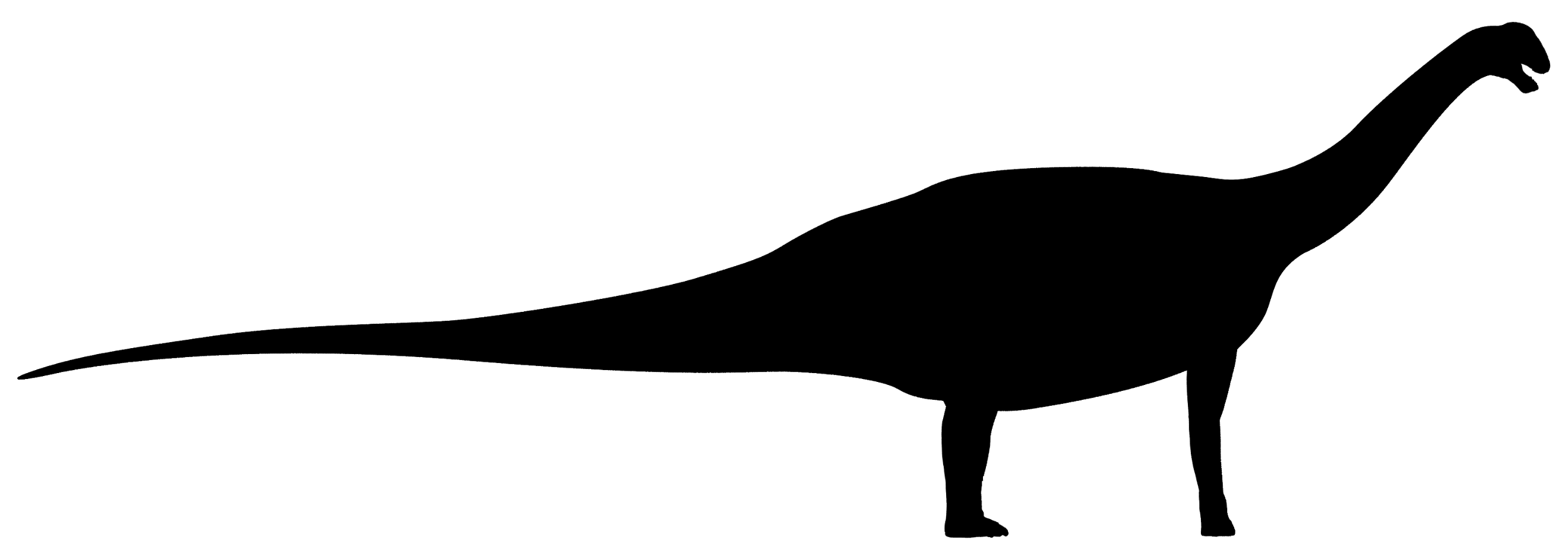  *Patagosaurus* | 16040  (13040–19094) | 16833  (13645–20021) | 12192  (9758–15300) | 12315 | 13165 | 12665 |
| 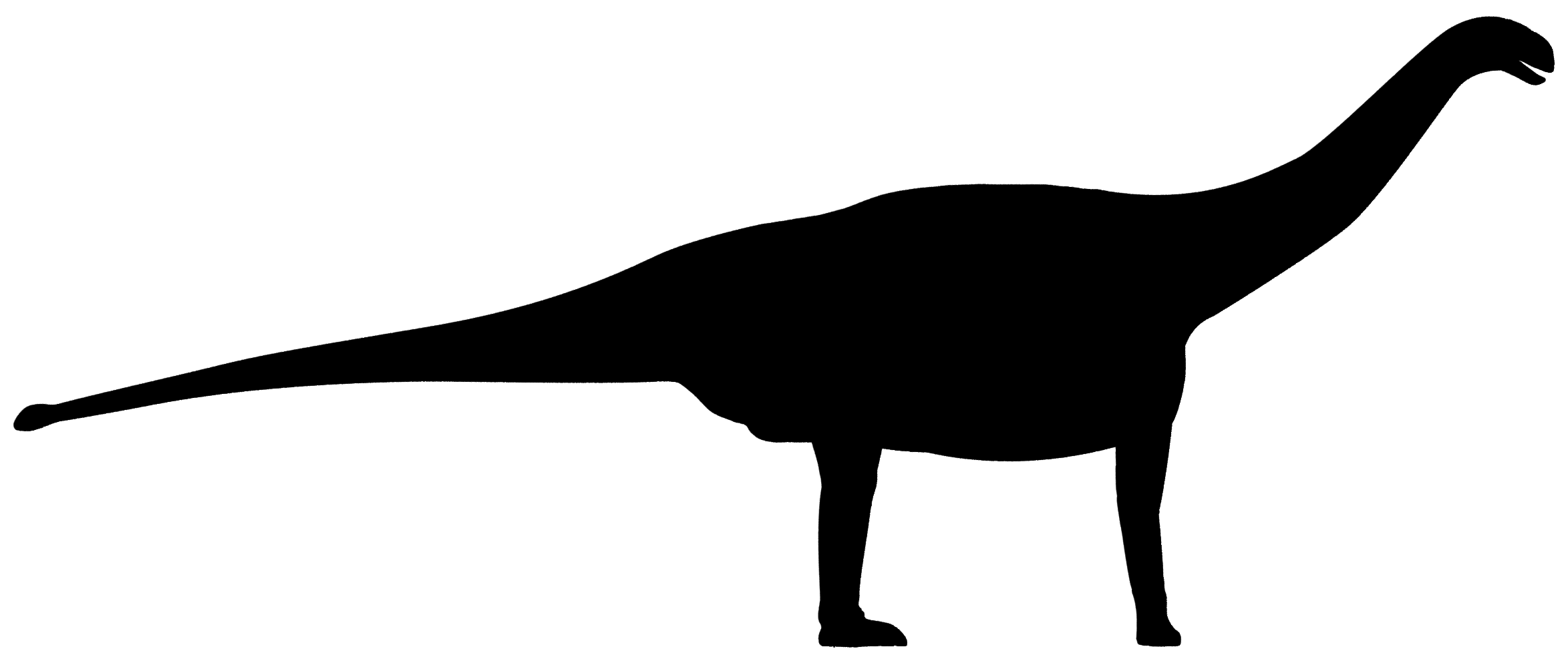  *Shunosaurus* | 3790  (3118–4474) | 4045  (3320–4770) | 3171  (2519–3847) | 3064 | 3332 | 3198 |
| 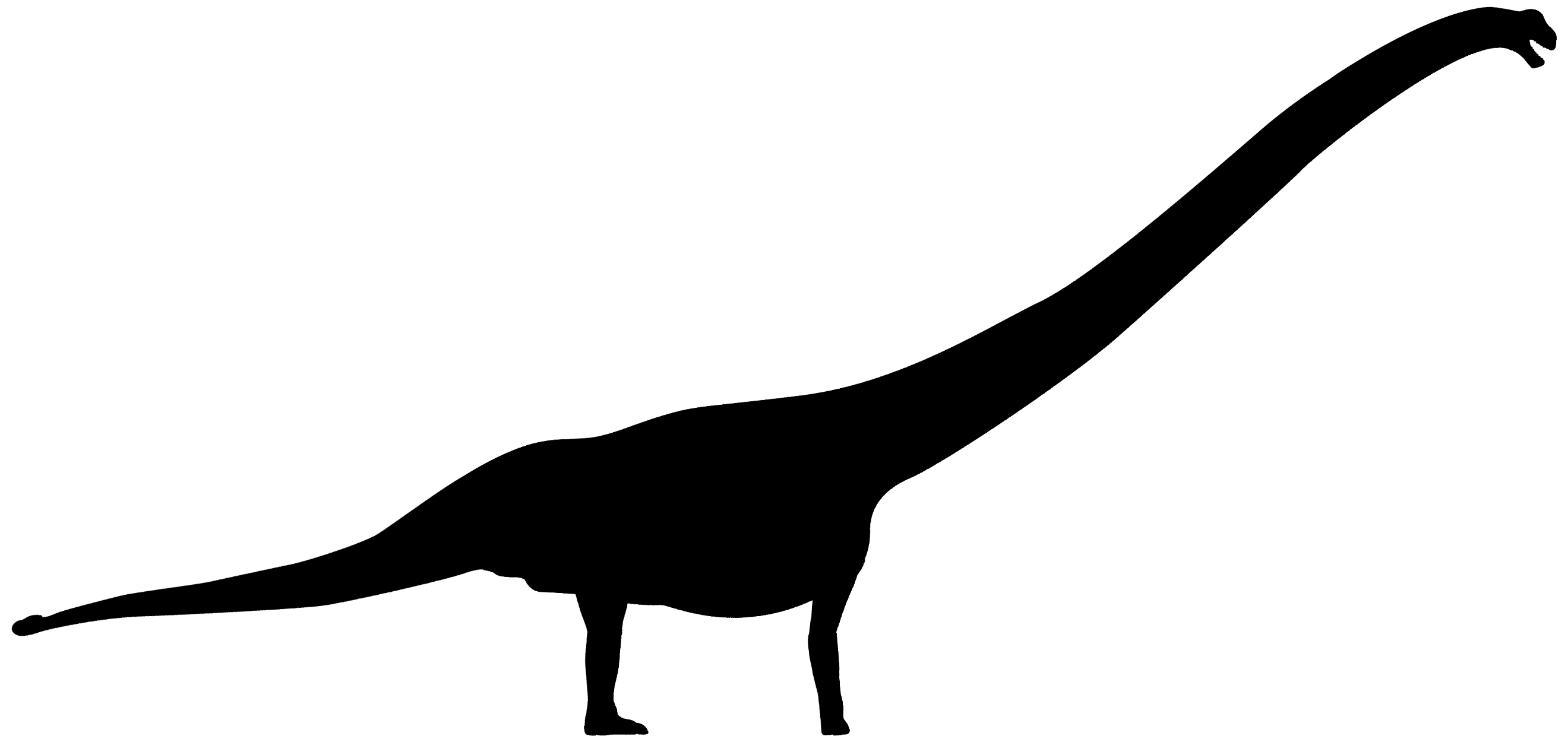  *Mamenchisaurus* | 14817  (12387–17560) | 18149  (14823–21475) | 12516  (10135–15260) | 13395 | 17082 | 14077 |
| 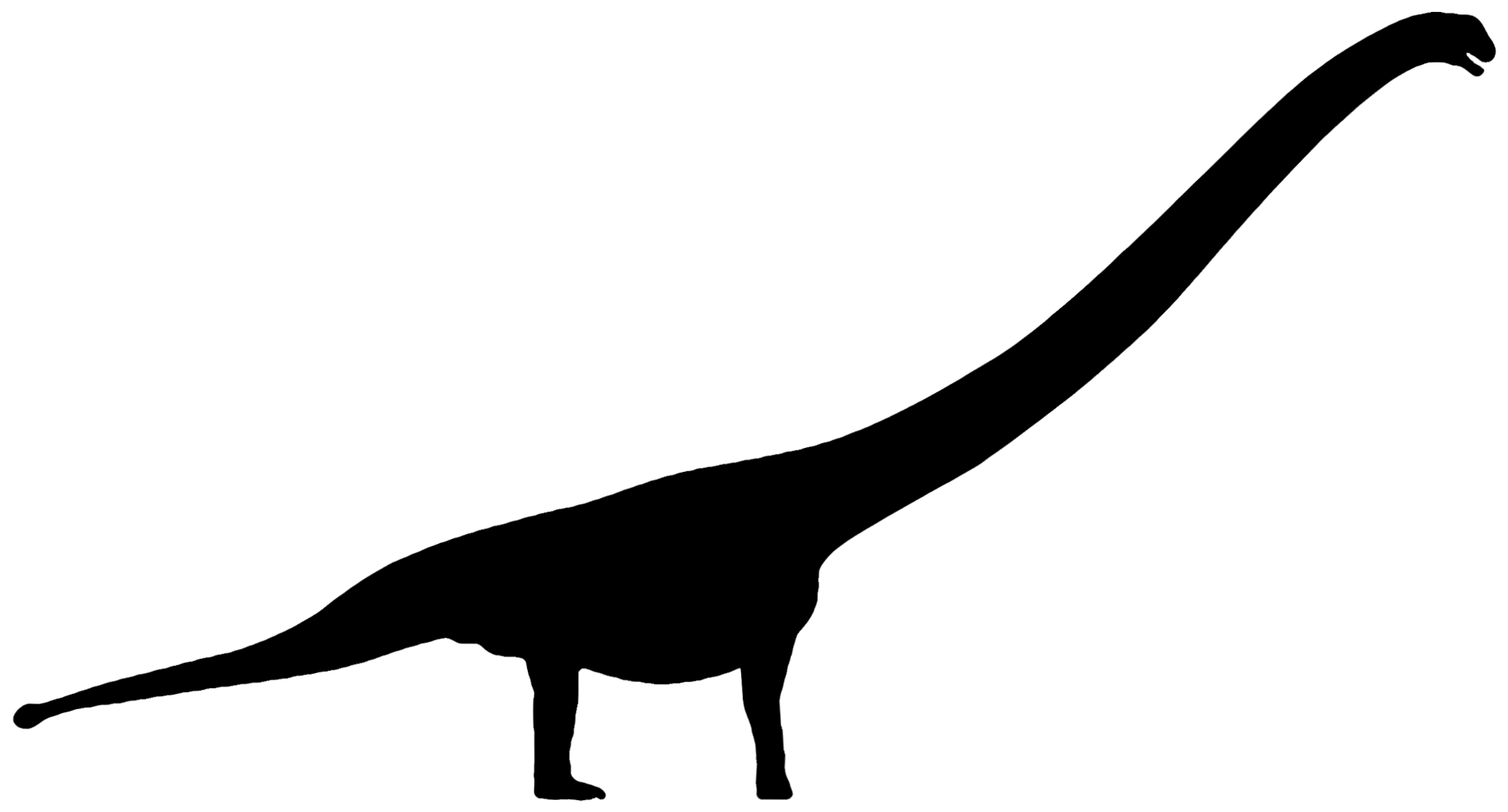  *Omeisaurus* | 14471  (12236–17059) | 18173  (14935–21410) | 12067  (9908–14627) | 13147 | 17254 | 13751 |
| 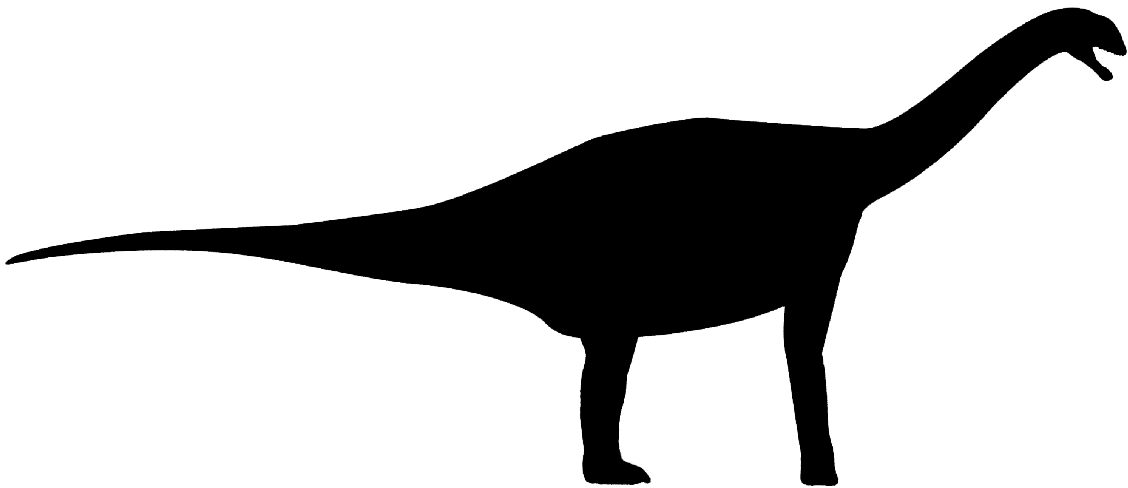  *Jobaria* | 26315  (21677–31202) | 29114  (23734–34494) | 18915  (15221–23040) | 20037 | 23122 | 20162 |
| 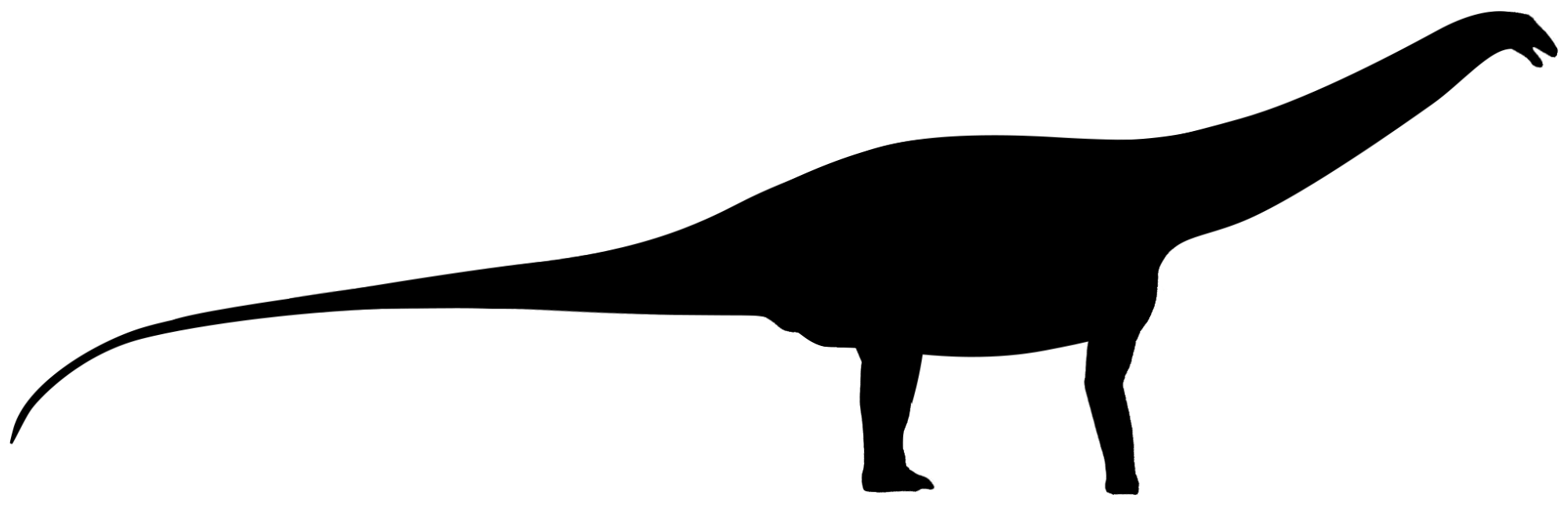*Apatosaurus* | 46428  (39112–54432) | 56415  (46347–66483) | 38005  (30892–45937) | 40446 | 52066 | 42624 |
| 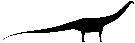  *Diplodocus* | 19842  (16295–23646) | 22667  (18371–26964) | 16941  (13297–20878) | 17293 | 20408 | 18321 |
| 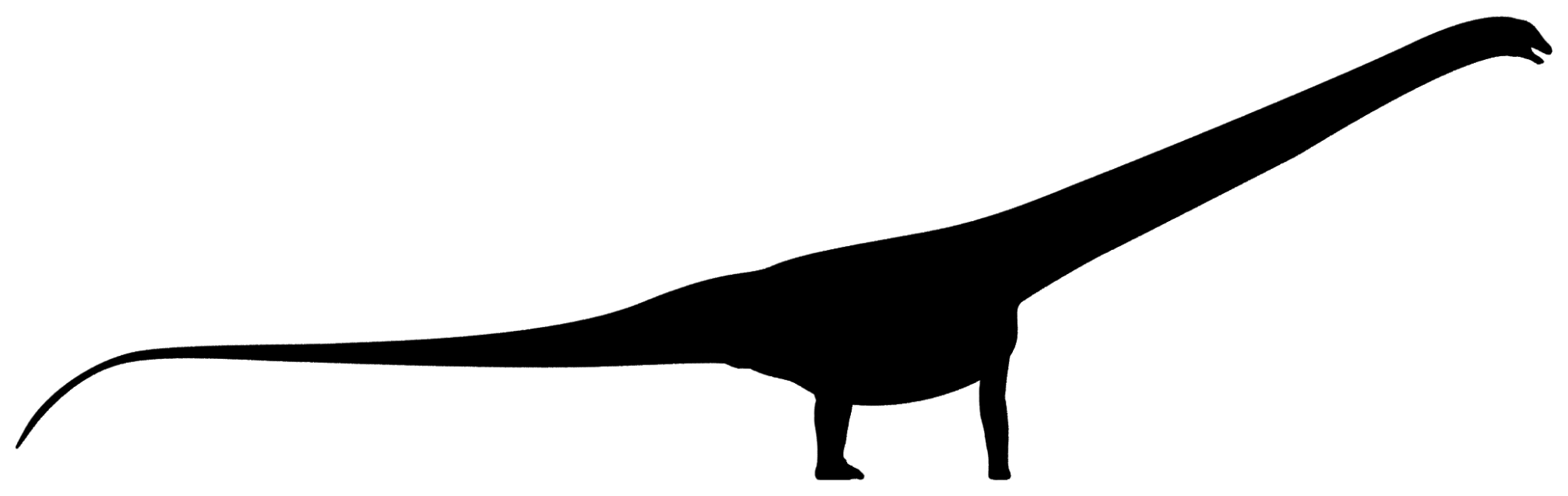  *Barosaurus* | 28095  (22797–33952) | 35059  (27849–42269) | 22379  (17692–27803) | 26322 | 34278 | 27022 |
| 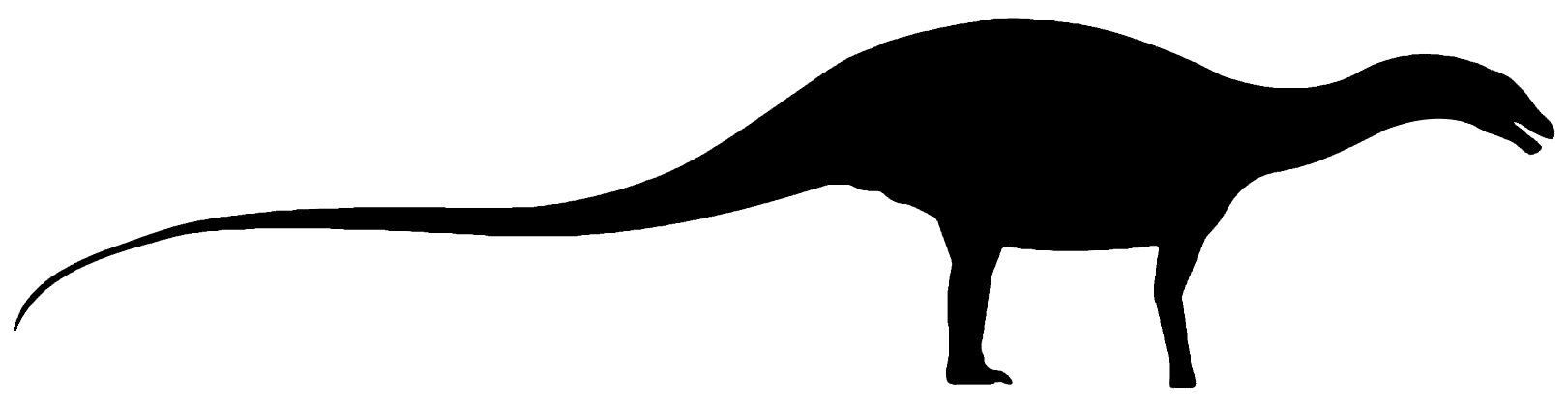  *Dicraeosaurus* | 8746  (7189– 10338) | 9291  (7609– 10973) | 7032  (5503 –8615) | 6918 | 7498 | 7201 |
| 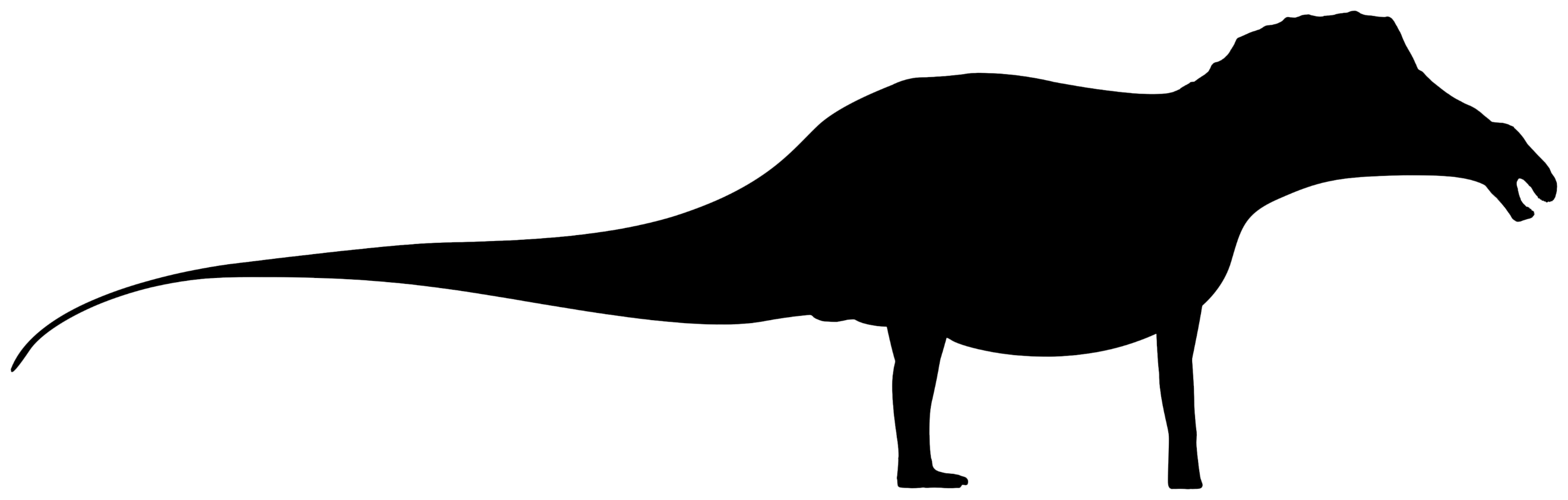  *Amargasaurus* | 5680  (4646–6714) | 6115  (5009–7222) | 4627  (3641–5631) | 4610 | 5072 | 4764 |
| 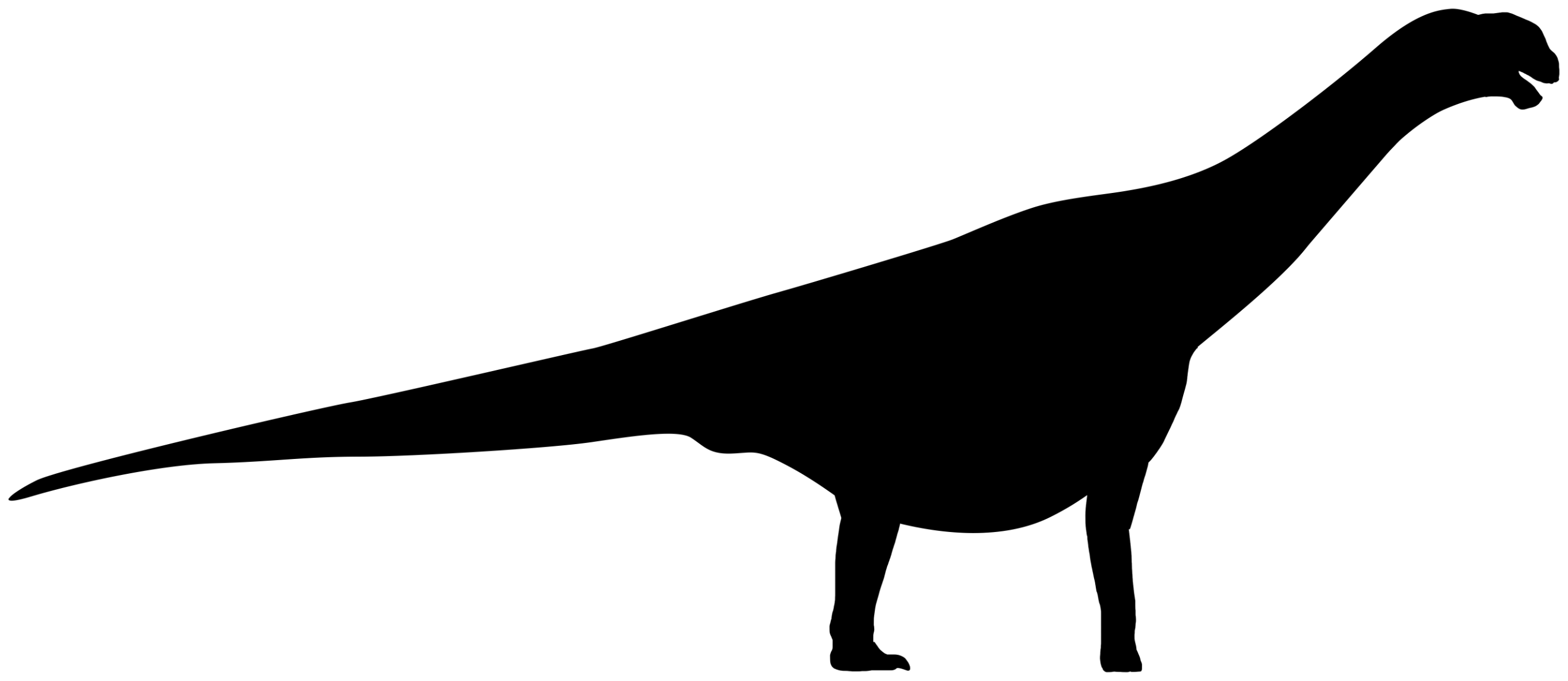  *Camarasaurus* | 24152  (20466–28093) | 26967  (22535–31399) | 19392  (15809–23285) | 18830 | 21934 | 19899 |
| 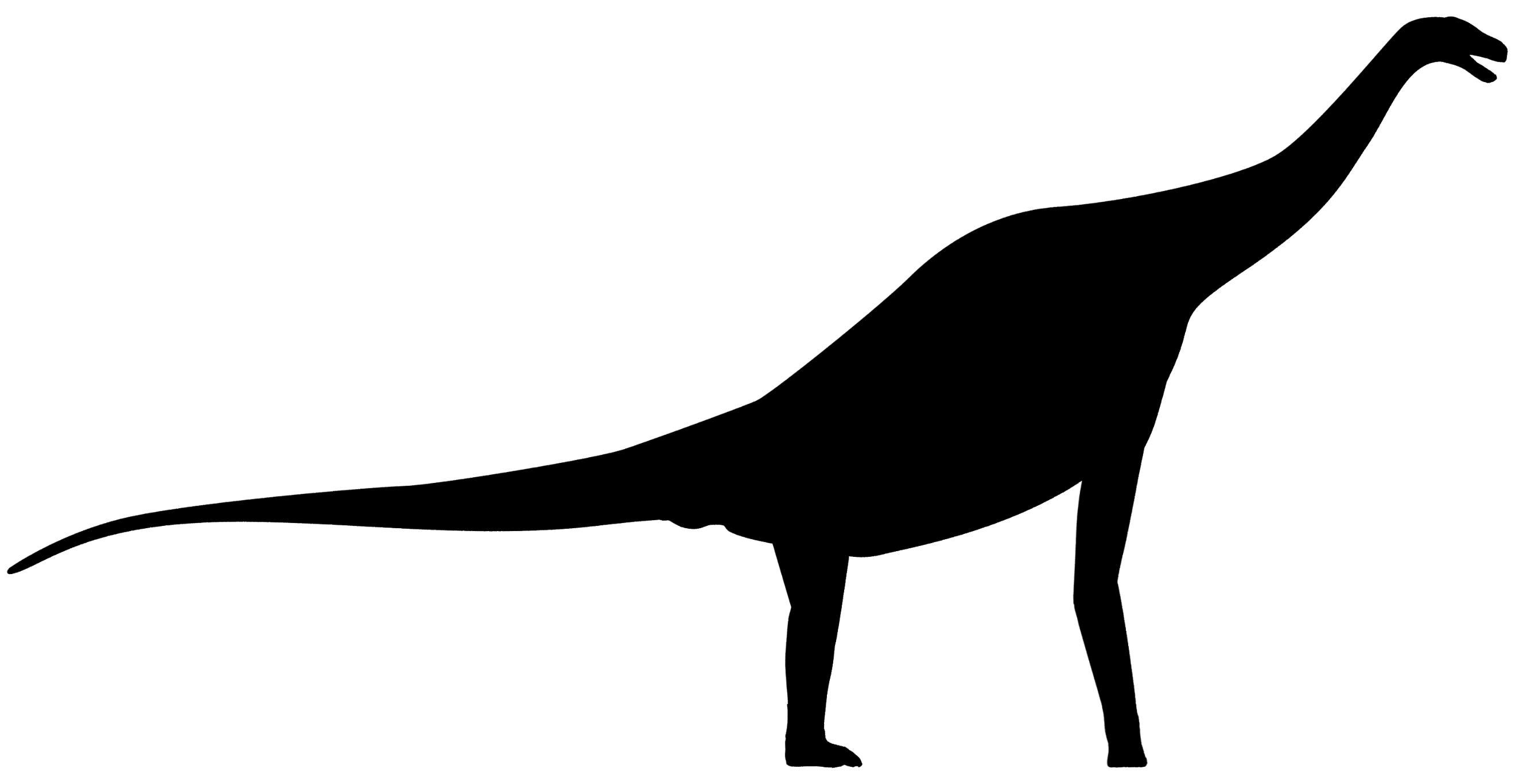  *Atlasaurus* | 30612  (25678–35628) | 31727  (26520–36933) | 23080  (18766–27652) | 21422 | 22626 | 22402 |
| 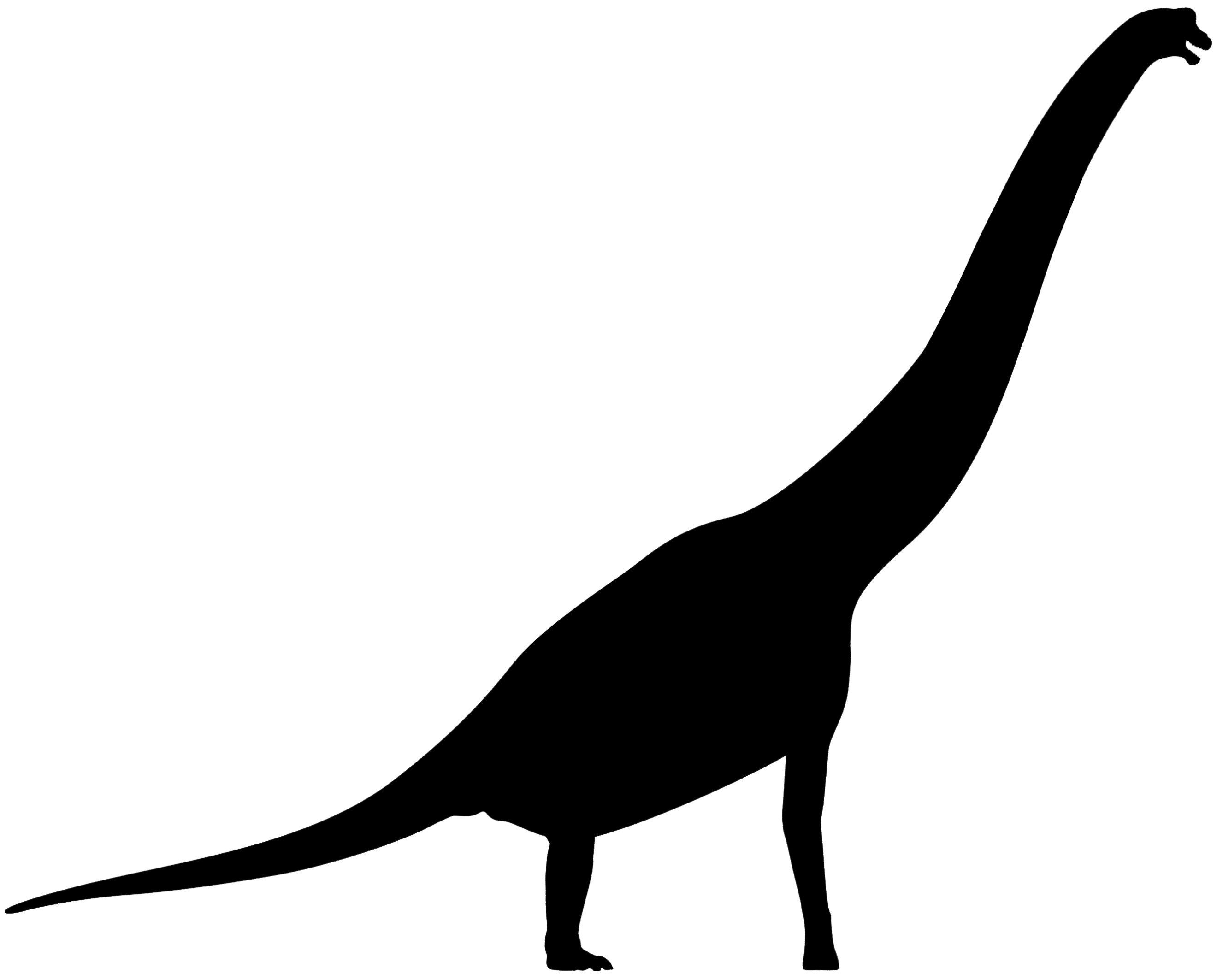  *Giraffatitan* | 47188  (40124–54736) | 52687  (44117–61258) | 35418  (29553–42066) | 34479 | 40686 | 36036 |
| 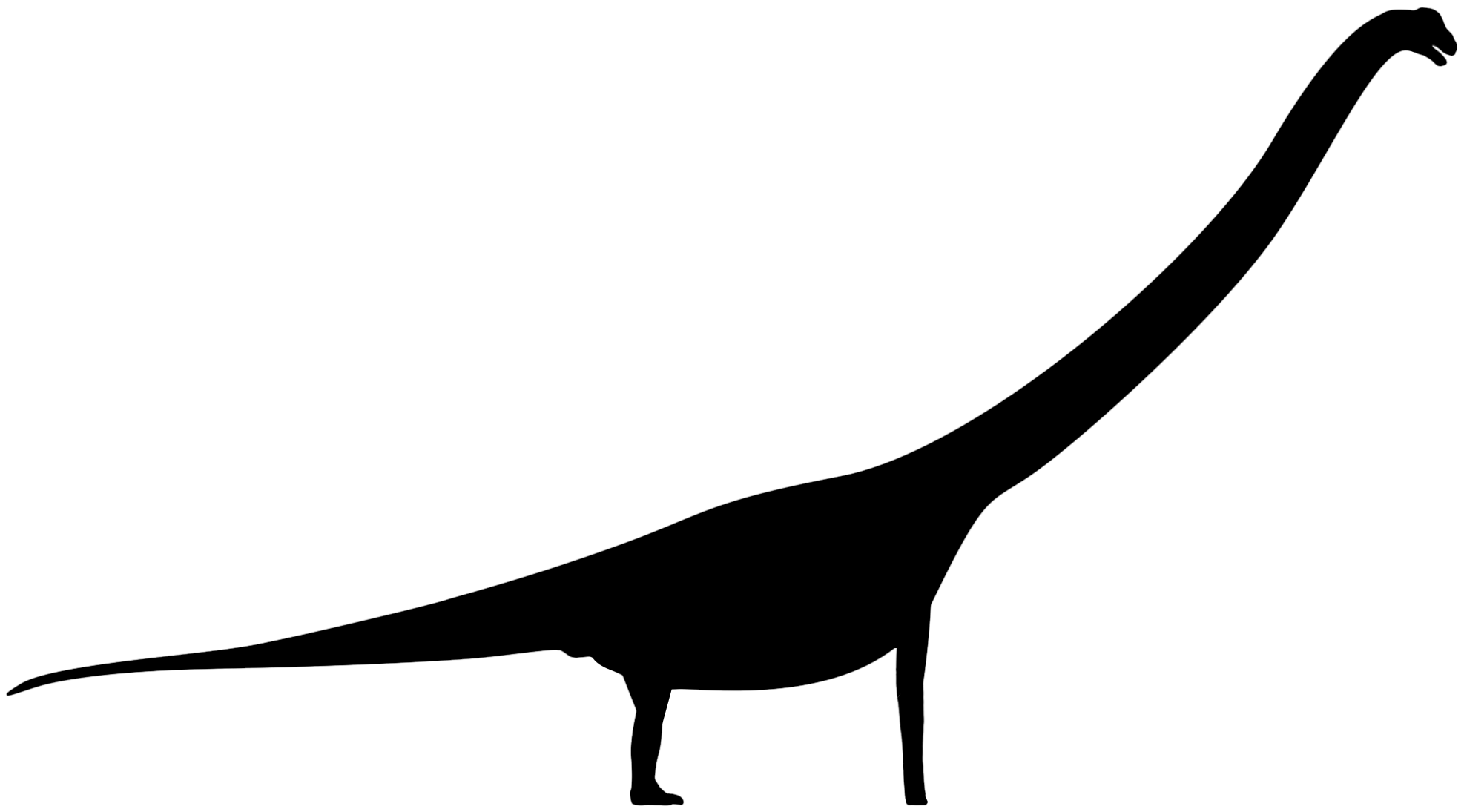  *Paluxysaurus* (=*Sauroposeidon*?) | 18316  (15563–21297) | 20870  (17445–24295) | 15455  (12738–18469) | 14860 | 17669 | 15867 |
| 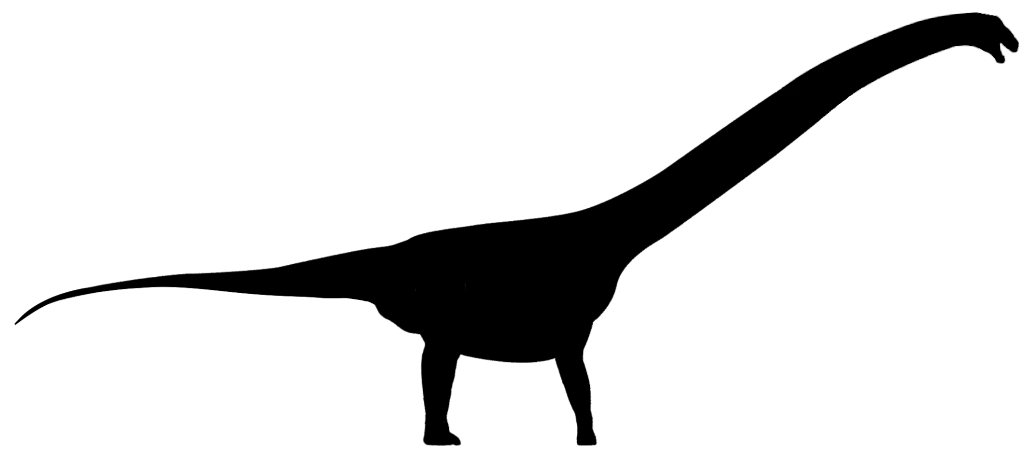  *Rapetosaurus* | 17739  (15225–20603) | 21419  (17909–24930) | 14349  (12009–17114) | 14793 | 18877 | 15587 |
| 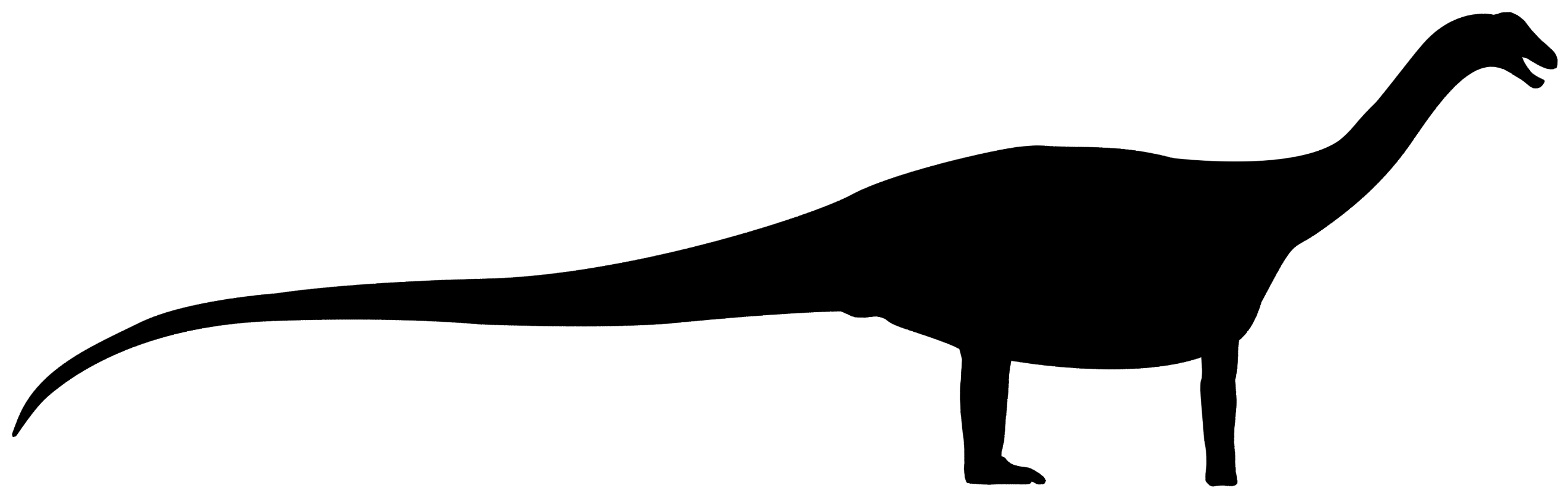  *Neuquensaurus* | 2660  (2116–3211) | 2822  (2246–3399) | 2193  (1674–2727) | 2252 | 2420 | 2307 |
| *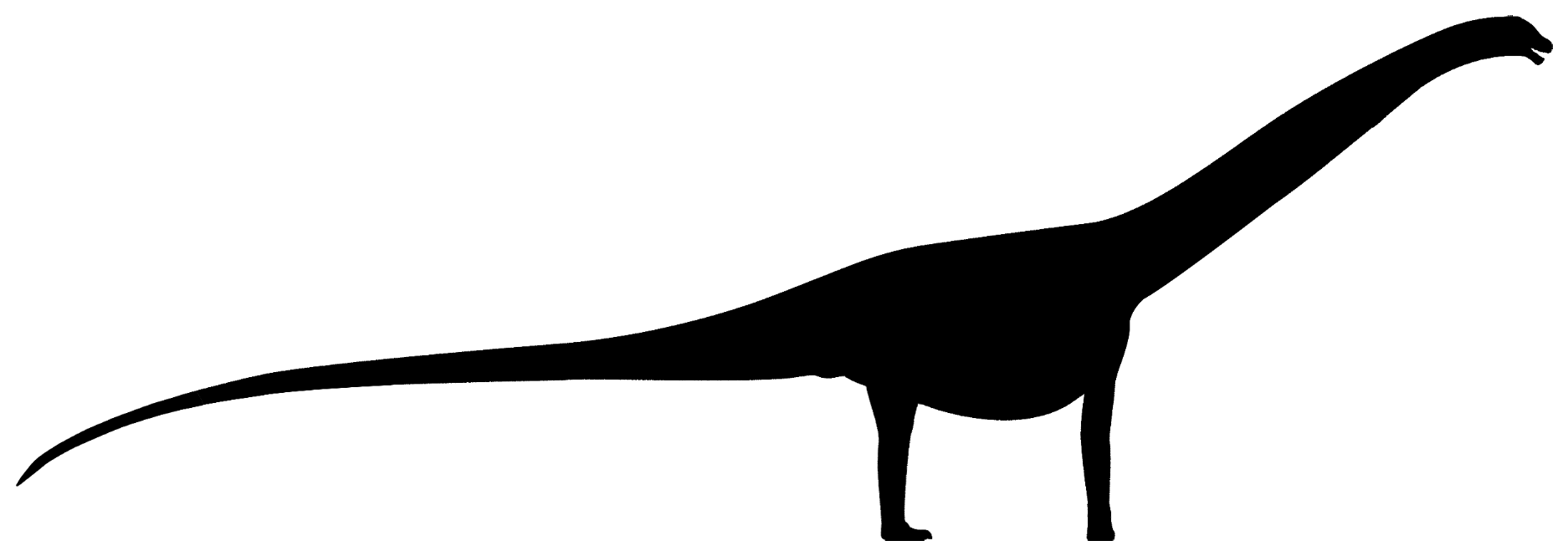*  *Patagotitan* | 81751  (68654–95663) | 95665  (78721–112608) | 66164  (53707–79703) | 67725 | 84187 | 71571 |
| 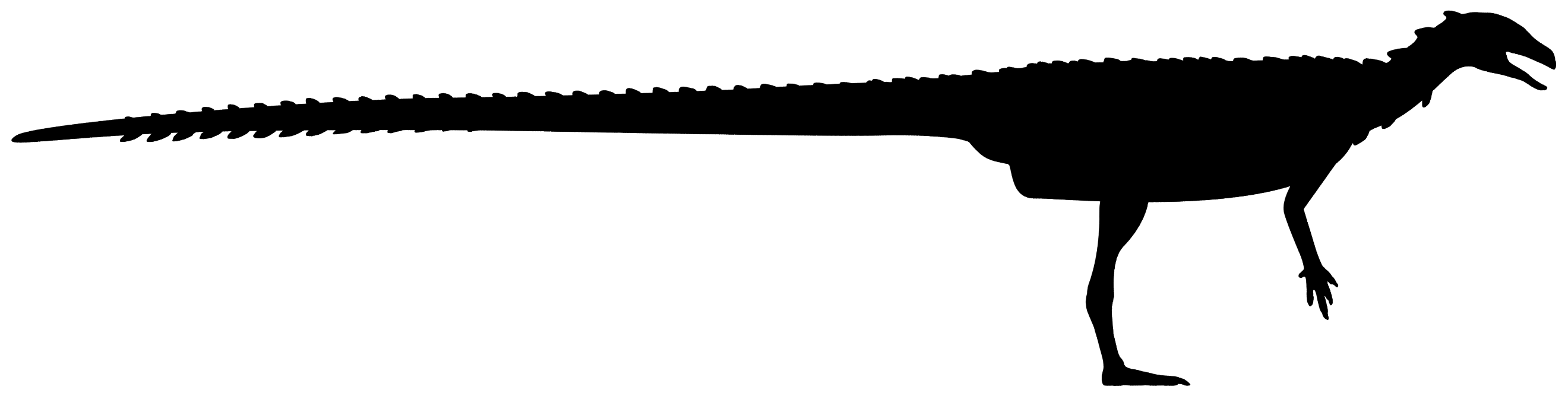  *Scutellosaurus* | 3.55  (2.87–4.23) | 3.81  (3.08–4.53) | 3.79  (2.96–4.62) | 3.66 | 3.91 | 3.94 |
| 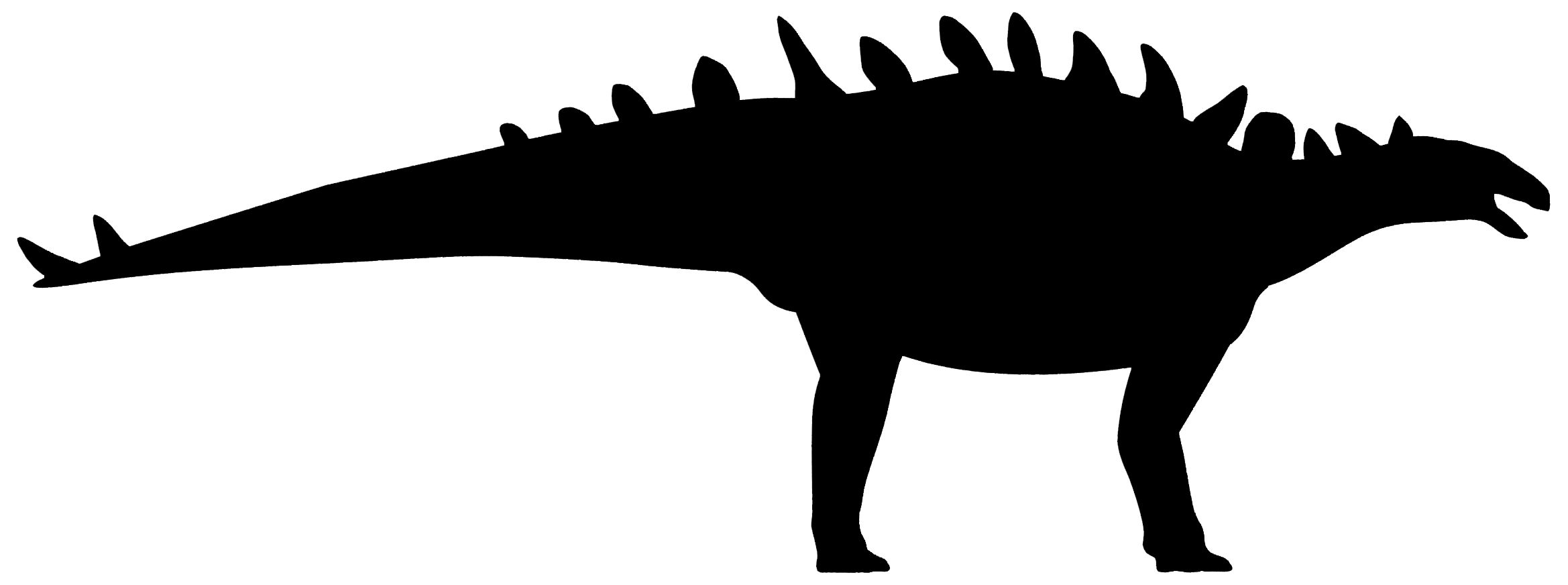  *Gigantspinosaurus* | 1769  (1496–2044) | 1826  (1543–2109) | 1596  (1302–1895) | 1456 | 1514 | 1553 |
| 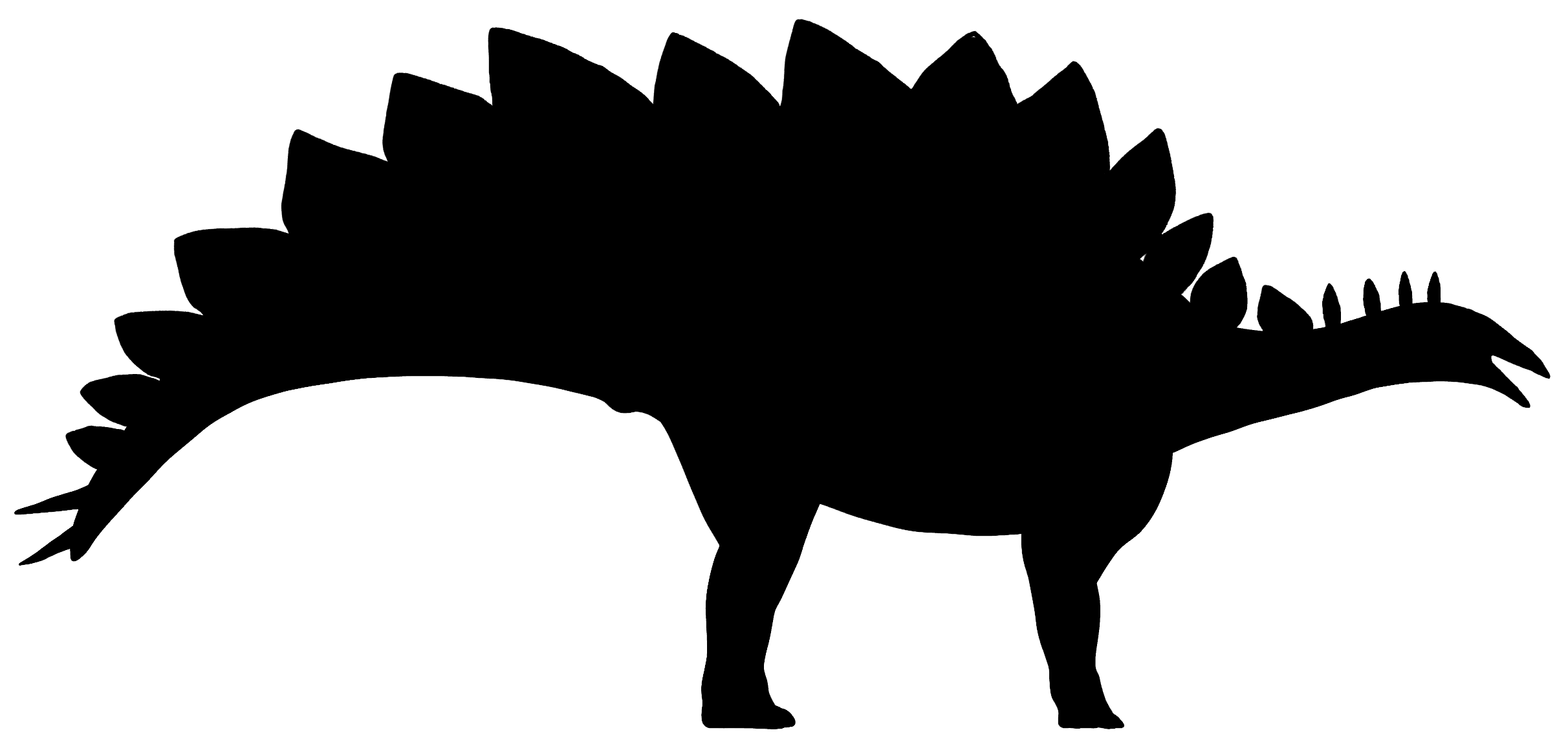  *Stegosaurus* | 2063  (1738–2388) | 2123  (1789–2458) | 1815  (1468–2167) | 1676 | 1738 | 1781 |
| 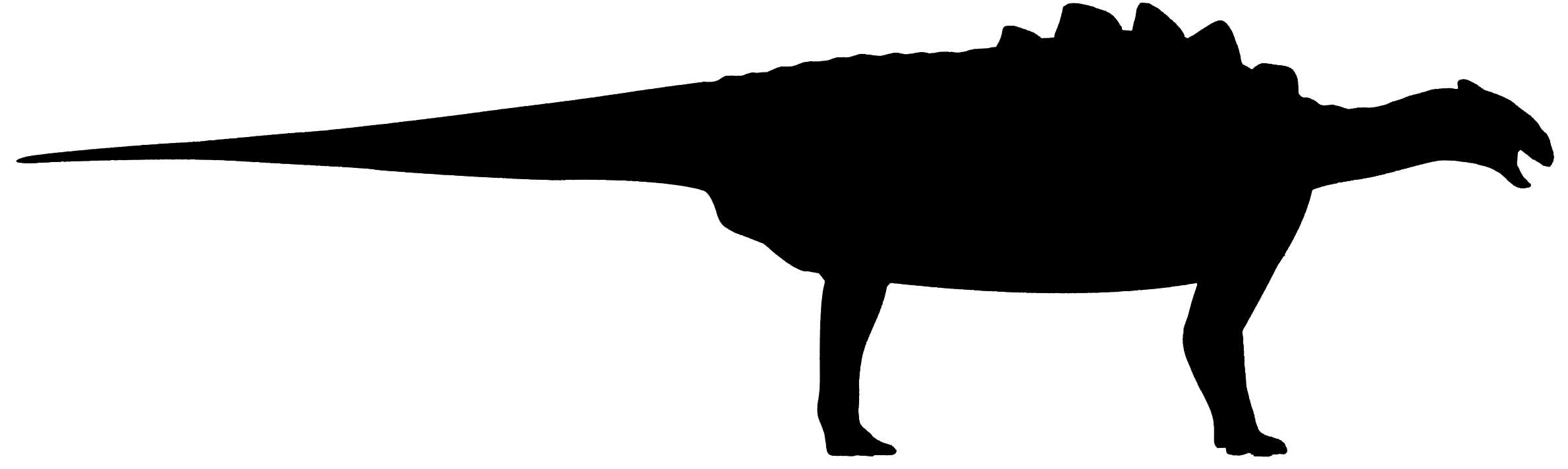  *Gastonia* | 572  (490–654) | 606  (518–695) | 557  (461–653) | 490 | 518 | 537 |
| 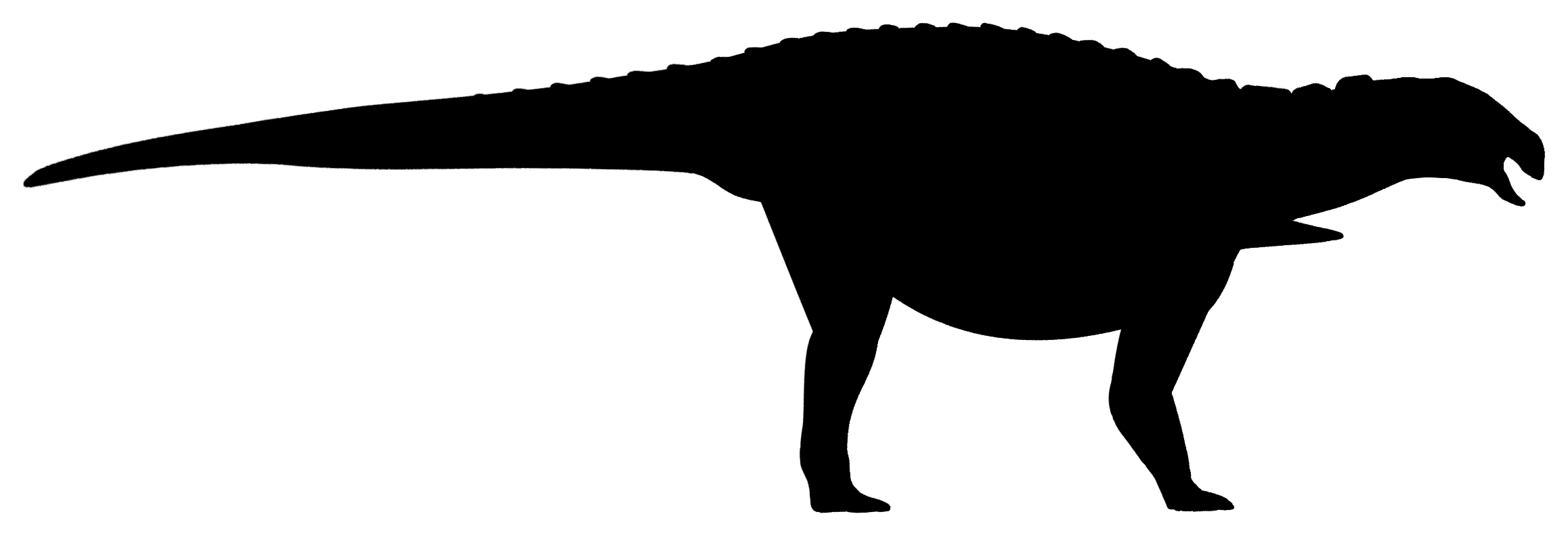  *Denversaurus* | 2787  (2389–3187) | 2976  (2544–3408) | 2557  (2117–3006) | 2232 | 2352 | 2430 |
| 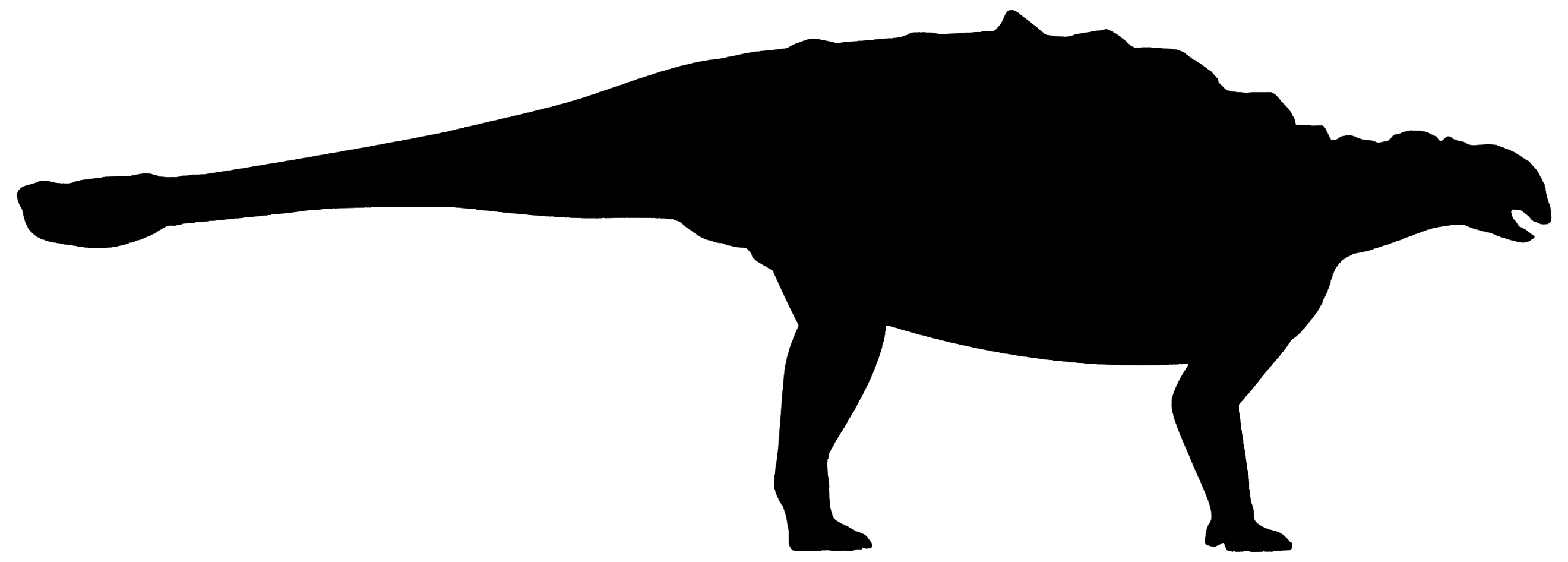  *Euoplocephalus* | 3256  (2789–3723) | 3407  (2911–3903) | 3035  (2512–3565) | 2606 | 2701 | 2856 |
| 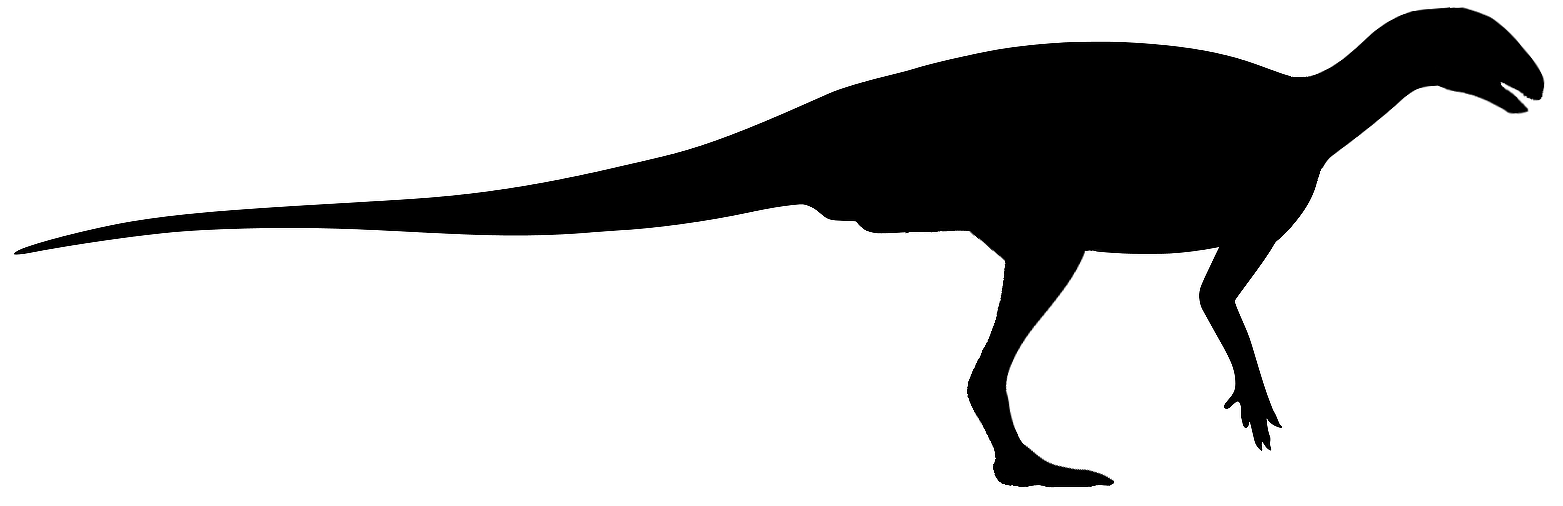  *Hypsilophodon* | 24.8  (20.0–29.7) | 27.7  (22.3–33.2) | 24.6  (18.9–30.2) | 24.1 | 26.4 | 25.4 |
| 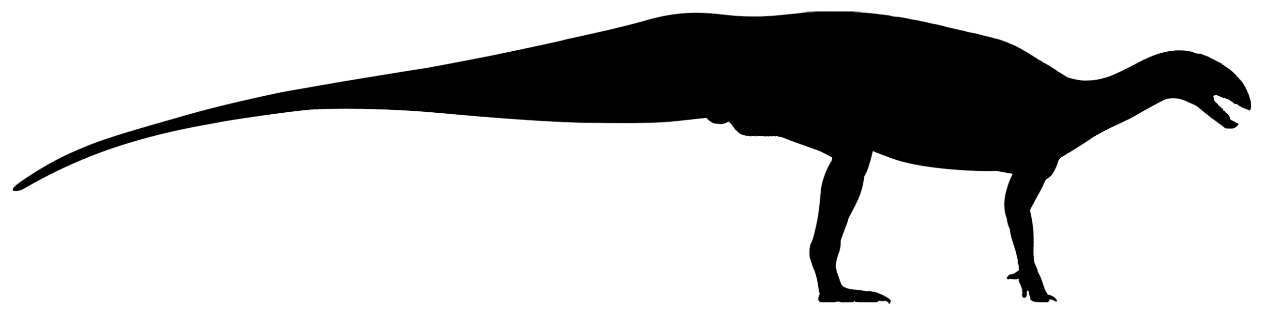  *Tenontosaurus* | 359  (297–421) | 391  (323–459) | 340  (269–411) | 314 | 336 | 338 |
| 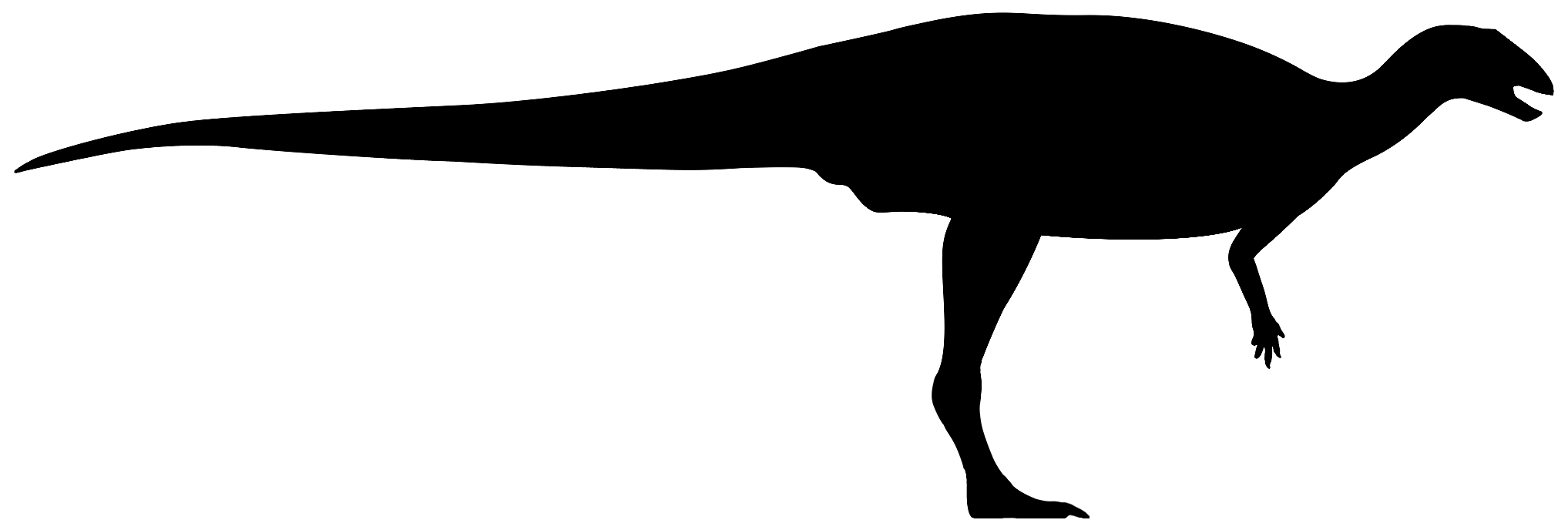  *Dysalotosaurus* | 62.6  (49.1–76.1) | 73.4  (57.6–89.3) | 60.2  (44.6–75.8) | 60.5 | 67.7 | 63.6 |
| 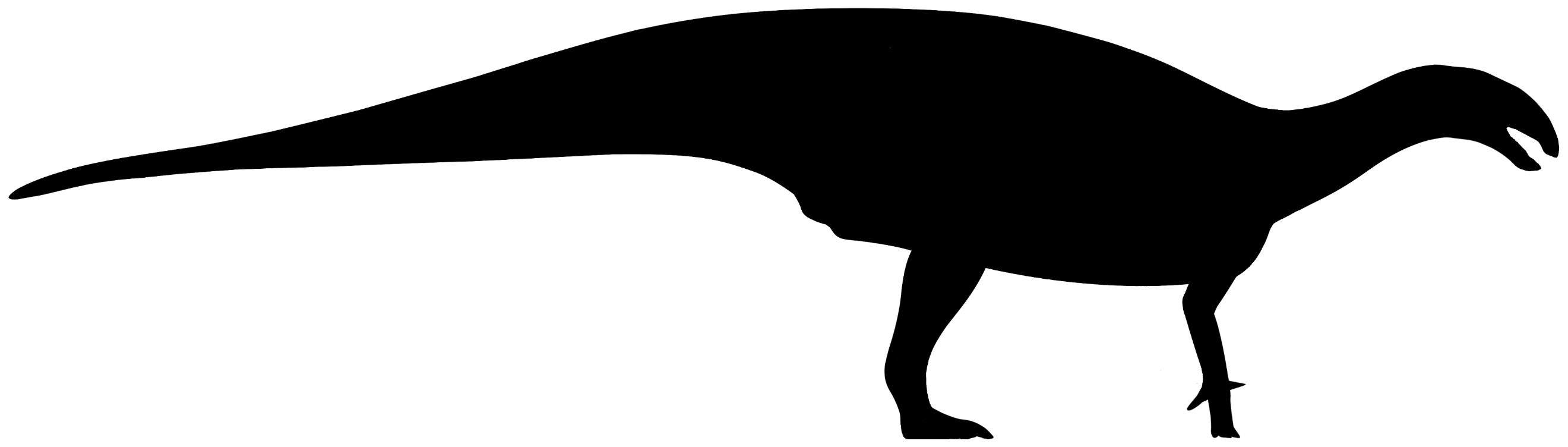  *Mantellisaurus* | 1090  (877–1304) | 1229  (989–1470) | 985  (750–1221) | 963 | 1053 | 1020 |
| 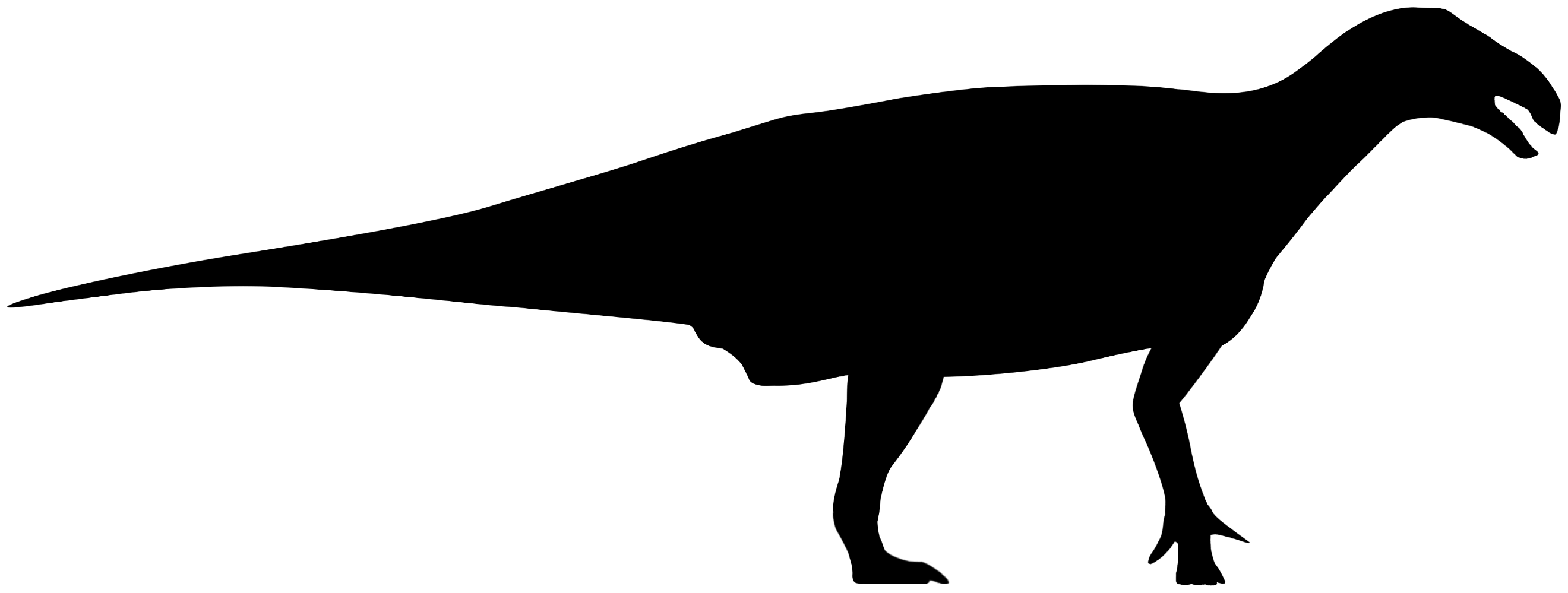  *Iguanodon* | 6213  (5162–7275) | 6887  (5690–8084) | 5375  (4289–6490) | 4960 | 5373 | 5299 |
| 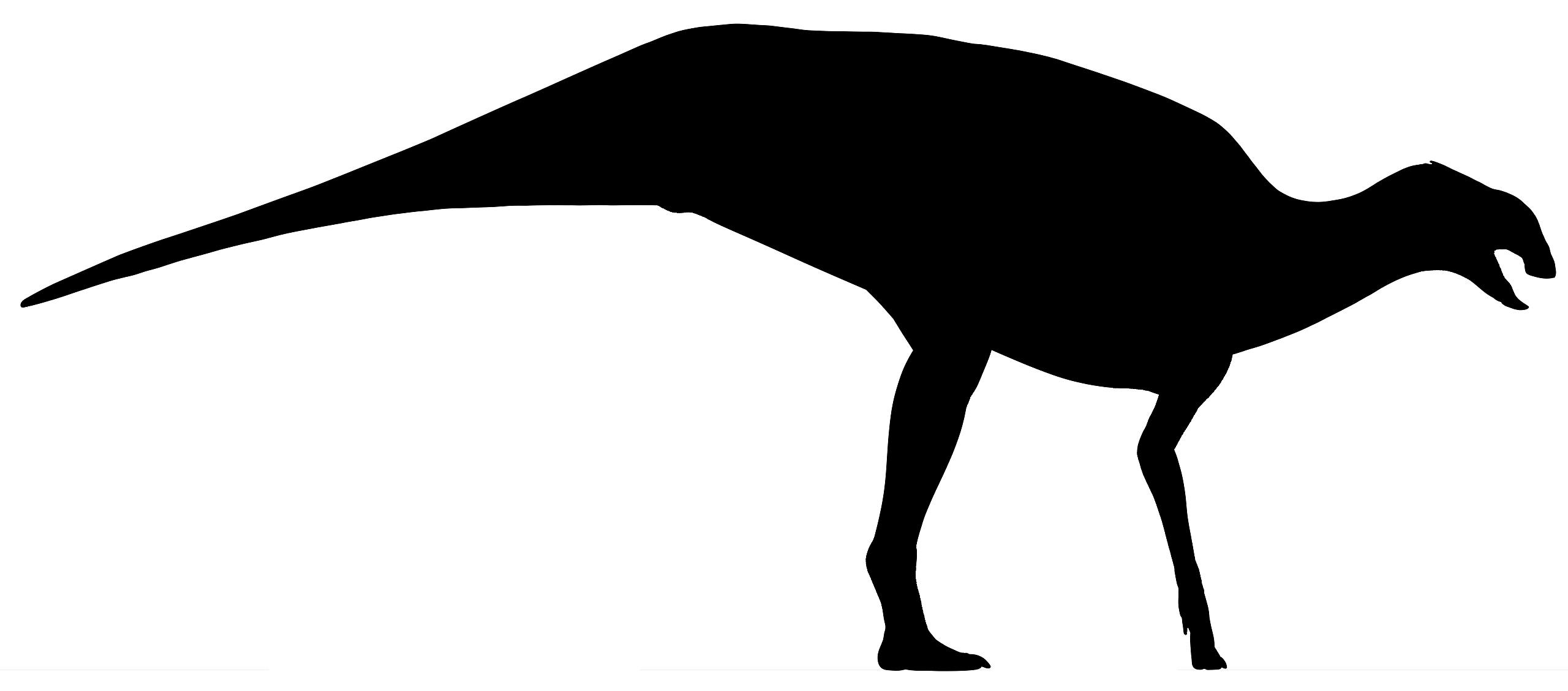  *Brachylophosaurus* | 3376  (2778–3973) | 3664  (3004–4323) | 2856  (2241–3481) | 2829 | 2943 | 2872 |
| 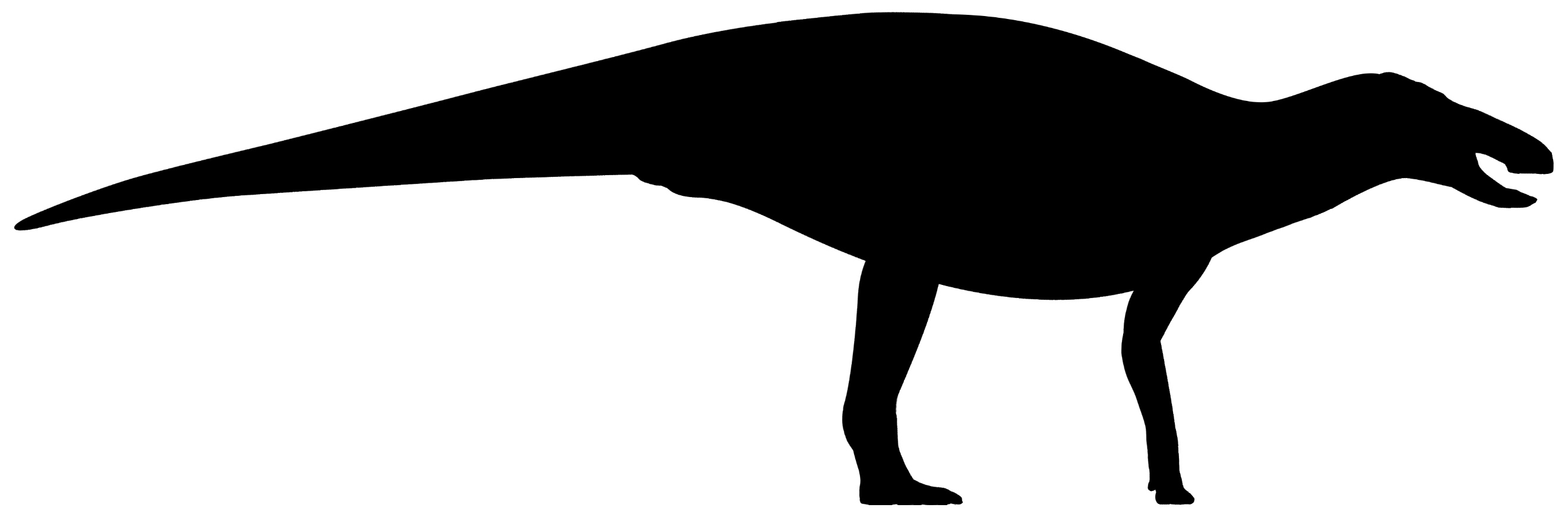  *Edmontosaurus* | 5577  (4514–6640) | 6129  (4945–7313) | 4571  (3508–5649) | 4658 | 4867 | 4699 |
| 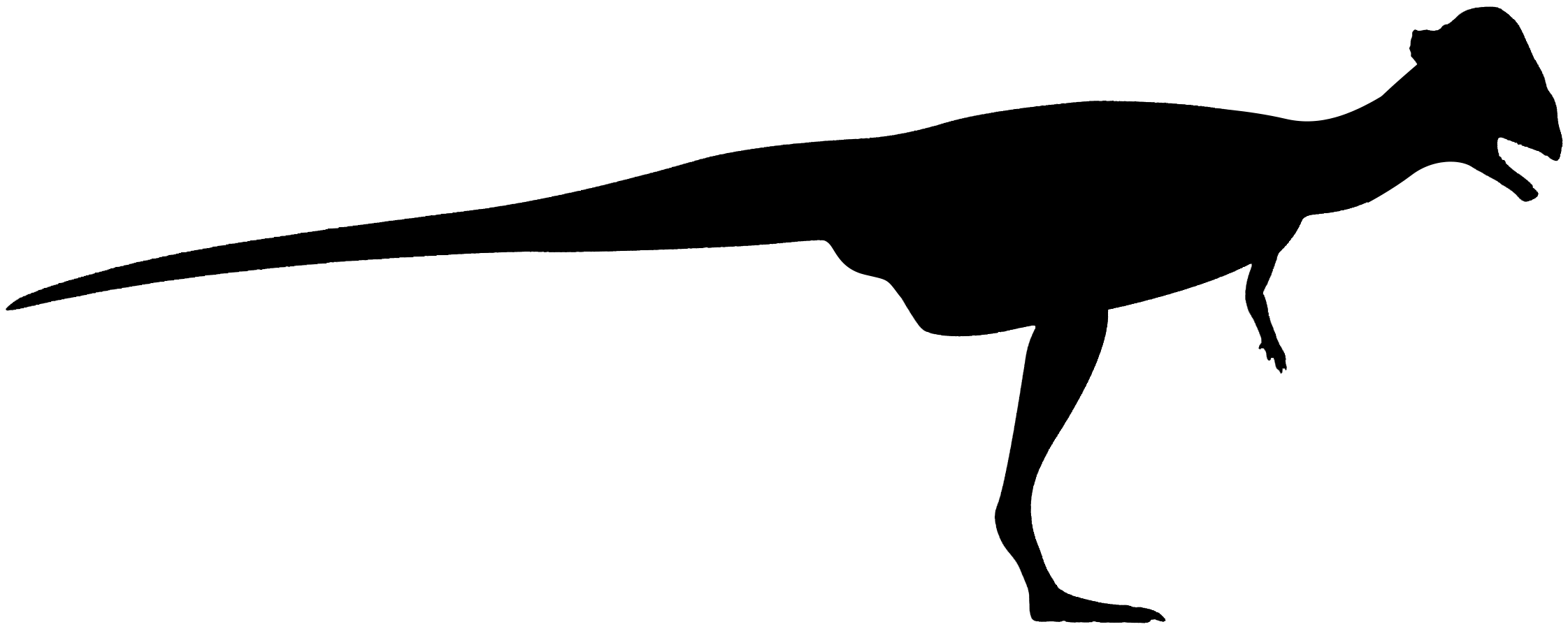  *Stegoceras* | 19.0  (15.3–22.7) | 21.0  (17.0–25.0) | 19.4  (15.1–23.7) | 18.9 | 20.4 | 20.0 |
| 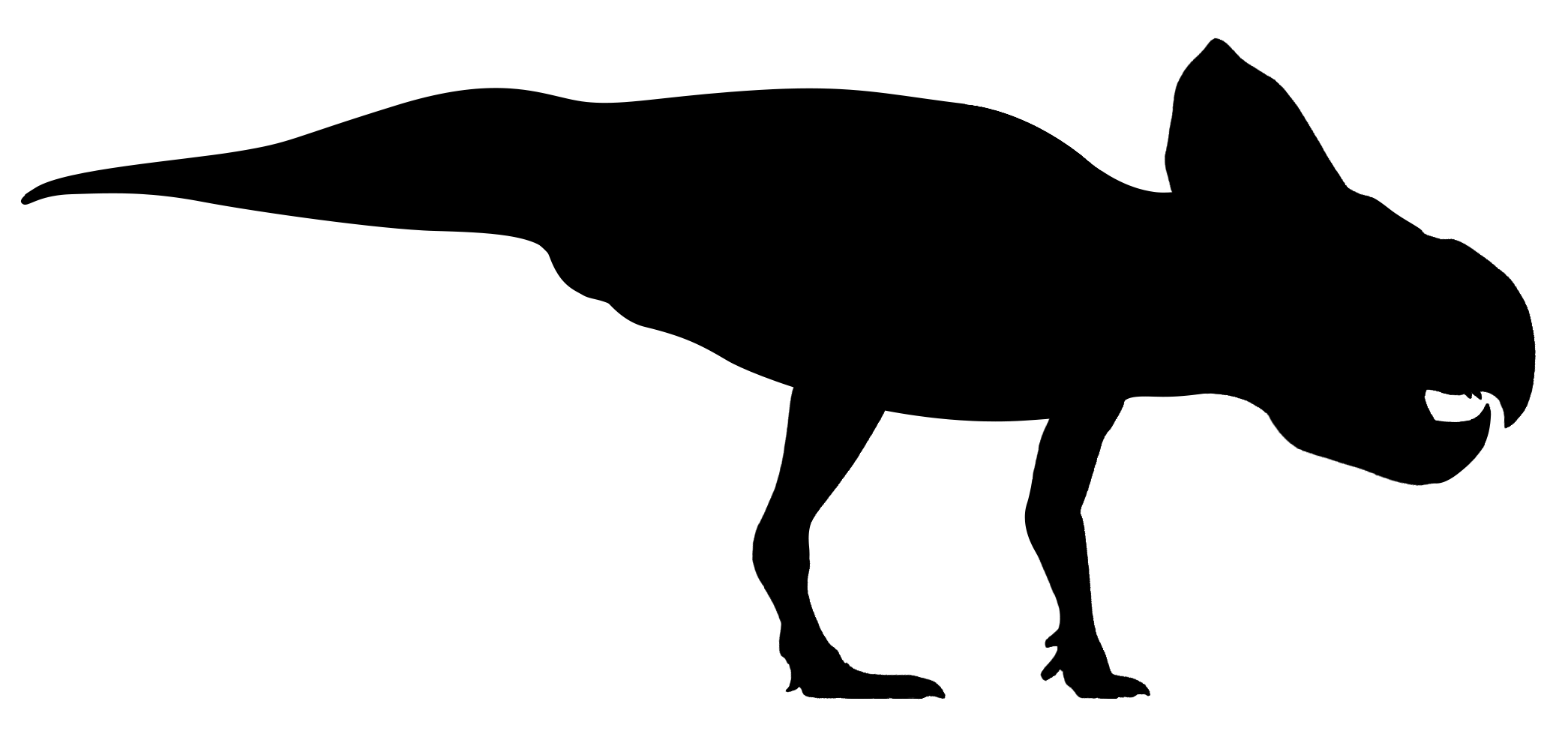  *Protoceratops* | 40.5  (33.0–48.0) | 43.9  (35.6–52.3) | 36.4  (29.1–43.7) | 40.1 | 42.2 | 38.2 |
| 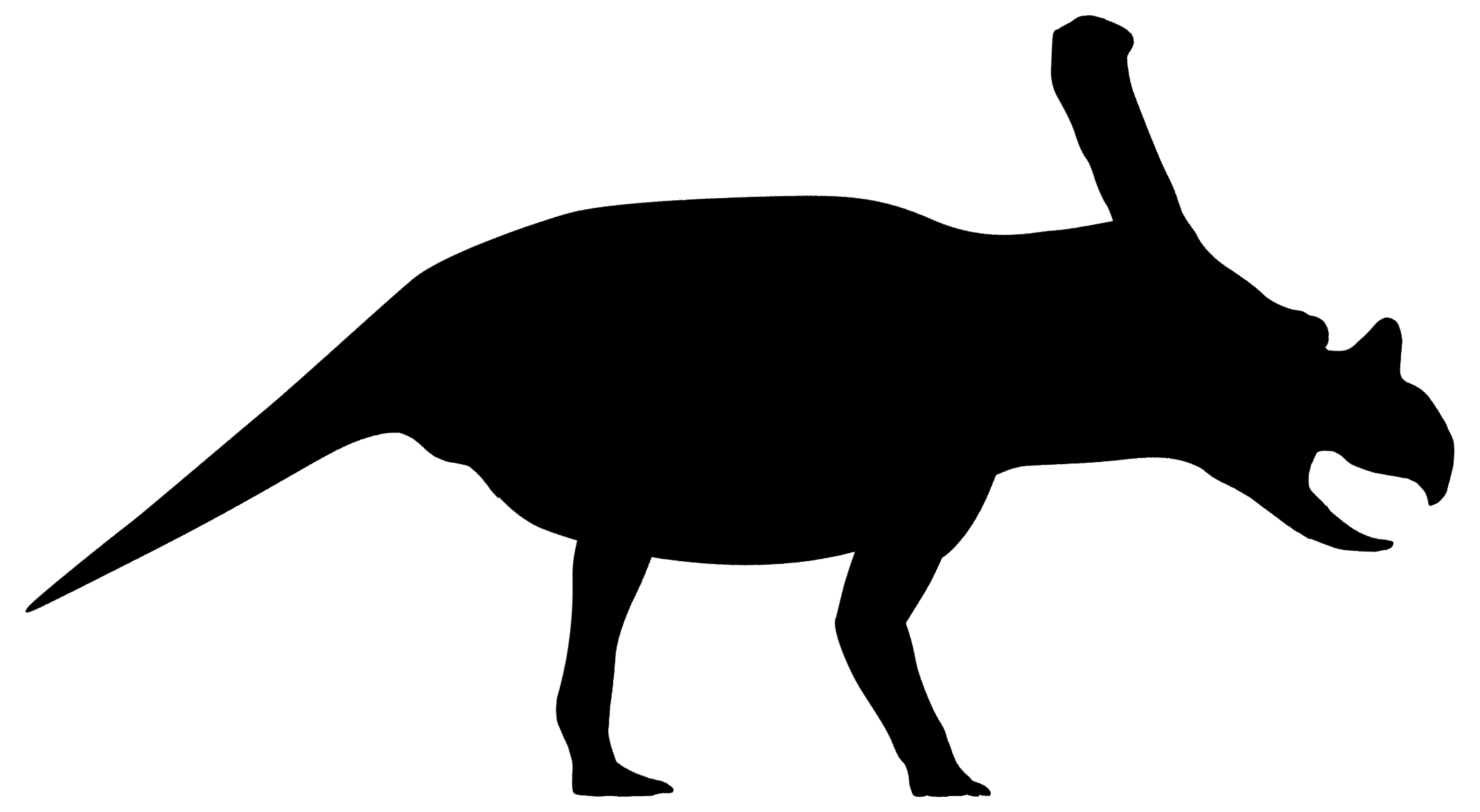  *Chasmosaurus* | 1907  (1631–2184) | 1978  (1688–2269) | 1569  (1311–1836) | 1575 | 1606 | 1559 |
| 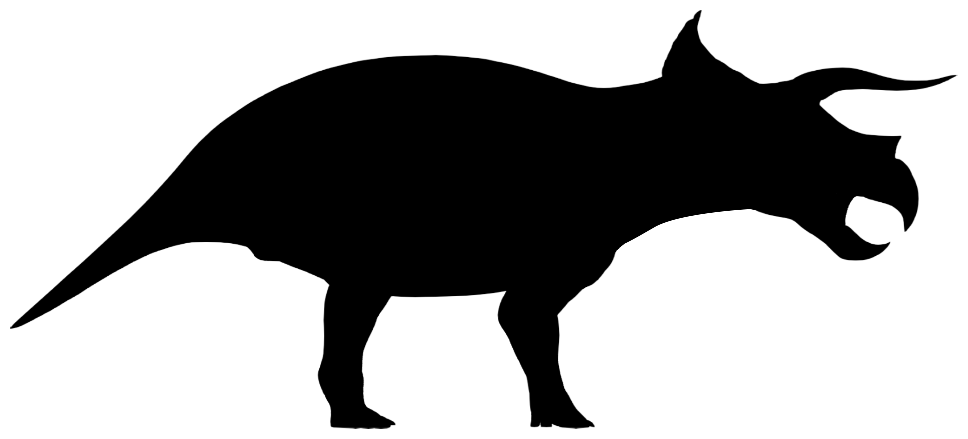  *Triceratops* | 8344  (7085–9602) | 8689  (7366–10011) | 6387  (5310–7520) | 6586 | 6712 | 6383 |

**Table S5.** (see separate file, Table_S5.xlsx). Data from variant models used in the sensitivity analyses.

**Table S6.** (see separate file, Table_S6.xlsx). Complete results for all body segment and centre of mass regressions.


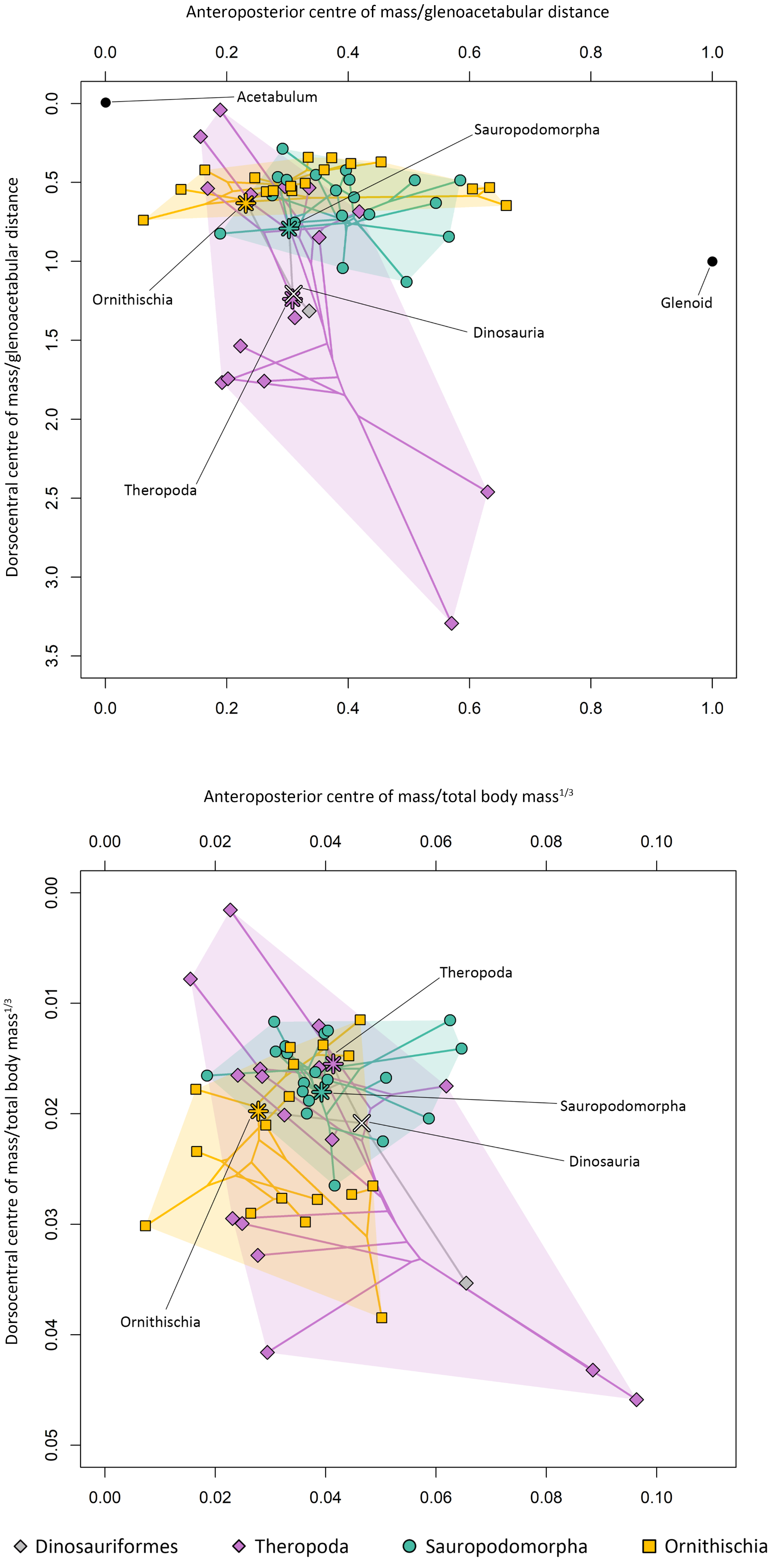


**Fig. S6.** Phylomorphospace scatter plots illustrating whole-body centre of mass evolution across Dinosauria, based on the preferred allometric model set.


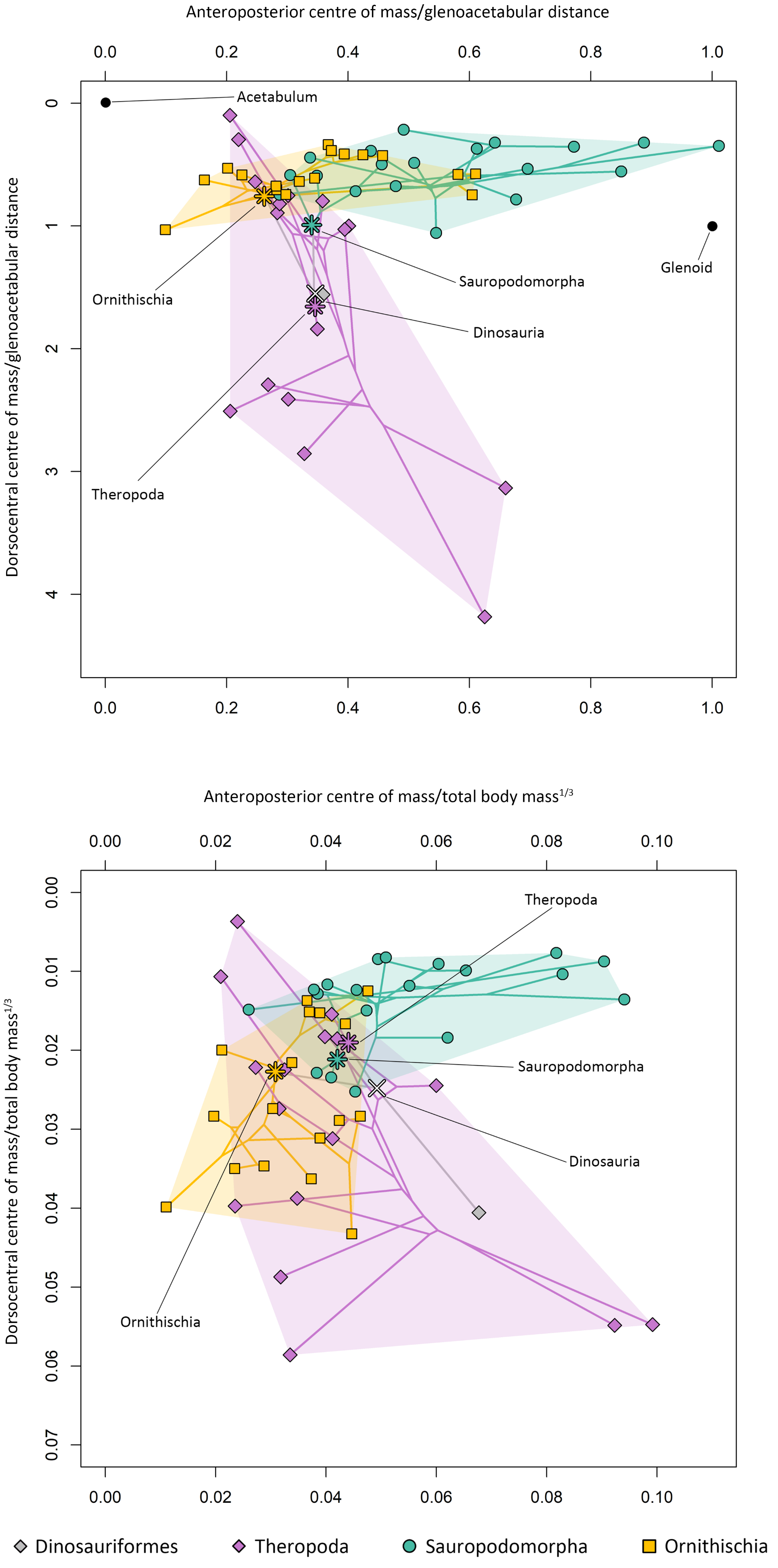


**Fig. S7.** Phylomorphospace scatter plots illustrating whole-body centre of mass evolution across Dinosauria, based on the non-avian sauropsid allometric model set.


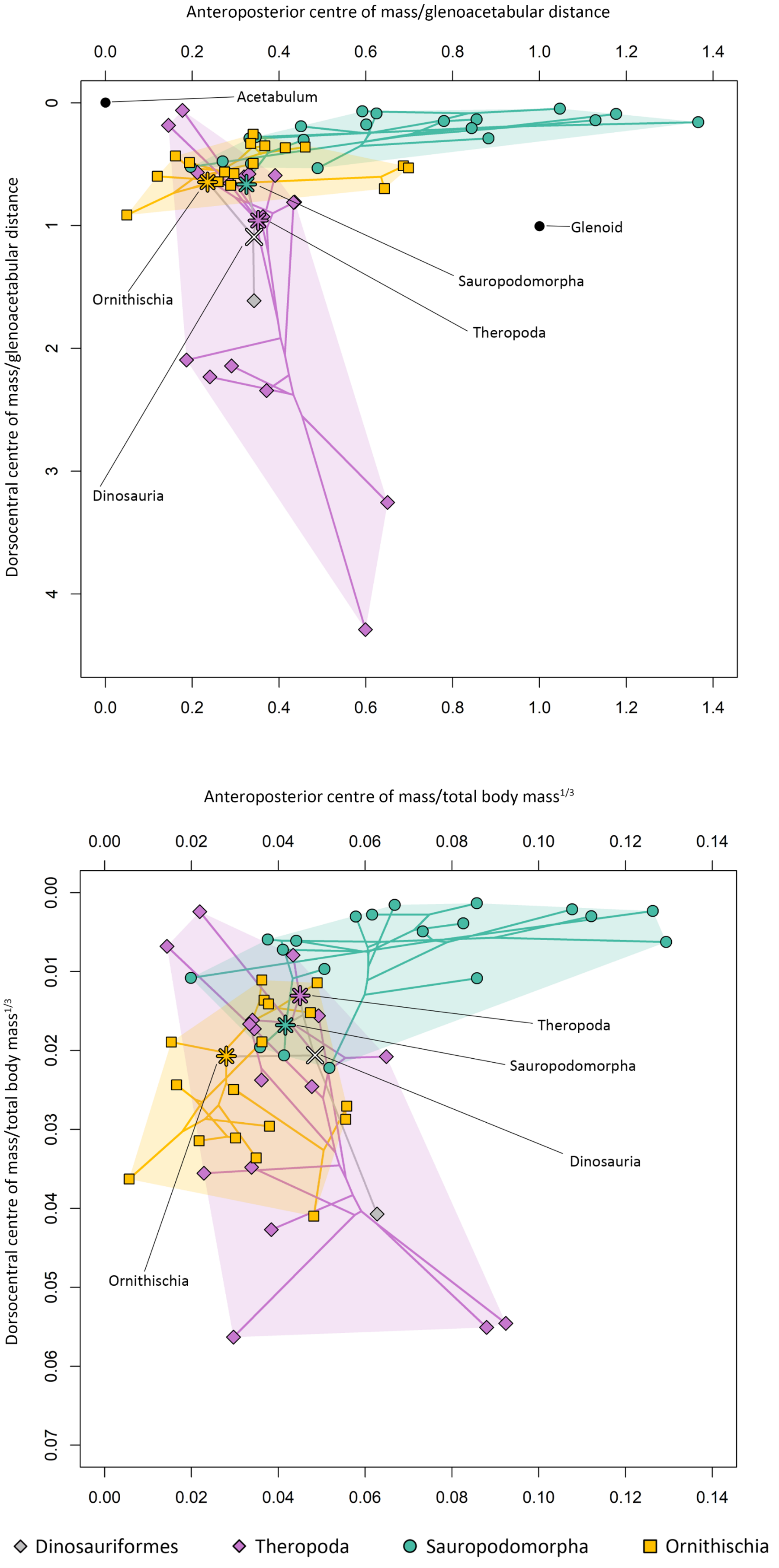


**Fig. S8.** Phylomorphospace scatter plots illustrating whole-body centre of mass evolution across Dinosauria, based on the non-avian sauropsid isometric model set.

**
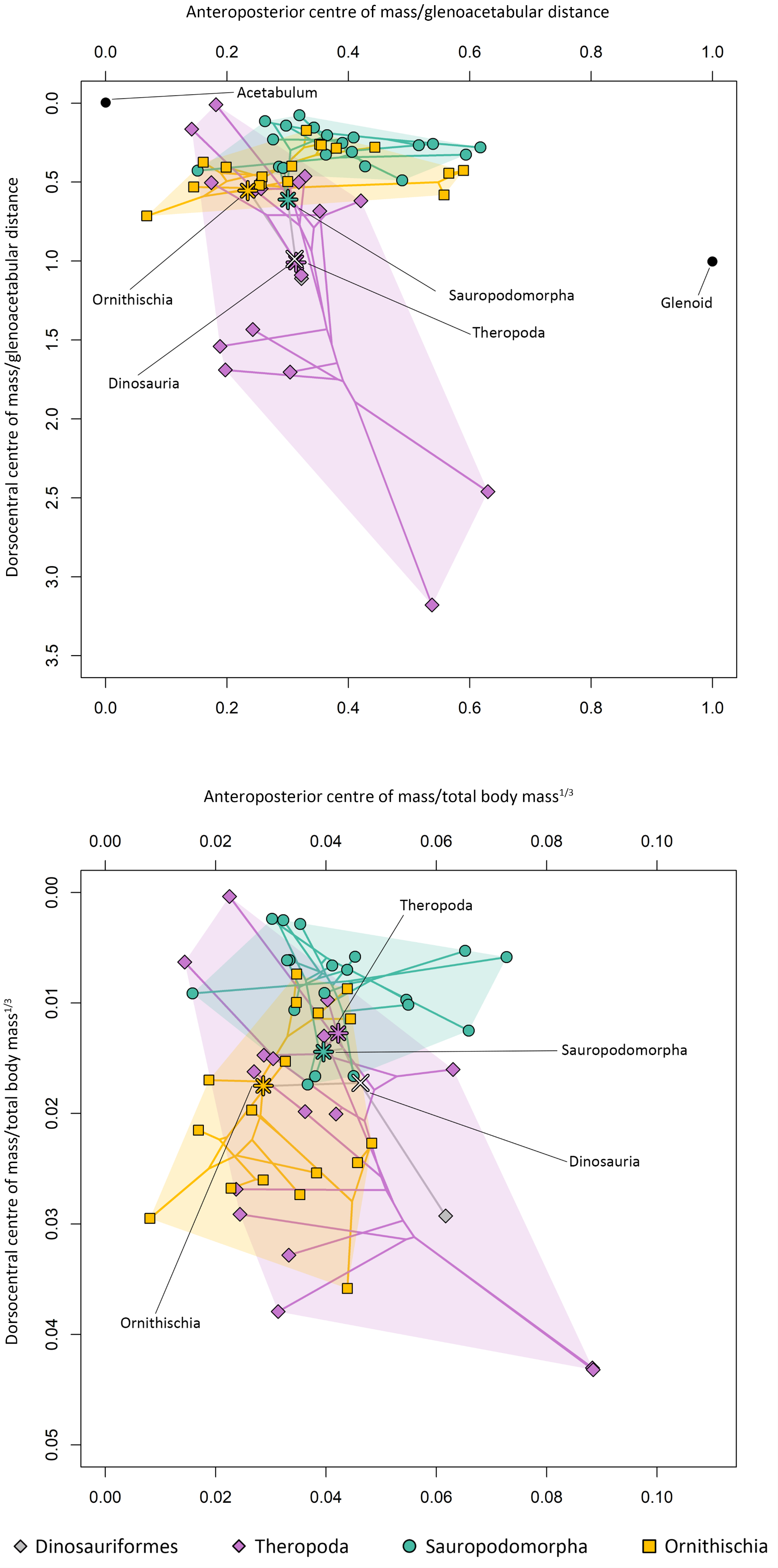
**

**Fig. S9.** Phylomorphospace scatter plots illustrating whole-body centre of mass evolution across Dinosauria, based on the bird allometric model set.

**
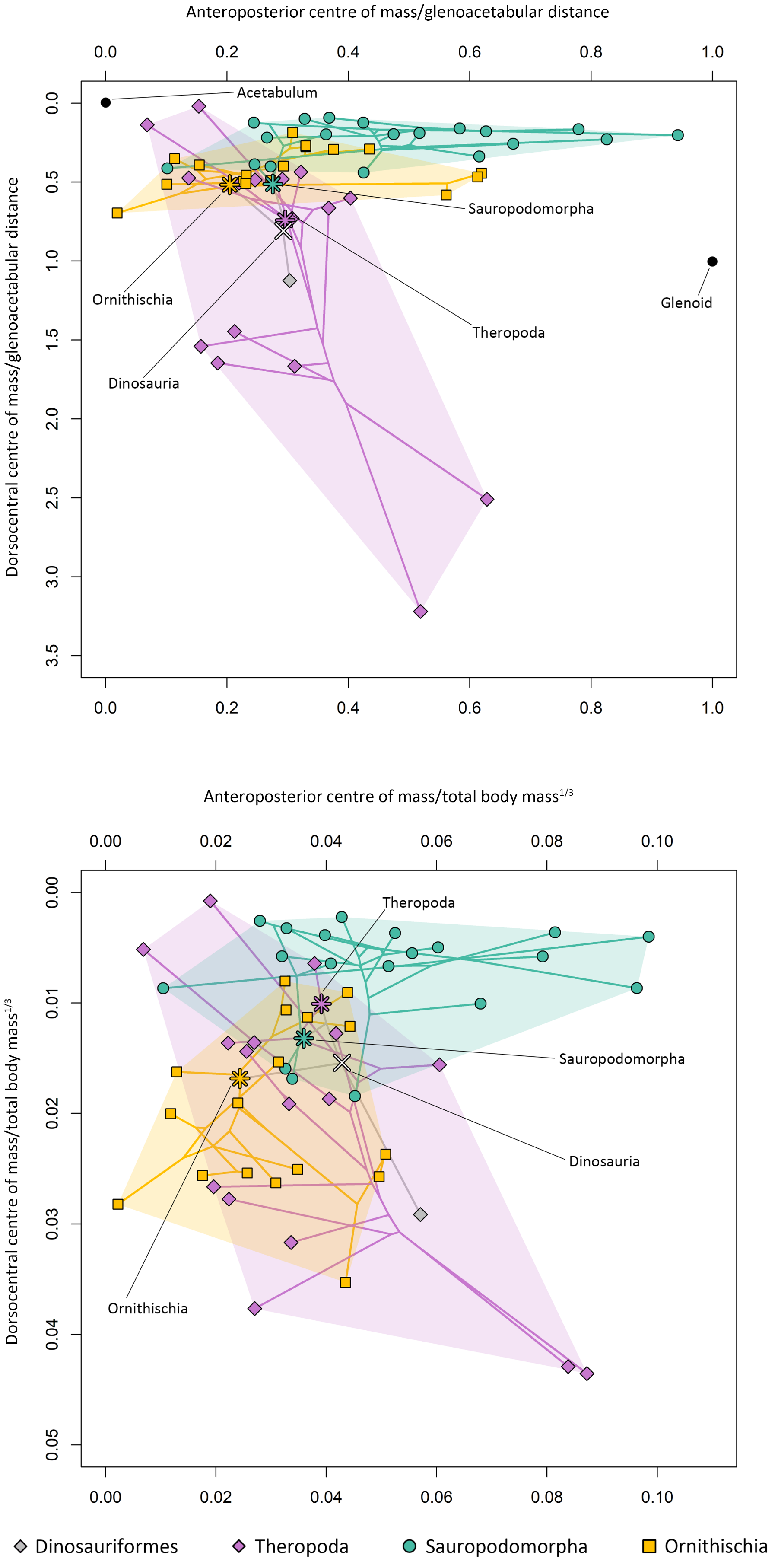
**

**Fig. S10.** Phylomorphospace scatter plots illustrating whole-body centre of mass evolution across Dinosauria, based on the bird isometric model set.

**REFERENCES**

Allen, V., Bates, K. T., Li, Z. & Hutchinson, J. R. (2013). Linking the evolution of body shape and biomechanics in bird–line archosaurs. *Nature* **497**, 104–107.

Allen, V., Molnar, J., Parker, W., Pollard, A., Nolan, G. and Hutchinson, J. R. (2014). Comparative architectural properties of limb muscles in Crocodylidae and Alligatoridae and their relevance to divergent use of asymmetrical gaits in extant Crocodylia. *Journal of Anatomy* **225**, 569–582.

Anderson, L., Brasset, C., Pond, S., Bates, K. & Sellers, W. I. (2023). Investigating the quadrupedal abilities of *Scutellosaurus lawleri* and its implications for locomotor behavior evolution among dinosaurs. *The Anatomical Record* **306**, 2514–2526.

Apaldetti, C., Pol, D., & Yates, A. (2012). The postcranial anatomy of *Coloradisaurus brevis* (Dinosauria: Sauropodomorpha) from the Late Triassic of Argentina and its phylogenetic implications.*Palaeontology* **56**, 277-301.

Armas, P., Moreno, C., Sánchez, M. L., & González, F. (2014). Sedimentary palaeoenvironment, petrography, provenance and diagenetic inference of the Anacleto Formation in the Neuquén Basin, Late Cretaceous, Argentina. *Journal of South American Earth Sciences* **53**, 59-76.

Augustin, F. J., Matzke, A. T., Maisch, M. W., & Pfretzschner, H. U. (2020). First evidence of an ankylosaur (Dinosauria, Ornithischia) from the Jurassic Qigu Formation (Junggar Basin, NW China) and the early fossil record of Ankylosauria. Geobios **61**, 1-10.

Averianov, A. O., Krasnolutskii, S. A., & Ivantsov, S. V. (2010). A new basal coelurosaur (Dinosauria: Theropoda) from the Middle Jurassic of Siberia. *Proceedings of the Zoological Institute RAS* **314**, 42-57.

Bajpai, S., Datta, D., Pandey, P., Ghosh, T., Kumar, K., & Bhattacharya, D. (2023). Fossils of the oldest diplodocoid dinosaur suggest India was a major centre for neosauropod radiation. *Scientific Reports* **13**, 12680.

Bandyopadhyay, S., Gillette, D. D., Ray, S., & Sengupta, D. P. (2010). Osteology of *Barapasaurus tagorei* (Dinosauria: Sauropoda) from the early Jurassic of India. *Palaeontology* **53**, 533-569.

Barrett, P. M., Butler, R. J., Mundil, R., Scheyer, T. M., Irmis, R. B., & Sánchez-Villagra, M. R. (2014). A palaeoequatorial ornithischian and new constraints on early dinosaur diversification. *Proceedings of the Royal Society B: Biological Sciences* **281**, 20141147.

Bates, K. T., Benson, R. B. J. & Falkingham P. L. (2012). A computational analysis of locomotor anatomy and body mass evolution in Allosauroidea (Dinosauria: Theropoda). *Paleobiology* **38**, 486–507.

Bates, K. T., Manning, P.L. Hodgetts, D. & Sellers, W. I. (2009*a*). Estimating Mass Properties of Dinosaurs Using Laser Imaging and 3D Computer Modelling. *PLoS ONE* **4**, e4532.

Bates, K. T., Mannion, P. D., Falkingham, P. L., Brusatte, S. L., Hutchinson, J. R., Otero, A, Sellers, W. I., Sullivan, C., Stevens, K. A., & Allen, V. (2016). Temporal and phylogenetic evolution of the sauropod dinosaur body plan. *Royal Society Open Science* **3**, 150636.

Bertozzo, F., Manucci, F., Dempsey, M., Tanke, D. H., Evans, D. C., Ruffell, A. & Murphy, E. (2020). Description and etiology of paleopathological lesions in the type specimen of *Parasaurolophus walkeri* (Dinosauria: Hadrosauridae), with proposed reconstructions of the nuchal ligament. *Journal of Anatomy* **238**, 1055–1069.

Beveridge, T. L., Roberts, E. M., Ramezani, J., Titus, A. L., Eaton, J. G., Irmis, R. B., & Sertich, J. J. (2022). Refined geochronology and revised stratigraphic nomenclature of the Upper Cretaceous Wahweap Formation, Utah, USA and the age of early Campanian vertebrates from southern Laramidia. *Palaeogeography, Palaeoclimatology, Palaeoecology* **591**, 110876.

Biewener, A. A. (2011). Muscle function in avian flight: achieving power and control. Philosophical Transactions of The Royal Society B: Biological Sciences **336**, 1496–1506.

Bishop, P. J., Falisse, A., De Groote, F. & Hutchinson, J. R. (2021*b*). Predictive simulations of running gait reveal a critical dynamic role for the tail in bipedal dinosaur locomotion. *Science Advances* **7**, eabi7348.

Bonsor, J. A., Lockwood, J. A. F., Leite, J. V., Scott-Murray, A. & Maidment, S. C. R. (2023). The osteology of the holotype of the British iguanodontian dinosaur *Mantellisaurus atherfieldensis*. *Monographs of the Palaeontographical Society* **665**, 1–63.

Brassey, C. A., Maidment, S. C. R. & Barrett, P. M. (2015). Body mass estimates of an exceptionally complete *Stegosaurus* (Ornithischia: Thyreophora): comparing volumetric and linear bivariate mass estimation methods. *Biology Letters* **11**, 20140984.

Breeden III, B. T., Raven, T. J., Butler, R. J., Rowe, T. B., & Maidment, S. C. (2021). The anatomy and palaeobiology of the early armoured dinosaur *Scutellosaurus lawleri* (Ornithischia: Thyreophora) from the Kayenta Formation (Lower Jurassic) of Arizona.*Royal Society Open Science* **8**, 201676.

Bussert, R., Heinrich, W. D., & Aberhan, M. (2009). The Tendaguru formation (Late Jurassic to Early Cretaceous, southern Tanzania): definition, palaeoenvironments, and sequence stratigraphy. *Fossil Record* **12**, 141-174.

Button, D. J., Porro, L. B., Lautenschlager, S., Jones, M. E. H. & Barrett, P. M. (2023). Multiple pathways to herbivory underpinned deep divergences in ornithischian evolution. *Current Biology* **33**, 557–565.

Carballido, J. L., Pol, D., Otero, A., Cerda, I. A., Salgado, L., Garrido, A. C., Ramezani, J., Cúneo, N. R. & Krause, J. M. (2017). A new giant titanosaur sheds light on body mass evolution among sauropod dinosaurs. *Proceedings of the Royal Society B: Biological Sciences* **284**, 20171219.

Carrano, M. T. & Hutchinson, J. R. (2002). Pelvic and hindlimb musculature of *Tyrannosaurus rex* (Dinosauria: Theropoda). *Journal of Morphology* **253**, 207–228.

Carrano, M. T. (1998). *Locomotor evolution in the Dinosauria: functional morphology, biomechanics, and modern analogs*. Doctoral dissertation, University of Chicago.

Carrano, M.T., Benson, R.B. and Sampson, S.D., 2012. The phylogeny of Tetanurae (Dinosauria: Theropoda). J*ournal of Systematic Palaeontology* **10**, 211–300.

Cerda, I. A., Novas, F. E., Carballido, J. L. & Salgado, L. (2022). Osteohistology of the hyperelongate hemispinous processes of *Amargasaurus cazaui* (Dinosauria: Sauropoda): Implications for soft tissue reconstruction and functional significance. *Journal of Anatomy* **240**, 1005–1019.

Chure, D. J., & Loewen, M. A. (2020). Cranial anatomy of *Allosaurus jimmadseni*, a new species from the lower part of the Morrison Formation (Upper Jurassic) of Western North America. *PeerJ* **8**, e7803.

Cilliers, C. D., Tucker, R. T., Crowley, J. L., & Zanno, L. E. (2021). Age constraint for the Moreno Hill Formation (Zuni Basin) by CA-TIMS and LA-ICP-MS detrital zircon geochronology. *PeerJ* **9**, e10948.

Clauss, M., Nurutdinova, I., Meloro, C., Gunga, H., Jiang, D., Koller, J., Herkner, B., Sander, P. M. & Hellwich O. (2016). Reconstruction of body cavity volume in terrestrial tetrapods. *Journal of Anatomy* **230**, 325–336.

Coram, R. A., Radley, J. D., & Martill, D. M. (2017). A Cretaceous calamity? The *Hypsilophodon* Bed of the Isle of Wight, southern England. *Geology Today* **33**, 66–70.

Currie, P. J. (2003). Cranial anatomy of tyrannosaurid dinosaurs from the Late Cretaceous of Alberta, Canada. *Acta Palaeontologica Polonica* **48**, 191–226.

Curry-Rogers, K. (2009). The postcranial osteology of *Rapetosaurus krausei* (Sauropoda: Titanosauria) from the Late Cretaceous of Madagascar. *Journal of vertebrate Paleontology* **29**, 1046–1086.

Cuthbertson, R. S. & Holmes, R. B. (2010). The first complete description of the holotype of *Brachylophosaurus canadensis* Sternberg, 1953 (Dinosauria: Hadrosauridae) with comments on intraspecific variation. *Zoological Journal of the Linnean Society* **159**, 373–397.

D’Emic, M. D., & Foreman, B. Z. (2012). The beginning of the sauropod dinosaur hiatus in North America: insights from the Lower Cretaceous Cloverly Formation of Wyoming. *Journal of Vertebrate Paleontology* **32**, 883–902.

D’Emic, M. D., Foreman, B. Z., Jud, N. A., Britt, B. B., Schmitz, M., & Crowley, J. L. (2019). Chronostratigraphic revision of the Cloverly Formation (Lower Cretaceous, Western Interior, USA). *Bulletin of the Peabody Museum of Natural History* **60**, 3–40

D’Emic, M. D., Melstrom, K. M., & Eddy, D. R. (2012). Paleobiology and geographic range of the large-bodied Cretaceous theropod dinosaur *Acrocanthosaurus atokensis*. *Palaeogeography, Palaeoclimatology, Palaeoecology* **333**, 13–23.

Dashzeveg, D., Dingus, L., Loope, D. B., Swisher, C. C., Dulam, T., & Sweeney, M. R. (2005). New stratigraphic subdivision, depositional environment, and age estimate for the Upper Cretaceous Djadokhta Formation, southern Ulan Nur Basin, Mongolia. *American Museum Novitates* **2005**, 1–31.

Dempsey, M., Maidment, S. C. R., Hedrick, B. P., & Bates, K. T. (2023). Convergent evolution of quadrupedality in ornithischian dinosaurs was achieved through disparate forelimb muscle mechanics. *Proceedings of the Royal Society B: Biological Sciences* **290**, 20222435.

Digitization Program Office (DPO) (2019*a*). *Triceratops horridus*. Smithsonian Institution. Online Dataset. <https://3d.si.edu/object/3d/triceratops-horridus-marsh-1889%3Ad8c623be-4ebc-11ea-b77f-2e728ce88125>

Digitization Program Office (DPO) (2019*b*). *Tyrannosaurus rex*. Smithsonian Institution. Online Dataset. <https://3d.si.edu/object/3d/tyrannosaurus-rex:d8c62d28-4ebc-11ea-b77f-2e728ce88125>

El Ouali, M., Kabiri, L., Essafraoui, B., Charroud, A., Krencker, F. N., & Bodin, S. (2021). Stratigraphic and geodynamic characterization of Jurassic–Cretaceous “red beds” on the Msemrir-Errachidia E-W transect (central High Atlas, Morocco). *Journal of African Earth Sciences* **183**, 104330.

Forster, C. A. (1990). The postcranial skeleton of the ornithopod dinosaur *Tenontosaurus tilletti*. *Journal of Vertebrate Paleontology* **10**, 273–294.

Fowler, D. W. (2017). Revised geochronology, correlation, and dinosaur stratigraphic ranges of the Santonian-Maastrichtian (Late Cretaceous) formations of the Western Interior of North America. *PLOS One* **12**, e0188426.

Galli, K. G., Buchwaldt, R., Lucas, S. G., & Tanner, L. (2018). New chemical abrasion thermal ionization mass spectrometry dates from the Brushy Basin Member, Morrison Formation, western Colorado: implications for dinosaur evolution. *The Journal of Geology* **126**, 473–486.

Gallina, P. A., Canale, J. I., & Carballido, J. L. (2021). The earliest known titanosaur sauropod dinosaur. *Ameghiniana* **58**, 35–51.

Galton, P. M. (1974). The ornithischian dinosaur *Hypsilophodon* from the Wealden of the Isle of Wight. *Bulletin of the British Museum (Natural History), Geology* **25**, 1–152.

Gilmore, C. W. (1936). *Osteology of Apatosaurus, with species reference to specimens in the Carnegie Museum*. Pittsburgh: Carnegie Institute.

Gradstein F. M., Ogg, J. G., Schmitz, M. D. & Ogg, G . M. (2020) *Geologic Time Scale 2020.* Amsterdam, Netherland*s.* Elsevier.

Han, F. L., Forster, C. A., Clark, J. M., & Xu, X. (2016). Cranial anatomy of *Yinlong downsi* (Ornithischia: Ceratopsia) from the Upper Jurassic Shishugou Formation of Xinjiang, China. *Journal of Vertebrate Paleontology* **36**, e1029579.

He, X., K. Li, & K. Cai. (1988). *The Middle Jurassic dinosaur fauna from Dashanpu, Zigong, Sichuan, vol. IV: sauropod dinosaurs (2): Omeisaurus tianfuensis*. Chengdu: Sichuan Scientific and Technological Publishing House. 1–143.

He, X., K. Li, & K. Cai. (1988). *The Middle Jurassic dinosaur fauna from Dashanpu, Zigong, Sichuan, vol. IV: sauropod dinosaurs (2): Omeisaurus tianfuensis*. Chengdu: Sichuan Scientific and Technological Publishing House.

Hedrick, B. P., Zanno, B. P., Wolfe, D. G. & Dodson, P. (2015). The slothful claw: osteology and taphonomy of *Nothronychus mckinleyi* and *N. graffami* (Dinosauria: Theropoda) and anatomical considerations for derived Therizinosaurids. *PLOS One* **10**, e0129449.

Henderson, D. M. & Snively, E. (2004). *Tyrannosaurus* en pointe: allometry minimized rotational inertia of large carnivorous dinosaurs. *Proceedings of the Royal Society of London. Series B: Biological Sciences* **271**, S57–S60.

Henderson, D. M. (2003). Footprints, trackways, and hip heights of bipedal dinosaurs – testing hip height predictions with computer models. *Ichnos* **10**, 99-114.

Hendrickx, C., Hartman, S. A., & Mateus, O. (2015). An overview of non-avian theropod discoveries and classification.*PalArch's Journal of Vertebrate Palaeontology* **12,** 1–73.

Holland, W. J. (1905). The osteology of *Diplodocus* Marsh with special reference to the restoration of the skeleton of *Diplodocus carnegiei* Hatcher, presented by Mr. Andrew Carnegie to the British Museum. *Memoirs of the Carnegie Museum* **6**, 225–278.

Holmes, R. B. (2014). The postcranial skeleton of *Vagaceratops irvinensis* (Dinosauria, Ceratopsidae). *Vertebrate Anatomy Morphology Palaeontology* **1**, 1–21.

Holwerda, F. M., & Pol, D. (2018). Phylogenetic analysis of Gondwanan basal eusauropods from the Early-Middle Jurassic of Patagonia, Argentina. *Spanish Journal of Palaeontology* **33**, 289.

Hone, D. W. E., Persons, W. S. & Le Comber, S. C. (2021). New data on tail lengths and variation along the caudal series in the non-avialan dinosaurs. *PeerJ* **9**, e10721.

Joeckel, R. M., Ludvigson, G. A., Möller, A., Hotton, C. L., Suarez, M. B., Suarez, C. A., Sames, B., Kirkland, J. I. & Hendrix, B. (2020). Chronostratigraphy and terrestrial palaeoclimatology of Berriasian-Hauterivian strata of the Cedar Mountain Formation, Utah, USA. *Geological Society, London, Special Publications* **498**, 75–100.

Kent, D. V., Santi Malnis, P., Colombi, C. E., Alcober, O. A., & Martínez, R. N. (2014). Age constraints on the dispersal of dinosaurs in the Late Triassic from magnetochronology of the Los Colorados Formation (Argentina). *Proceedings of the National Academy of Sciences* **111**, 7958–7963.

Kinneer, B., Carpenter, K., & Shaw, A. (2016). Redescription of *Gastonia burgei* (Dinosauria: Ankylosauria, Polacanthidae), and description of a new species. *Neues Jahrbuch für Geologie und Paläontologie-Abhandlungen* **282**, 37–80.

Kirkland, J. I. & Wolfe, D. G. (2001). First definitive therizinosaurid (Dinosauria; Theropoda) from North America. *Journal of Vertebrate Paleontology* **21**, 410–414.

Kirkland, J. I. & Wolfe, D. G. (2001). First Definitive Therizinosaurid (Dinosauria; Theropoda) from North America. *Journal of Vertebrate Paleontology* **21**, 410–414.

Lacerda, M. B., Bittencourt, J. S., & Hutchinson, J. R. (2023). Macroevolutionary patterns in the pelvis, stylopodium and zeugopodium of megalosauroid theropod dinosaurs and their importance for locomotor function. *Royal Society Open Science* **10**, 230481.

Lallensack, J. N., Teschner, E., Pabst, B., & Sander, M. P. (2021). New skulls of the basal sauropodomorph *Plateosaurus trossingensis* from Frick, Switzerland: is there more than one species? *Acta Palaeontologica Polonica* **66**, 1–28

Lambe, L. M. (1914). On a new genus and species of carnivorous dinosaur from the Belly River Formation of Alberta, with a description of the skull of *Stephanosaurus marginatus* from the same horizon. *The Ottawa Naturalist* **28**, 13–21.

Langer, M. C., Ramezani, J., & Da Rosa, Á. A. (2018). U-Pb age constraints on dinosaur rise from south Brazil. *Gondwana Research* **57**, 133–140.

Lautenschlager (2015). Estimating cranial musculoskeletal constraints in theropod dinosaurs. *Royal Society Open Science* **2**, 150495.

Leanza, H.A., Apesteguıa, S., Novas, F.E. & de la Fuente, M.S. (2004). Cretaceous terrestrial beds from the Neuquén Basin (Argentina) and their tetrapod assemblages. *Cretaceous Research* **25**, 61–87.

Macaulay, S., Hoehfurtner, T., Cross, S. R. R., Marek, R. D., Hutchinson, J. R., Schachner, E. R., Maher, A. E. & Bates, K. T. (2023). Decoupling body shape and mass distribution in birds and their dinosaurian ancestors. *Nature Communications* **14**, 1575.

Madzia, D., Arbour, V. M., Boyd, C. A., Farke, A. A., Cruzado-Caballero, P., & Evans, D. C. (2021). The phylogenetic nomenclature of ornithischian dinosaurs. *PeerJ* **9**, e12362.

Maidment, S. C. R. & Barrett, P. M. (2011). A new specimen of *Chasmosaurus belli* (Ornithischia: Ceratopsidae), a revision of the genus, and the utility of postcrania in the taxonomy and systematics of ceratopsid dinosaurs. *Zootaxa* **2963**, 1–47.

Maidment, S. C. R., Brassey, C. A. & Barrett, P. M. (2015). The postcranial skeleton of an exceptionally complete individual of the plated dinosaur *Stegosaurus stenops* (Dinosauria: Thyreophora) from the Upper Jurassic Morrison Formation of Wyoming, U.S.A. *PLOS One* **10**, e0138352.

Maidment, S. C., Strachan, S. J., Ouarhache, D., Scheyer, T. M., Brown, E. E., Fernandez, V., Johanson, Z., Raven, T. J. & Barrett, P. M. (2021). Bizarre dermal armour suggests the first African ankylosaur. *Nature Ecology & Evolution* **5**, 1576–1581.

Mallison, H. (2010). The digital *Plateosaurus* I: Body mass, mass distribution and posture assessed Using CAD and CAE on a digitally mounted complete skeleton. *Palaeontologia Electronica* **13**, 8A.

Mao, L., Xing, L., Zhang, J., Wang, T., & Wang, D. (2019). Revisiting the world famous Lufeng Formation dinosaur fauna: new approaches to old problems.*Historical Biology* **32**, 1062–1070.

Marsh, A. D. (2018). *Contextualizing the evolution of theropod dinosaurs in western North America using U-Pb geochronology of the Chinle Formation and Kayenta Formation on the Colorado Plateau*. Doctoral dissertation, The University of Texas.

Marsh, A. D., & Rowe, T. B. (2020). A comprehensive anatomical and phylogenetic evaluation of *Dilophosaurus wetherilli* (Dinosauria, Theropoda) with descriptions of new specimens from the Kayenta Formation of northern Arizona. *Journal of Paleontology* **94**, 1–103.

Marsicano, C. A., Irmis, R. B., Mancuso, A. C., Mundil, R., & Chemale, F. (2016). The precise temporal calibration of dinosaur origins. *Proceedings of the National Academy of Sciences* **113**, 509–513.

Martin, T., Averianov, A. O., & Pfretzschner, H. U. (2010). Mammals from the late Jurassic Qigu formation in the southern Junggar basin, Xinjiang, northwest China. Palaeobiodiversity and Palaeoenvironments, 90, 295-319.

Martínez, R. D., Lamanna, M. C., Novas, F. E., Ridgely, R. C., Casal, G. A., Martínez, J. E., Vita, J. R. & Witmer, L. M. (2016). A basal lithostrotian titanosaur (Dinosauria: Sauropoda) with a complete skull: implications for the evolution and paleobiology of Titanosauria. *PLOS One* **11**, e0151661.

Maryańska T. & Osmólska H. (1974). Pachycephalosauria, a new suborder of ornithischian dinosaurs. *Palaeontologia Polonica* **30**, 45–102.

McIntosh, J. S., & Carpenter, K. (1998). The holotype of *Diplodocus longus*, with comments on other specimens of the genus. *Modern Geology* **23**, 85–110.

Moore, A. J., Barrett, P. M., Upchurch, P., Liao, C. C., Ye, Y., Hao, B., & Xu, X. (2023). Re-assessment of the Late Jurassic eusauropod *Mamenchisaurus sinocanadorum* Russell and Zheng, 1993, and the evolution of exceptionally long necks in mamenchisaurids. *Journal of Systematic Palaeontology***21**, 2171818.

Moore, B. R. S., Roloson, M. J., Currie, P. J., Ryan, M. J., Patterson, R. T. & Mallon, J. C. (2022). The appendicular myology of *Stegoceras validum* (Ornithischia: Pachycephalosauridae) and implications for the head-butting hypothesis. *PLOS ONE* **17**, e0268144.

Norman, D. B. (1980) On the ornithischian dinosaur *Iguanodon bernissartensis* from the Lower Cretaceous of Bernissart (Belgium). *Mémoires - Institut royal des sciences naturelles de Belgique* **178**, 7–83.

Norman, D. B. (1986). On the anatomy of *Iguanodon atherfieldensis* (Ornithischia: Ornithopoda). *Bulletin de l'Institut Royal des Sciences Naturelles de Belgique Sciences de la Terre* **56**, 281–372

Norman, D. B. (2011). On the osteology of the lower Wealden (Valanginian) ornithopod *Barilium dawsoni* (Iguanodontia: Styracosterna). *Special Papers in Palaeontology* **86**, 165–194.

Ostrom, J. H. (1969). Osteology of *Deinonychus antirrhopus*, an Unusual Theropod from the Lower Cretaceous of Montana. *Bulletin of the Peabody Museum of Natural History* **30**, 1–165.

Ostrom, J. H. (1970).*Stratigraphy and paleontology of the Cloverly Formation (Lower Cretaceous) of the Bighorn basin area, Wyoming and Montana (Vol. 35)*. New Haven, USA: Yale University Press. 1–354.

Piechowski, R. & Dzik, J. (2010) The axial skeleton of *Silesaurus opolensis*. *Journal of Vertebrate Paleontology* **30**, 1127–1141.

Piechowski, R. & Dzik, J. (2010) The axial skeleton of *Silesaurus opolensis*. *Journal of Vertebrate Paleontology* **30**, 1127–1141.

Pol, D., Ramezani, J., Gomez, K., Carballido, J. L., Carabajal, A. P., Rauhut, O. W. M., Escapa, I. H. & Cúneo, N. R. (2020). Extinction of herbivorous dinosaurs linked to Early Jurassic global warming event. *Proceedings of the Royal Society B* **287**, 20202310.

Pretto, F. A., Müller, R. T., Moro, D., Garcia, M. S., Neto, V. D. P., & Da Rosa, Á. A. S. (2022). The oldest South American silesaurid: new remains from the Middle Triassic (Pinheiros-Chiniquá Sequence, *Dinodontosaurus* Assemblage Zone) increase the time range of silesaurid fossil record in southern Brazil. *Journal of South American Earth Sciences* **120**, 104039.

Prieto-Marquez, A. (2001). Osteology and variation of *Brachylophosaurus canadensis* (Dinosauria, Hadrosauridae) from the Upper Cretaceous Judith River formation of Montana. Master of Science Dissertation: Montana State University.

Ramezani, J., Fastovsky, D. E., & Bowring, S. A. (2014). Revised chronostratigraphy of the lower Chinle Formation strata in Arizona and New Mexico (USA): high-precision U-Pb geochronological constraints on the Late Triassic evolution of dinosaurs. *American Journal of Science* **314**, 981–1008.

Rauhut, O. W., & López-Arbarello, A. (2009). Considerations on the age of the Tiouaren Formation (Iullemmeden Basin, Niger, Africa): implications for Gondwanan Mesozoic terrestrial vertebrate faunas. *Palaeogeography, Palaeoclimatology, Palaeoecology* **271**, 259–267.

Rauhut, O. W., & Pol, D. (2019). Probable basal allosauroid from the early Middle Jurassic Cañadón Asfalto Formation of Argentina highlights phylogenetic uncertainty in tetanuran theropod dinosaurs.*Scientific reports* **9**, 18826.

Rauhut, O. W., Foth, C., & Tischlinger, H. (2018). The oldest *Archaeopteryx* (Theropoda: Avialae): a new specimen from the Kimmeridgian/Tithonian boundary of Schamhaupten, Bavaria. *PeerJ* **6**, e4191.

Raven, T. J., Barrett, P. M., Joyce, C. B., & Maidment, S. C. (2023). The phylogenetic relationships and evolutionary history of the armoured dinosaurs (Ornithischia: Thyreophora). *Journal of Systematic Palaeontology* **21**, 2205433.

Ren, X., Sekiya, T., Wang, T. Yang, Z. & You, H. (2021). A revision of the referred specimen of *Chuanjiesaurus anaensis* Fang *et al.*, 2000: a new early branching mamenchisaurid sauropod from the Middle Jurassic of China. *Historical Biology* **33**, 1872–1887.

Royo-Torres, R., Cobos, A., Mocho, P., & Alcalá, L. (2021). Origin and evolution of turiasaur dinosaurs set by means of a new ‘Rosetta’ specimen from Spain. *Zoological Journal of the Linnean Society* **191**, 201–227.

Ruiz-Omeñaca, J. I., Pereda Suberbiola, X., Galton, P. M., & Carpenter, K. (2006). *Callovosaurus leedsi*, the earliest dryosaurid dinosaur (Ornithischia: Euornithopoda) from the Middle Jurassic of England. In *Horns and beaks: ceratopsian and ornithopod dinosaurs* (ed. Carpenter, K.), 3–16. Bloomington, USA: Indiana University Press.

Russell, D. A. (1970). Tyrannosaurs from the Late Cretaceous of Western Canada. *National Museum of Natural Sciences Publications in Palaeontology* **1**, 1–30.

Sales, M. A. F., Liparini, A., de Andrade, M. B., Aragão, P. R. L. & Schultz, C. L. (2017). The oldest South American occurrence of Spinosauridae (Dinosauria, Theropoda). *Journal of South American Earth Sciences* **74**, 83–88.

Salgado, L., Canudo, J. I., Garrido, A. C., Moreno-Azanza, M., Martínez, L. C., Coria, R. A., & Gasca, J. M. (2017). A new primitive Neornithischian dinosaur from the Jurassic of Patagonia with gut contents. *Scientific Reports* **7**, 1–10.

Schweigert, G. (2007). Ammonite biostratigraphy as a tool for dating Upper Jurassic lithographic limestones from South Germany: first results and open questions. Neues Jahrbuch für Geologie und Paläontologie. Abhandlungen **245**, 117.

Sellers, W. I. & Pond, S. B. (2015). Kinect controlled dinosaur simulations for education and public outreach. PeerJ PrePrints **3**, e1584v1.

Sereno, P. C. Myhrvold, N., Henderson, D. M., Fish, F. E., Vidal, D., Baumgart, S. L., Keillor, T. M., Formoso, K. K., Conroy, L. L. (2022). *Spinosaurus* is not an aquatic dinosaur. *eLife* **11**, e80092.

Sereno, P. C., Beck, A. L., Dutheil, D. B., Gado, B., Larsson, H. C., Lyon, G. H., Marcot, J. D., Rauhut, O. W. M., Sadleir, R. W., Sidor, C. A., Varricchio, D. D., Wilson, G. P. & Wilson, J. A. (1998). A long-snouted predatory dinosaur from Africa and the evolution of spinosaurids. *Science* **282**, 1298–1302.

Smith, D. K. & Gillette, D. D. (2023). Reconstruction of soft noncontractile tissue in the derived therizinosaur Nothronychus: The interplay of soft tissue and stress on hindlimb ossification and posture. *Journal of Morphology* **284**, e21579.

Stevens, K. A. (2013). The Articulation of Sauropod Necks: Methodology and Mythology. *PLoS ONE* **8**, e78572.

Tschopp, E., & Mateus, O. (2013). The skull and neck of a new flagellicaudatan sauropod from the Morrison Formation and its implication for the evolution and ontogeny of diplodocid dinosaurs. *Journal of Systematic Palaeontology* **11**, 853–888.

Tsuihiji, T. (2010). Reconstructions of the axial muscle insertions in the occipital region of dinosaurs: evaluations of past hypotheses on Marginocephalia and Tyrannosauridae using the extant phylogenetic bracket approach. *The Anatomical Record* **8**, 1360–1369.

Turner, C. E., & Peterson, F. (1999). Biostratigraphy of dinosaurs in the Upper Jurassic Morrison Formation of the western interior, USA. In *Vertebrate paleontology in Utah Vol. 99*, 77–114. Salt Lake City, Utah: Utah Geological Survey Miscellaneous Publication.

Wang, J., Ye, Y., Pei, R., Tian, Y., Feng, C., Zheng, D., & Chang, S. C. (2018). Age of Jurassic basal sauropods in Sichuan, China: a reappraisal of basal sauropod evolution. *GSA Bulletin* **130**, 1493–1500.

Wills, S., Underwood, C. J., & Barrett, P. M. (2023). Machine learning confirms new records of maniraptoran theropods in Middle Jurassic UK microvertebrate faunas. P*apers in Palaeontology* **9**, e1487.

Xu, X., Zhou, Z., Sullivan, C., Wang, Y., & Ren, D. (2016). An updated review of the Middle‐Late Jurassic Yanliao Biota: chronology, taphonomy, paleontology and paleoecology. *Acta Geologica Sinica‐English Edition* **90**, 2229–2243.

Yans, J., Dejax, J., & Schnyder, J. (2012). On the age of the Bernissart Iguanodons. Bernissart dinosaurs and early Cretaceous terrestrial ecosystems. In *Bernissart dinosaurs and early Cretaceous terrestrial ecosystems* (ed. Godefroit, P.) 79–86. Bloomington, USA: Indiana University Press.

Yong, Y. E., Hui, O. Y. G. & Qian-Ming, F. U. (2001). New material of *Mamenchisaurus hochuanensis* from Zigong, Sichuan. *Vertebrata PalAsiatica* **39**, 266.

Young, C. C., & Zhao, X. (1972). *Mamenchisaurus*. Institute of Vertebrate Paleontology and Paleoanthropology Monograph Series I, No. 8 (Translated by Will Downs, 1996 Bilby Research Center, Northern Arizona University).

Yu, Z., Wang, M., Li, Y., Deng, C., & He, H. (2021). New geochronological constraints for the Lower Cretaceous Jiufotang Formation in Jianchang Basin, NE China, and their implications for the late Jehol Biota. *Palaeogeography, Palaeoclimatology, Palaeoecology* **583**, 110657.

Zaher, H., Pol, D., Carvalho, A. B., Nascimento, P. M., Riccomini, C., Larson, P., Juarez-Valieri, R., Pires-Domingues, R., da Silva Jr., N. J. & de Almeida Campos, D. (2011). A complete skull of an Early Cretaceous sauropod and the evolution of advanced titanosaurians. *PLOS One* **6**, e16663.
